# Mixed guanine, adenine base quartets: possible roles of protons and metal ions in their stabilization

**DOI:** 10.1007/s00775-017-1507-7

**Published:** 2017-12-07

**Authors:** Dominik A. Megger, Patrick M. Lax, Jeroen Paauwe, Célia Fonseca Guerra, Bernhard Lippert

**Affiliations:** 1 Medizinisches Proteom-Center, Ruhr-Universität Bochum, 44801 Bochum, Germany; 2 Institute of Virology, University Hospital, University Duisburg-Essen, 45120 Essen, Germany; 3 Fakultät Chemie und Chemische Biologie (CCB), Technische Universität Dortmund, 44221 Dortmund, Germany; 4 Department of Theoretical Chemistry and Amsterdam Center for Multiscale Modeling (ACMM), VU Amsterdam, De Boelelaan 1083, 1081 HV Amsterdam, The Netherlands; 5 Gorlaeus Laboratories, Leiden Institute of Chemistry, Leiden University, P.O. Box 9502, 2300 RA Leiden, The Netherlands

**Keywords:** Mixed adenine, Guanine quartets, Protonated adenine, Alkali metal ions, DFT calculations

## Abstract

Structural variations of the well-known guanine quartet (G_4_) motif in nucleic acid structures, namely substitution of two guanine bases (G) by two adenine (A) nucleobases in mutual *trans* positions, are discussed and studied by density functional theory (DFT) methods. This work was initiated by three findings, namely (1) that GA mismatches are compatible with complementary pairing patterns in duplex-DNA structures and can, in principle, be extended to quartet structures, (2) that GA pairs can come in several variations, including with a N1 protonated adeninium moiety (AH), and (3) that cross-linking of the major donor sites of purine nucleobases (N1 and N7) by transition metal ions of linear coordination geometries produces planar purine quartets, as demonstrated by some of us in the past. Here, possible structures of mixed AGAG quartets both in the presence of protons and alkali metal ions are discussed, and in particular, the existence of a putative four-purine, two-metal motif.

**Electronic supplementary material:**

The online version of this article (10.1007/s00775-017-1507-7) contains supplementary material, which is available to authorized users.

## Introduction

The field of structural nucleic acid chemistry is experiencing an ever increasing number of novel unconventional structures beyond the by now classical features of duplex DNA. These include, among others, triplex structures based on nucleobase triplets, quadruplex structures based on nucleobase quartets, and several larger multi-stranded arrays, as well as special folding patterns of polynucleotides, junctions, different strand orientations, etc. ([1], and refs. cited). This research is fuelled in particular by questions regarding their biological relevance and numerous applications in nucleic acid nanotechnology [2–5]. Without exception, the stabilization of classical and unconventional nucleic acid structures relies on the presence of “helpers” such as proteins, small molecules, cations in general and metal ions in particular, or simply protons. From a bioinorganic chemistry perspective, the role of metal ions and of protons is of particular interest [6–8]. It appears that at present this field is dominated by research surrounding guanine (G)-rich sequences and in particular G_4_ quadruplex structures [4, 5], hemiprotonated cytosine (C)-rich sequences (“i-motif“) [9], as well as biomaterial scaffolds based on DNA [10, 11]. In the G_4_ structures, four guanine bases are arranged in a cyclic fashion via eight hydrogen bonds involving the Hoogsteen face (N7, O6) and part of the Watson–Crick face (N1H, N2H_2_) of each G, with a metal ion in the center or sandwiched between two G_4_ layers [12]. In the i-motif, hemiprotonated cytosine pairs ([CHC^+^]) are interdigitated into each other, combining parallel strand orientation within a pair and antiparallel orientation between adjacent pairs [13]. Among DNA-based scaffolds, 3D lattices can be constructed from suitable sequences which allow for conventional Watson–Crick helices and orthogonally extending regions forming homo base pairs (AA, GG, [CHC^+^]) and hence adopting a parallel strand orientation [14].

Here we focus on the potential of adenine (A) and guanine to form mixed AGAG tetrads in the absence or presence of alkali metal ions and/or under low pH conditions, when A becomes protonated (AH^+^). The motivation for this study comes from our earlier findings that a G(AH)(AH)G quartet can be crystallized by employing model nucleobases [15] and that metal ions capable of cross-linking purine bases via N7 and/or N1 sites enable the construction of metalated purine quartets or fragments thereof, with additional inter-nucleobase hydrogen bonds possible [16–23]. More recent findings on the existence of G_3_ triplets layered on G_4_, without [24] or with a water molecule substituting for the fourth G [25] and providing a site for binding another flat molecule, as well as mixed G_2_, xanthine, 8-oxoguanine quartets [26, 27] justify our approach.

We are aware that the likelihood of a mixed AG quartet to occur within a biologically relevant quadruplex structure, based essentially on a flat structure derived from model compounds and relevant calculations, is not stringent. On the other hand, feasible sequences for quadruplex structures consisting of 10 G’s and 2 A’s are numerous, in principle. For example, a sequence 5′-…**A**GG(TTA)GGG(TTA)GG**A**(TTA)GGG… (loops in brackets, subject to variations) could feasibly lead to a quadruplex with two A’s in mutual *trans*-positions, sitting on top of two layers of regular G_4_ quartets. Moreover, the well-established propensity of adenine to engage in base stacking, after all most pronounced of all common nucleobases, could be a distinct advantage for such a structure. It should be noted that there is evidence that at least in one case, that of a repressor element of a human growth factor, a G_3_A element within a three-storey G-quadruplex has been identified [28].

In the following, we wish to elaborate on the structures and relative energies of feasible GAGA and G(AH)G(AH) quartets in the absence and presence of metal ions, be they biologically relevant (alkali metal ions) or not (transition metal ions of linear coordination geometry). Concentration will be on geometries with identical purines *trans* to each other; hence G–G hydrogen bonding contacts as seen in G_4_ as well as in G_3_ or G_2_ fragments thereof, will not be considered. A major focus will be on the question, whether or not more than a single metal cation, as seen in G_4_ structures, is capable of stabilizing such mixed purine quartet structures.

## Computational details

The computational models on purine quartets are based on X-ray crystal structures of known AG base pairs as well as metal-modified adducts. All calculations were performed with the Amsterdam Density Functional (ADF) program [29] using dispersion-corrected relativistic density functional theory (DFT) at the ZORA-BLYP-D3/TZ2P level for geometry optimizations and ZORA-BLYP-D3/QZ4P level for energies. These levels of theory have shown to adequately predict structures and energies of guanine quartets and quadruplexes [30–33]. Full computational details are available in the Supplementary Information.

To compare the stabilities of the quartet structures, the bond energy ∆*E* of each quartet is calculated using the energies of quartets and their respective monomeric components. Depending on the type of the quartet, ∆*E* is calculated as follows:

$${\text{Neutral quartet: }}\Delta E = E_{\text{quartet}} {-}2 \cdot E_{\text{guanine}} {-}2 \cdot E_{\text{adenine}}$$

$${\text{Protonated quartet: }}\Delta E = E_{\text{quartet}} {-}2 \cdot E_{\text{guanine}} {-}2 \cdot E_{\text{adeninium}}$$

$${\text{Metalated quartet: }}\Delta E = E_{\text{quartet}} {-}2 \cdot E_{\text{guanine}} {-}2 \cdot E_{\text{adenine}} {-}2 \cdot E_{{{\text{M}}^{ + } }} .$$

In protonated quartet structures, *E*_adeninium_ refers to the energy of adenine either protonated at N1 or N7. $E_{{{\text{M}}^{ + } }}$ is only considered in metalated quartets with M = Li, Na or K. For calculations in water, the solvation effects are simulated using the conductor-like screening model (COSMO) [29].

## Results and discussion

### Base pairing schemes between A and G

**Fig. 1:**
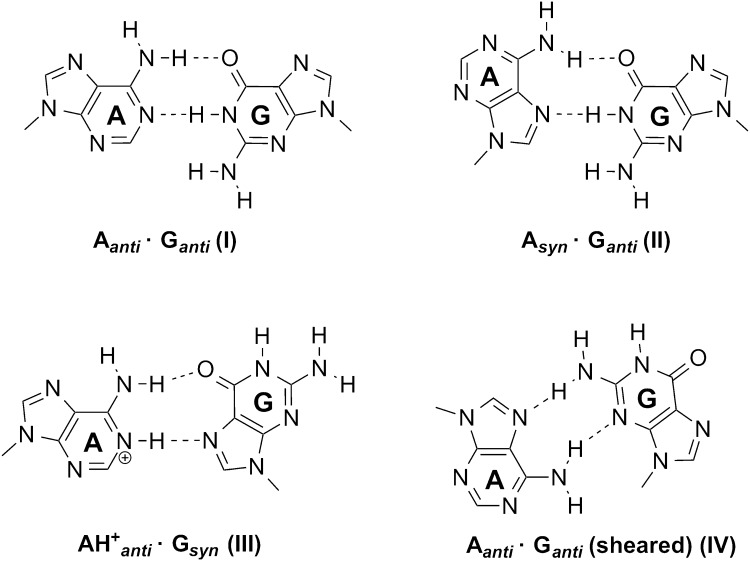
Pairing schemes between adenine and guanine nucleobases

Mismatches between adenine and guanine nucleobases are possible in DNA [34] and RNA structures [35]. The most common DNA mispairs include the combinations A_anti_·G_anti_ (I) [36], A_syn_·G_anti_ (II) [37], ${\text{AH}}^{ + }_{\text{anti}}$·G_syn_ (III) [38, 39] and the “sheared” A_anti_·G_anti_ pair (IV) [40]. As to the stability of the A·G mispairs containing neutral bases, (I) and (II) are more stable (in gas phase) than the adenine–thymine Watson–Crick pair [41]. With the exception of (IV), in none of the other three pairs the exocyclic guanine–N(2)H_2_ is group involved in hydrogen bond formation (Fig. 1). It, therefore, is not surprising to see that mismatches between adenine and inosine follow the variations observed for A·G mismatches (I)–(III) and that they, too, are surprisingly stable [42]. Mispairs (I) and (II) are moderately to strongly propeller twisted in duplex DNA [37, 38] and in the case of (I), the N(2)H_2_ group of guanine makes an unusual hydrogen bond to O(2) of an adjacent A·T pair [36]. A thorough literature search performed by Šponer et al. [43] has revealed numerous other cases of such out-of-plane H bond interactions of guanine–NH_2_ in DNA and RNA structures. On the other hand, ab initio calculations with A·G model base pairs have shown that very little energy is required to make the two nucleobases in A_anti_·G_anti_ co-planar [44].

All three mismatches (I)–(III) are, in principle, capable of forming dimers via H bond formation, hence of generating purine quartets. They may adopt the shapes of squares, rectangles or diamonds if projected on to a plane, yet may not necessarily lead to co-planar arrangements (Figures S1–S2). Their existence in multi-stranded nucleic acid structures is under dispute [45–49], with the non-planarity of the quartet derived from the A_syn_·G_anti_ pair (II) being one of the main arguments against a role in biological systems [45].

In the following, base quartets derived from the three mismatches (I)–(III) will be discussed individually, and ways leading to a more planar arrangement will be examined. Specifically, the role of protons and of alkali metal ions in modifying the topology of the purine base quartet will be studied.

### Neutral quartets derived from the A·G mispairs (I) and (II)

**Fig. 2:**
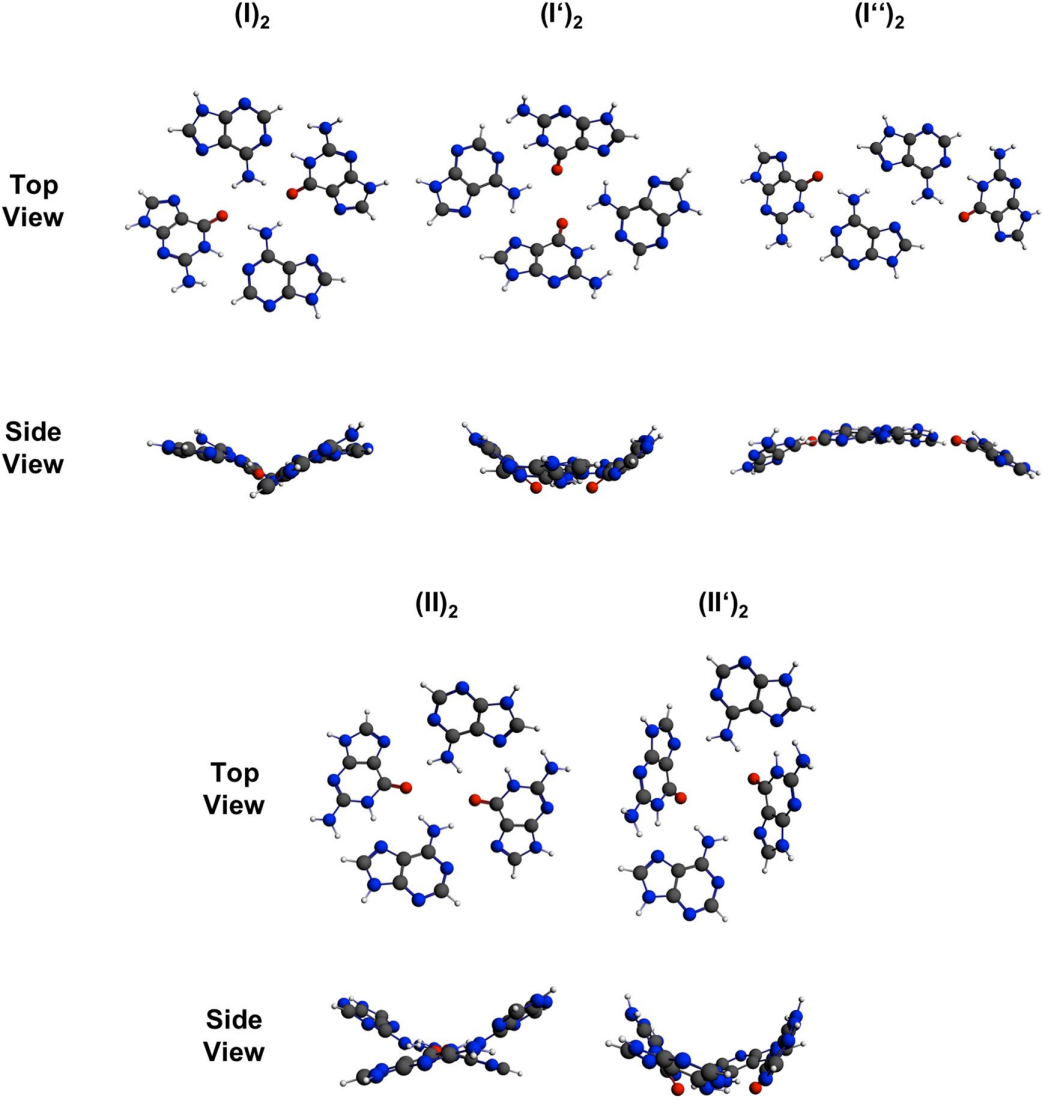
Top and side views of quartets derived from dimerization of neutral A_anti_ G_anti_ (I) and neutral A_syn_ G_anti_ (II) base pairs

Dimerization of (I) or (II) via hydrogen bonding can lead to the following nucleobase quartet arrangements: (I)_2_, (I′)_2_, or (I″)_2_ as well as (II)_2_ or (II′)_2_. According to our calculations in gas phase and water only slight deviations between structures in gas phase and solution are observed. Hence, only structures in water (Fig. 2) will be discussed in detail.

The base quartet (I)_2_ adopts a non-planar geometry. Within each A·G pair, the two bases are slightly propeller-twisted (22.6°) and the two halves of the quartet are strongly skewed (47.4° between the averaged A·G planes). Whereas the two adenines are oriented in a buckled fashion (22.5°) the two guanines are distinctly twisted (65.6°). The distance between A–N(7) and G–N(7) is 3.88 Å and it seems obvious that a non-planar geometry is adopted as a result of repulsive interactions between the electron lone pairs of these sites. The two hydrogen bonds within each pair in (I)_2_ are reversed in length relative to the isolated A_anti_·G_anti_ pair (I) with G–N(1)H…N(1)–A being 2.87 Å and G–O(6)…H_2_N(6)–A being 2.92 Å in the quartet. The inter-base pair hydrogen bond between G–O(6) and H_2_(6)N–A is similar in length (2.95 Å) but the angle is slightly buckled (147.9°). The distances between N9 atoms of opposite adenines and guanines are 12.55 and 13.53 Å, respectively. If the quartet is constrained to C_2h_ symmetry during optimization, and therefore, co-planar arrangement of the nucleobases is enforced, the A–N(7)…N(7)–G distance becomes shorter (3.50 Å). Contrarily, hydrogen bonds are increased in length by approximately 0.06 Å, except G–N(1)H…N(1)–A. The energy required for planarization of the base quartet amounts to 3.2 kcal/mol in gas phase and 5.9 kcal/mol in water.

A way of relieving the repulsion between the N7 sites in (I)_2_ is to slide the two pairs slightly past each other and to generate the diamond-shaped quartet (I′)_2_. Such a possibility has previously been proposed by Murchie and Lilley [47]. The computed structure of (I′)_2_ is shown in Fig. 2, top. Again, A and G pairs are propeller-twisted (27.5°) within the pair and, similar to the situation in (I)_2_, the two adenine bases are buckled to each other (24.9°). However, the two guanine bases are now oriented in an almost perpendicular fashion (82.9°). Hydrogen bond distances in G–N(1)H…N(1)–A and G–O(6)…H_2_N(6)–A amount to 2.87 and 2.96 Å, respectively. The length of the inter-base pair hydrogen bond between A–N(6)H_2_ and G–N(7) is 2.94 Å. In comparison to (I)_2_, the distances between N9 atoms of opposite adenines are increased (14.50 Å), whereas guanines are closer to each other (10.39 Å). Constraining the bases to a planar arrangement requires approx. 2 kcal/mol in both gas phase and water. Hydrogen bonds adjust to this structural change as follows: The intra- and inter-base pair contacts G–N(1)H…N(1)–A are elongated to 2.95 and 2.98 Å, while G–O(6)…H_2_N(6)–A shortens by about 0.08 Å.

Sliding two base pairs (I) in opposite direction leads to a situation as realized in (I″)_2_, in which the A_anti_·G_anti_ pairs interact exclusively via two hydrogen bonds between the Hoogsteen edges of adenine, hence through A–N(7) and A–N(6)H_2_. A pairing of the kind seen in (I″)_2_ is realized, for example, upon head–head dimerization of two DNA hairpins of composition d(GCATGCT) [50] and is a common association pattern of protonated adenine moieties in poly(AH^+^) [51]. The respective hydrogen bond is 2.96 Å long. Intra-base pair hydrogen bonds are 2.89 Å (G–O(6)…H_2_N(6)–A) and 2.90 Å (G–N(1)H…N(1)–A) in length. In this arrangement, the two bases within the A·G pair have a reduced propeller twist (9.4°), but the two adenines are still buckled by 20.9°. However, the angle between the planes of opposite guanines is remarkably reduced to 48.0° and the two halves of the quartets are also less skewed (21.1°). Hence, the overall structure of (I″)_2_ is considerably more planar than (I′)_2_ and (I)_2_.

Gu and Leszczynski [45] have calculated (II)_2_ and found it to be V-shaped with the G bases strongly tilted along the N7–G_1_…N7–G_2_ axis and the A bases oriented almost perpendicular to each other along this axis. In our current study, attempts to optimize the geometry of (II)_2_ in the gas phase failed and instead produced a geometry as shown in (II′)_2_. However, our calculations in water provided a similar result, with (II)_2_ adopting a non-planar orientation of the nucleobases. Here, the two opposite guanines are twisted (32.4°) and the two adenines are strongly buckled (61.4°). The angle of 46.0° between the averaged A·G planes shows that the two halves of the quartet are distinctly skewed, whereas the single bases within an A·G are twisted by 19.4°. Lengths of hydrogen bonds within the quartet range from 2.88 to 2.90 Å. Constraining this quartet to planarity require 7.0 kcal/mol in water. As a consequence of planarization, all hydrogen bonds lengthen by approx. 0.10 Å. In analogy to (I)_2_, the favored non-planar arrangement can be attributed to repulsive interaction between lone electron pairs at the nitrogen atoms, in this case A–N(1) and G–N(7) with a distance of 3.88 Å in the relaxed geometry and 3.44 Å in the constrained planar one.

In quartet (II′)_2_ a comparable, yet more distorted geometry as previously seen in (II)_2_ is observed. Overall, a bowl-shaped geometry is adopted and opposite bases are heavily buckled (adenines 48.5°, guanines 56.3°). The single A·G base pairs show a propeller twist of 31.6° and A·G planes are strongly skewed (76.3°). The distances of the hydrogen bonds G–O(6)…H_2_N(6)–A formed via Hoogsteen and Watson–Crick edges amount to 2.94 and 3.01 Å, respectively. The length of the hydrogen bond N(1)H…N(7)–A is 2.88 Å. If constrained to planarity, the hydrogen bonds involving G–O(6) are both shortened by approx. 0.10 Å, whereas G–N(1)H…N(7)–A elongates to the same extent.

The bond energies of all quartets discussed so far are summarized in Table 1. A direct comparison of the five quartets shows that in gas phase quartet (I′)_2_ is the most stable one, either in relaxed and planar geometry. In water, (I′)_2_ and (I″)_2_ are necessarily isoenergetic in relaxed geometry and (I″)_2_ is more stable by 1 kcal/mol if planarity is enforced. Overall, square-shaped quartets (I)_2_ and (II)_2_ are significantly less stable than slid variants, especially when a co-planar arrangement of the nucleobases is enforced. This can clearly be attributed to the resulting steric repulsion between the lone electron pairs of endocyclic nitrogen atoms.

**Table 1:** Bond energies (∆*E* in kcal/mol) of neutral quartets (ZORA-BLYP-D3/QZ4P), either in a relaxed structure (C_2_ symmetry) or constrained to planarity (C_2h_ symmetry)

Quartet	C_2_ symmetry		C_2h_ symmetry	
	∆*E* (gas phase)	∆*E* (water)	∆*E* (gas phase)	∆*E* (water)
(I)_2_	− 45.8	− 15.2	− 42.6	− 9.3
(I′)_2_	− 54.0	− 18.7	− 52.2	− 16.5
(I″)_2_	− 51.9	− 18.5	− 51.3	− 17.5
(II)_2_	n.d.	− 14.4	n.d.	− 7.3
(II′)_2_	− 50.1	− 17.6	− 46.3	− 13.7

Selected geometrical parameters of the quartets are provided in Table S1

### Protonation of A_anti_·G_anti_ and A_syn_·G_anti_

A second possibility of reducing the repulsion between opposite A–N(7) and G–N(7) sites in (I)_2_ and opposite A–N(1) and G–N(7) sites in (II)_2_ is to insert protons between these positions. Nucleobase proton binding even at neutral pH value is a phenomenon widely seen in nucleic acid chemistry [52], and the p*K*_a_ of AH^+^ [53] is close to that of protonated cytosine, which occurs at physiological pH. It implies that protons can be accepted if it is favorable for the generation of a stable H bonded associate. Analogous to our finding for the neutral quartets, the calculations of protonated quartets in water and gas phase gave similar structures. Hence, also in this case only structures calculated in water are discussed in detail.

**Fig. 3:**
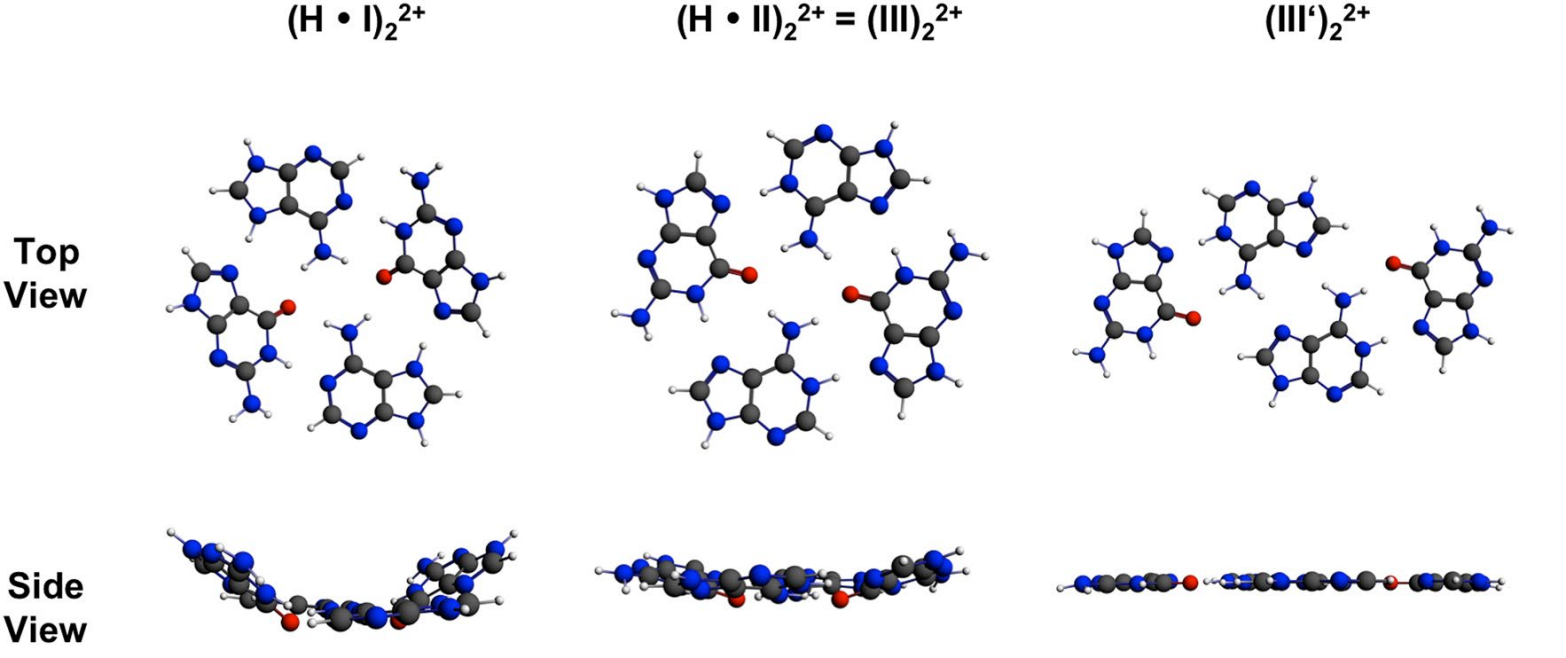
Top and side views of G(AH)(AH)G quartets. Note that A protonation sites are different in the first and the two other structures

An optimized structure of a quartet derived from two ${\text{AH}}^{ + }_{\text{anti}}$·G_anti_ pairs (H·I)_2_ is given in Fig. 3. The protons introduced at the two adenine–N(7) sites, generate rare tautomers of the adeninium cation, which normally is protonated at the N(1) site.

The geometry of (H·I)_2_ shows four distinct types of hydrogen bonds, which are all reasonably short. In contrast to the situation in neutral quartet structures, the repulsion between N7 positions of guanine and adenine is diminished due to protonation of adenine at N7. The formed hydrogen bond A–N(7)H…N(7)–G shows a distance of 2.78 Å and an angle of 168.2°. The second inter-base pair hydrogen bond is G–O(6)…H_2_N(6)–A. With a distance of 2.84 Å and an angle of 153.0° the situation is comparable to the neutral quartet (I)_2_. The distances of two hydrogen bonds formed via Watson–Crick edges, namely within the (AH)G pair, are completely inverted in comparison to (I)_2_ with A–N(6)H_2_…O(6)–G being 2.87 Å and A–N(1) and N(1)H–G 2.93 Å long.

Twofold protonation of (II)_2_, with protons residing at the N1 sites of the adenine bases produces the nucleobase quartet (H·II)_2_. As far as the geometry of the quartet and the hydrogen bonding interactions are concerned and ignoring differences in orientations of sugar residues (it needs to be pointed out that the use of *syn* and *anti* descriptions of sugar puckering as common in base pairing schemes can become misleading upon quartet formation in that a view rotated by 90° inverts relative orientations), a simple rotation by 90° converts (H·II)_2_ into quartet (III)_2_, which is obtained alternatively through dimer formation of the ${\text{AH}}^{ + }_{\text{anti}}$·G_syn_ pair (III). We have previously reported on (III)_2_ and on a variation of it, the slipped quartet (III′)_2_ [15]. The calculated structure of the quartet (III)_2_ shows also four distinct types hydrogen bonds.

An energy of only 0.7 kcal/mol in gas phase and 1.3 kcal/mol in solution is required to force the slightly bowl-shaped quartet (III)_2_ into a flat structure. (III′)_2_, on the other hand, is virtually completely planar, not only according to the calculations in gas phase and water, but also in the solid state [15]. However, compared to (III)_2_, (III′)_2_ is less stable by 6.7 kcal/mol in water and 5.9 kcal/mol in the gas phase. Table 2 provides an overview of the bond energies of all investigated protonated quartets.

**Table 2:** Bond energies (∆*E* in kcal/mol) of protonated quartets (ZORA-BLYP-D3/QZ4P), either in a relaxed structure (C_2_ symmetry) or constrained to planarity (C_2h_ symmetry)

Quartet	C_2_ symmetry		C_2h_ symmetry	
	∆*E* (gas phase)	∆*E* (water)	∆*E* (gas phase)	∆*E* (water)
(H·I)_2_	− 73.6	− 30.3	− 73.9	− 28.6
(H·II)_2_/(III)_2_	− 73.9	− 30.3	− 73.2	− 29.0
(III′)_2_	− 68.0	− 23.6	− 68.0	− 23.4

Selected geometrical parameters of the quartets are provided in Table S2

### Introducing alkali metal ions into [AG]_2_ quartets

As a third possibility of avoiding repulsion between endocyclic N atoms of the purine bases in quartet structures, the effect of alkali metal ions placed between such sites, was studied. Structures were calculated for Li^+^, Na^+^, and K^+^ starting out from (I)_2_ and (II)_2_. Depending on the introduced metal ions and the environment, namely gas phase or water, remarkably different quartet structures were obtained. Hence, differences between structures in gas phase and water will also be discussed in this section.

In the case of Na^+^ and K^+^, the introduction of two alkali metal ions into the base quartets (I)_2_ and (II)_2_ does not lead to a planarization (Figure S3). Moreover, all quartet structures containing these ions are more distorted than the respective non-metalated species. The observed distortions are most likely based on two main reasons, namely a steric interference between the relatively large K^+^ and Na^+^ ions and one of the amino protons as well as an elongation and weakening of the intra-base pair hydrogen bonds. In the gas phase, the quartets (M·I)_2_ and (M·II)_2_ containing Na^+^ or K^+^ adopt saddle-shaped structures with similar metal-binding patterns. The metal ions are bound via N(7), O(6) chelation by the guanine base (for chelation of K^+^ to G-N(7),O(6), see, e.g. [54]) as well as N(7) binding (M·I)_2_ and N(1) binding (M·II)_2_ by the adenine base, respectively. For the hydrogen bonds between and within base pairs (I) and (II), a remarkable elongation or complete disruption is observed in comparison to neutral and protonated species. The impact on the inter-base pair bond between G–O(6) and H_2_(6)N–A can be attributed to the comparatively long adjacent coordinative bonds, whereas the elongation of intra-base pair hydrogen bonds is likely driven by changes of the electronic structure of each nucleobase caused by the metal ion binding at N7. In contrast to the situation in the gas phase, the quartets (M·I)_2_ and (M·II)_2_ exhibit diverse structure types in water. Two of the four quartets, namely (K·I)_2_ and (Na·II)_2_ adopt structures in which the two base pairs are mainly interacting by π–π-stacking interaction (inter-base pairs distances of approx. 3.5 Å) instead of forming inter-base pair hydrogen bonds between G–O(6) and H_2_(6)N–A. (K·II)_2_ and (Na·I)_2_, on the other hand, form heavily distorted quartets with inter-base pair hydrogen bonds. If forced to planarity, quartets containing K^+^ and Na^+^ ions adopt rectangular structures with completely disrupted inter-base pair hydrogen bonds, hence with segregation of the quartets into two pairs. For most of the quartets, the energy necessary for a complete planarization ranges from 2.8 to 8.0 kcal/mol, depending on the type of quartet, the environment and the metal ion. However, for the stacked structures of (K·I)_2_ and (Na·II)_2_ planarization energies are considerably higher, with values ranging from 12.8 to 13.1 kcal/mol.

**Fig. 4:**
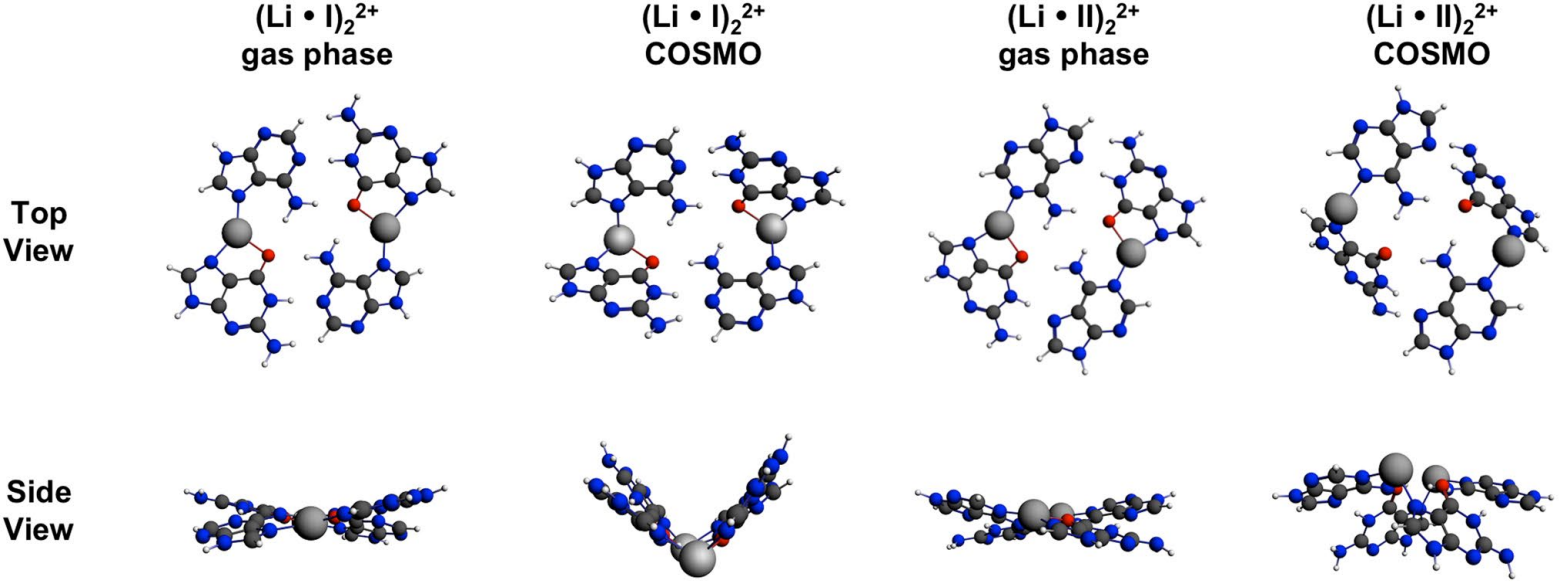
Top and side views of AGAG quartets containing two Li^+^ ions. Optimized geometries in gas phase and water are shown

In contrast to Na^+^ and K^+^ ions, the observed distortive effects are less pronounced if the relatively small Li^+^ ion is introduced as bridging metal ion (Fig. 4). In the gas phase, the quartets (Li·I)_2_ and (Li·II)_2_ both adopt almost planar structures. The metal-binding patterns in both quartets are similar to those observed for the quartets containing Na^+^ and K^+^ ions, namely N(7),O(6) chelation by the guanine base and N(7) or N(1) binding by the adenine base. Coordinative bond lengths are rather similar in both quartets ranging from 1.98 to 2.04 Å in (Li·I)_2_ and 1.99–2.08 Å in (Li·II)_2_. A remarkable difference to the quartets containing Na^+^ and K^+^ ions is realized with regard to the hydrogen bonding interactions. Due to the small size of the Li^+^ ion, the inter-base pair hydrogen bonding interactions in (Li·I)_2_ and (Li·II)_2_ are maintained, even if they are slightly elongated in comparison to the respective neutral quartets and remarkably longer than those in the protonated species. Concerning the intra-base pair hydrogen bonds, an elongation of the hydrogen bond G–O(6)…H_2_N(6)–A can be observed in both quartets, which is a direct consequence of the involvement of G–O(6) in Li^+^ binding. For the second intra-base pair hydrogen bond, namely G–N(1)H…N(1)–A (2.83 Å) in the case of (Li·I)_2_ and G–N(1)H…N(7)–A (2.88 Å) in the case of (Li·II)_2_, a slight shortening in comparison to the respective non-metalated and protonated species is realized.

In summary, the calculations have shown that among the investigated alkali metal ions Li^+^ seems to be the most suitable one for the stabilization of AGAG quartets, either in type (I) and (II) quartets. For all described quartet structures containing alkali metal ions, a detailed summary of the most important geometrical parameters like bond distances and angles, as well as energetic parameters is provided as supplementary material (Table S3).

### Introducing transition metal ions into [AG]_2_ quartets

A final option to overcome the repulsive interactions of endocyclic N atoms of the purine bases is the introduction of linearly coordinating transition metal ions such as Ag^+^ and Hg^2+^ or metal entities such as *trans*-(NH_3_)_2_Pt^II^. Metalated purine base pairs interacting through hydrogen bond formation or open and closed purine quartets containing three or more metal ions have been isolated and X-ray structurally characterized in numerous cases [16–23]. Although of interest from a conceptual point of view, we do not consider these constructs viable models for biologically relevant entities at this point.

## Conclusions

By means of DFT methods, 14 different types of mixed AGAG quartets containing either neutral, protonated, or metalated nucleobases were analyzed. In addition to energetic stability trends among different nucleobase arrangements in neutral quartets, the role of protons and alkali metal ions for the stabilization of AGAG quartets was of particular interest. Based on the reported findings, the following conclusions can be drawn:

1. Neutral AGAG tetrads preferentially adopt rectangular- or diamond-shaped structure as realized in the quartets (I′)_2_, (II′)_2_, and (I″)_2_. In contrast, the square-shaped quartets (I)_2_ and (II)_2_ are less stable due to the repulsive interaction of lone electron pairs of nitrogen atoms.
2. Protonation of nitrogen atoms represents an ideal method to overcome repulsive interactions between lone electron pairs of nitrogen atoms. The quartets (H·I)_2_ and (H·II)_2_ both adopt square-shaped structures, which are of similar stability and more stable than diamond-shaped quartet (III′)_2_. Recent reports on the reversible effect of pH as a stimulus for conformational changes of G-quadruplex DNA [55], which appear to be associated with protonation of bases in the loop regions, and specifically that of an adenine in a AGA triplet [56], are suggesting that the existence of mixed G(AH)G(AH) quartets in tetrastranded nucleic acids is not fully unrealistic.
3. The introduction of two alkali metal ions leads to a stabilization of square-shaped quartets if the relatively small Li^+^ ion is introduced. On the other hand, the introduction of K^+^ and Na^+^, respectively, leads to distorted quartet structures.
4. An analogy between artificial mixed purine quartets, in which the nucleobases are cross-linked by transition metal ions of linear coordination geometry, and alkali metal ions having a similar function, is not observed.

In conclusion, the question whether or not the here discussed AGAG tetrads and their variants involving protonated A’s are viable in stem regions of tetraplex nucleic acids cannot be answered at present. Although it is unquestioned that “guanines are a quartet’s best friends” [57], hence that natural G’s form more stable quartets than substituted G’s or other nucleobases, there is an increasing number of examples now available, which demonstrate that G’s in G4 structures can indeed be substituted by other molecules [25–27, 58], including adenine [58]. In other words, such modified entities can be tolerated in structures stabilized by additional G tetrads.

All the here discussed AGAG quartets are devoid of the four keto oxygen atoms, which can bind Na^+^ or K^+^ and which are important for the stabilization of G tetrads. Our calculations suggest that two alkali metal ions cannot take over the role of a single alkali metal ion in G4, with the possible exception of Li^+^ which, however, is unlikely to play any role under physiological conditions.

A potentially relevant scenario in which AGAG quartets might be realized is if duplex structures containing AG mismatch were to dimerize side-by-side. As evident from Fig. 3, planar or near-planar arrangements of the four purine nucleobases might be accomplished, especially with the two A’s being protonated.

## Electronic supplementary material

Below is the link to the electronic supplementary material.

**Electronic supplementary material** Schematic representations of possible AGAG quartets, Optimized geometries of quartets containing Na^+^ and K^+^, Selected geometrical properties of all investigated quartets, Cartesian coordinates of all investigated quartets and nucleobases (PDF 2515 kb)
