# Supplementary material for: Mixed guanine, adenine base quartets: possible roles of protons and metal ions in their stabilization
Source: J Biol Inorg Chem. 2017 Dec 7;23(1):41–9. doi: 10.1007/s00775-017-1507-7 (PMC5756560; doi:10.1007/s00775-017-1507-7)
Supplement: Supplementary file 1 — Electronic supplementary material Schematic representations of possible AGAG quartets, Optimized geometries of quartets containing Na+ and K+, Selected geometrical properties of all investigated quartets, Cartesian coordinates of all investigated quartets and nucleobases (PDF 2515 kb) [file 775_2017_1507_MOESM1_ESM.pdf]

Supplemental Material

Mixed guanine, adenine base quartets: Possible roles of protons and metal ions in their stabilization

Dominik A. Megger, Patrick M. Lax, Jeroen Paauwe, Célia Fonseca Guerra, Bernhard Lippert

Table of Contents:

1. Computational details

2. Figure S1

3. Figure S2

4. Figure S3

5. Table S1

6. Table S2

7. Table S3

8. Cartesian coordinates of all calculated quartets and nucleobases

Full computational details.

All calculations were performed with the Amsterdam Density Functional (ADF) program.[1] The numerical integration was carried out using the procedure developed by te Velde et al..[2] The molecular orbitals were expanded in a large uncontracted set of Slater type orbitals (STOs): TZ2P for geometry optimization (that is: triple-ξ quality, augmented by two sets of polarization functions) and QZ4P for energies (that is: quadruple-ξ quality, augmented by four sets of polarization functions).[3] An auxiliary set of s, p, d, f and g STOs were used to fit the molecular density and to represent the Coulomb and exchange potentials accurately in each self-consistent field (SCF) cycle. Equilibrium structures were obtained by optimizations using analytical gradient techniques.[4] Geometries and energies were calculated at the BLYP level of generalized gradient approximation (GGA). BLYP described the exchange by Slater's Xα potential,[5] with nonlocal corrections due to Becke[6] added self-consistently, and the gradient-corrected functional of Lee, Yang and Parr[7] is used for the correlation. Dispersion corrections are applied using the DFT-D method, developed by Grimme.[8] In this approach, the density functional is augmented with an empirical term correcting for long-range dispersion effects, described by a sum of damped interatomic potentials of the form C6/(R6+c) added to the usual DFT energy. Scalar relativistic effects were accounted for using the zeroth-order regular approximation (ZORA).[9] Solvation in water is simulated using the conductor-like screening model (COSMO).[10] Radii of cations have been computed according to the procedure presented in Ref 11 to reproduce the solvation energy of the cation.[12] (The ionic radii parameter in COSMO were set to 1.364 Å for Li^+, 1.775 for Na^+ and 2.200 for K^+).

1 (*a*) G. te Velde, F. M. Bickelhaupt, E. J. Baerends, C. Fonseca Guerra, S. J. A. van Gisbergen, J. G. Snijders and T. Ziegler, *J. Comput. Chem.*, 2001, **22**, 931-967; (*b*) C. Fonseca Guerra, J. G. Snijders, G. te Velde and E. J. Baerends, *Theor. Chem. Acc.*, 1998, **99**, 391-403. (*c*) ADF2013, SCM, Theoretical Chemistry, Vrije Universiteit, Amsterdam, The Netherlands, http://www.scm.com, E. J. Baerends, T. Ziegler, J. Autschbach, D. Bashford, A. Bérces, F. M. Bickelhaupt, C. Bo, P. M. Boerrigter, L. Cavallo, D. P. Chong, L. Deng, R. M. Dickson, D. E. Ellis, M. van Faassen, L. Fan, T. H. Fischer, C. Fonseca Guerra, A. Ghysels, A. Giammona, S. J. A. van Gisbergen, A. W. Götz, J. A. Groeneveld, O. V. Gritsenko, M. Grüning, S. Gusarov, F. E. Harris, P. van den Hoek, C. R. Jacob, H. Jacobsen, L. Jensen, J. W. Kaminski, G. van Kessel, F. Kootstra, A. Kovalenko, M. V. Krykunov, E. van Lenthe, D. A. McCormack, A. Michalak, M. Mitoraj, J. Neugebauer, V. P. Nicu, L. Noodleman, V. P. Osinga, S. Patchkovskii, P. H. T. Philipsen, D. Post, C. C. Pye, W. Ravenek, J. I. Rodríguez, P. Ros, P. R. T. Schipper, G. Schreckenbach, J. S. Seldenthuis, M. Seth, J. G. Snijders, M. Solà, M. Swart, D. Swerhone, G. te Velde, P. Vernooijs, L. Versluis, L. Visscher, O. Visser, F. Wang, T. A. Wesolowski, E. M. van Wezenbeek, G. Wiesenekker, S. K. Wolff, T. K. Woo and A. L. Yakovlev.

2 (*a*) P. M. Boerrigter, G. te Velde and E. J. Baerends, *Int. J. Quantum Chem.*, 1988, **33**, 87-113; (*b*) G. te Velde and E. J. Baerends, *J. Comput. Phys.*, 1992, **99**, 84-98.

3 E. van Lenthe and E. J. Baerends, *J. Comput. Chem.*, 2003, **24**, 1142-1156.

4 L. Versluis and T. Ziegler, *J. Chem. Phys.*, 1988, **88**, 322-328.

5 J. C. Slater, *Quantum Theory of Molecules and Solids.* McGraw-Hill: New York, 1974; Vol. 4.

6 (*a*) A. D. Becke, *J. Chem. Phys.*, 1986, **84**, 4524-4529; (*b*) A. D. Becke, *Phys. Rev. A*, 1988, **38**, 3098-3100.

7 C. Lee, W. Yang and R. G. Parr, *Phys. Rev. B*, 1988, **37**, 785-789.

8 S. Grimme, J. Anthony, S. Ehrlich, and H. Krieg, *J. Chem. Phys.* 2010, **132**, 154104.

9 (*a*) E. van Lenthe, E. J. Baerends and J. G. Snijders, *J. Chem. Phys.*, 1994, **101**, 9783-9792; (*b*) E. van Lenthe, R. van Leeuwen, E. J. Baerends and J. G. Snijders,*Int. J. Quantum Chem.*, 1996, **57**, 281-293.

10 (*a*) A. Klamt, *J. Phys. Chem.*, 1995, **99**, 2224-2235; (*b*) C. C. Pye and T. Ziegler, *Theor. Chem. Acc.*, 1999, **101**, 396-408.

11 T. van der Wijst, C. Fonseca Guerra, M. Swart, F. M. Bickelhaupt and B. Lippert, *Angew. Chem. Int. Ed.* 2009, **48**, 3285-3287

12 Y. Marcus, J. Chem. Soc., Faraday Trans., 1991, **87**, 2995–2999

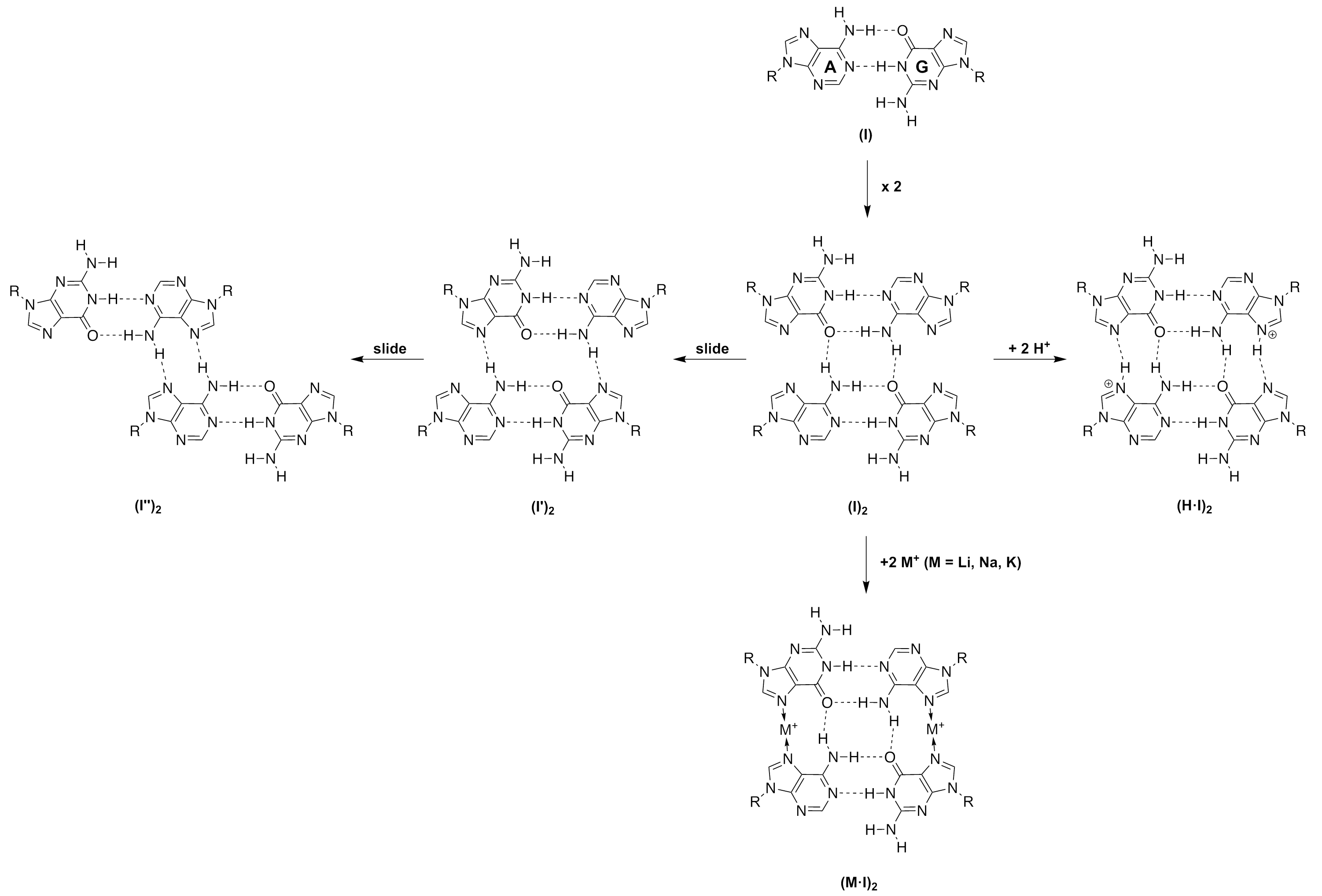

Figure S1. Schematic representation of possible AGAG quartets derived from the A · G mismatch pair (I).

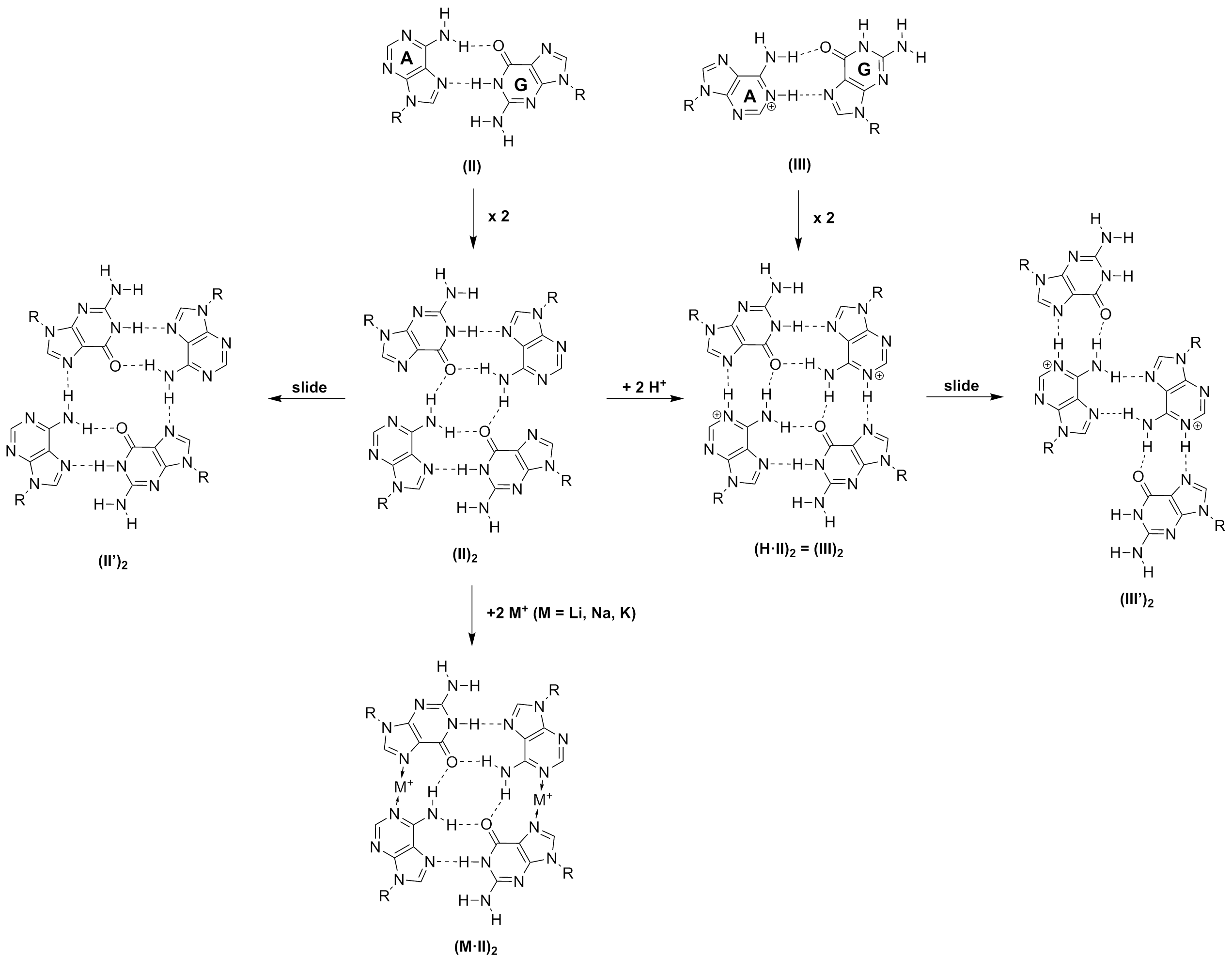

Figure S2. Schematic representation of possible AGAG quartets derived from the A · G mismatch pairs (II) and (III).

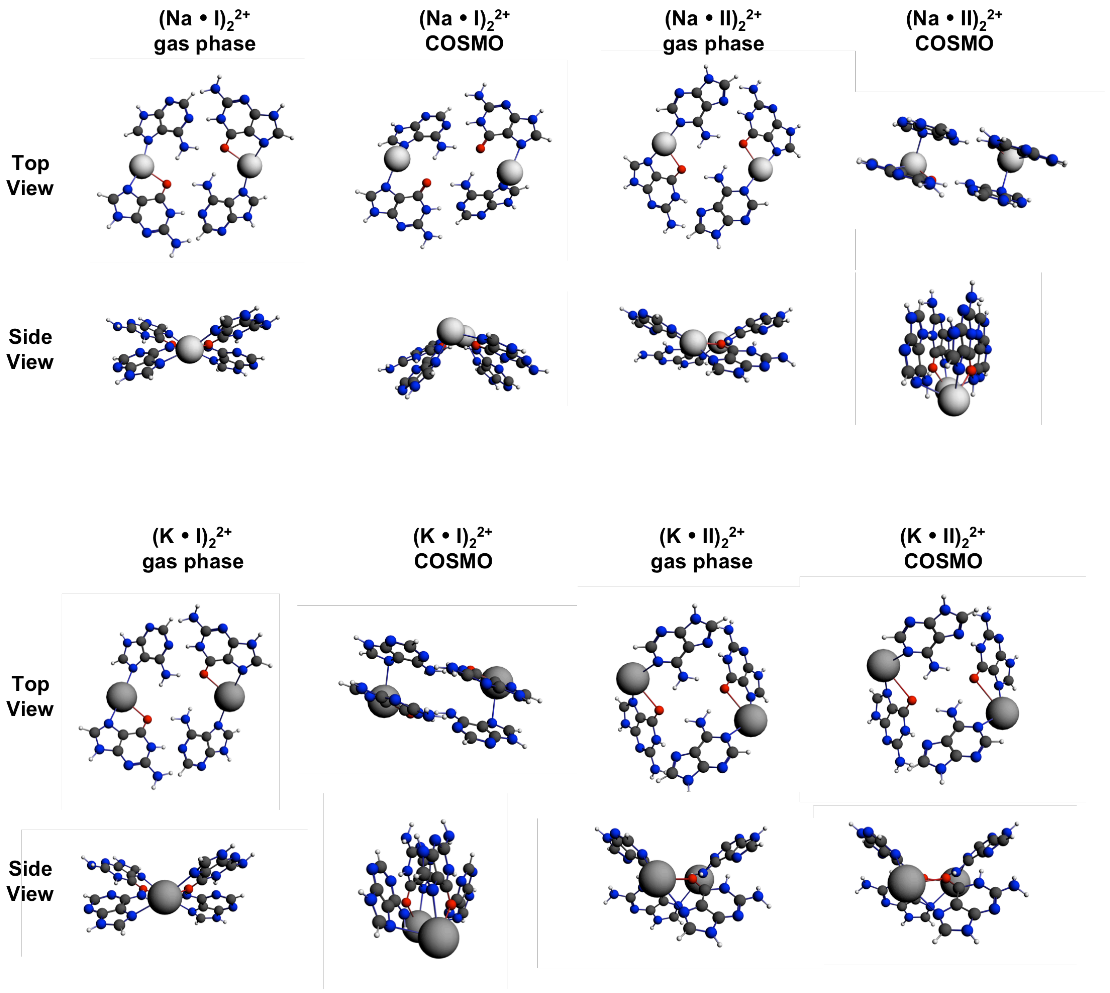

Figure S3. Geometry-optimized structures (ZORA-BLYP-D3/TZ2P) of AGAG quartets containing the alkali metal ions Na^+ and K^+.

Table S1. Selected geometrical properties[a] and bond energies[b] of neutral quartets.

Quartet	Symmetry	Environment	ΔE (kcal mol^{-1})	Hydrogen bonds					
				Distance (Å)	Angle (°)	Distance (Å)	Angle (°)	Distance (Å)	Angle (°)
				**O6(g)-N6H(a) (Hoogsteen)**	**O6(g)-N6H(a) (Hoogsteen)**	**O6(g)-N6H(a) (WC)**	**O6(g)-N6H(a) (WC)**	**N1H(g)-N1(a) (WC)**	**N1H(g)-N1(a) (WC)**
(I)$_2$	C_2	Gas phase	-45,8	2.900	144.6	2.908	178.0	2.855	176.6
	C_{2h}	Gas phase	-42,6	2.949	148.9	2.922	179.5	2.917	176.1
	C_2	Water	-15,2	2.945	147.9	2.920	178.0	2.874	175.6
	C_{2h}	Water	-9,3	3.008	154.7	2.918	179.9	2.937	177.7
				**N7(g)-N6H(a) (Hoogsteen)**	**N7(g)-N6H(a) (Hoogsteen)**	**O6(g)-N6H(a) (WC)**	**O6(g)-N6H(a) (WC)**	**N1H(g)-N1(a) (WC)**	**N1H(g)-N1(a) (WC)**
(I')$_2$	C_2	Gas phase	-54,0	2.913	167.8	2.920	172.9	2.860	173.2
	C_{2h}	Gas phase	-52,2	2.951	170.6	2.870	175.0	2.921	178.4
	C_2	Water	-18,7	2.944	169.0	2.957	174.6	2.873	175.0
	C_{2h}	Water	-16,5	2.977	171.7	2.876	176.2	2.951	177.4
				**N7(a)-N6H(a) (Hoogsteen)**	**N7(a)-N6H(a) (Hoogsteen)**	**O6(g)-N6H(a) (WC)**	**O6(g)-N6H(a) (WC)**	**N1H(g)-N1(a) (WC)**	**N1H(g)-N1(a) (WC)**
(I'')$_2$	C_2	Gas phase	-51,9	2.934	149.3	2.892	159.7	2.891	170.8
	C_{2h}	Gas phase	-51,3	2.906	155.7	2.816	173.7	2.970	176.0
	C_2	Water	-18,5	2.961	159.2	2.889	171.1	2.897	171.0
	C_{2h}	Water	-17,5	2.953	159.4	2.855	177.1	2.955	178.8
				**O6(g)-N6H(a) (Hoogsteen)**	**O6(g)-N6H(a) (Hoogsteen)**	**O6(g)-N6H(a) (WC)**	**O6(g)-N6H(a) (WC)**	**N1H(g)-N7(a) (Hoogsteen)**	**N1H(g)-N7(a) (Hoogsteen)**
(II)$_2$	C_2	Gas phase	n.d.[c]	n.d.	n.d.	n.d.	n.d.	n.d.	n.d.
	C_{2h}	Gas phase	n.d.[c]	n.d.	n.d.	n.d.	n.d.	n.d.	n.d.
	C_2	Water	-14,4	2.897	163.9	2.903	149.4	2.883	172.6
	C_{2h}	Water	-7,3	2.907	165.0	3.010	166.3	2.960	173.2
				**O6(g)-N6H(a) (Hoogsteen)**	**O6(g)-N6H(a) (Hoogsteen)**	**N7(g)-N6H(a) (WC)**	**N7(g)-N6H(a) (WC)**	**N1H(g)-N7(a) (Hoogsteen)**	**N1H(g)-N7(a) (Hoogsteen)**
(II')$_2$	C_2	Gas phase	-50,1	2.876	165.1	2.924	165.6	2.898	171.0
	C_{2h}	Gas phase	-46,3	2.827	165.0	2.916	177.7	2.992	176.4
	C_2	Water	-17,6	2.942	166.2	3.010	155.7	2.878	175.1
	C_{2h}	Water	-13,7	2.847	163.7	2.933	178.1	2.980	177.3

a) Geometries were optimized at ZORA-BLYP-D3/TZ2P level of theory.
b) Energies were calculated at ZORA-BLYP-D3/QZ4P level of theory.
c) In the gas phase, the quartet (II)$_2$ collapsed into (II')$_2$. Hence, no bond energies and geometrical properties could be assessed.

Table S2. Selected geometrical properties[a] and bond energies[b] of protonated quartets.

Quartet	Symmetry	Environment	ΔE (kcal mol^{-1})	Hydrogen bonds							
				Distance (Å)	Angle (°)	Distance (Å)	Angle (°)	Distance (Å)	Angle (°)	Distance (Å)	Angle (°)
				N1H(g)-N1(a) (WC)	N1H(g)-N1(a) (WC)	O6(g)-N6H(a) (WC)	O6(g)-N6H(a) (WC)	O6(g)-N6H(a) (Hoogsteen)	O6(g)-N6H(a) (Hoogsteen)	N7(g)-N7H(a) (Hoogsteen)	N7(g)-N7H(a) (Hoogsteen)
$(H \bullet I)_2$	C_2	Gas phase	-73.6	3.019	170.7	2.799	169.7	2.736	152.7	2.818	166.9
	C_{2h}	Gas phase	-73.9	3.098	177.0	2.778	173.6	2.728	152.3	2.821	167.5
	C_2	Water	-30.3	2.931	168.6	2.868	174.9	2.836	153.0	2.784	168.2
	C_{2h}	Water	-28.6	3.069	177.3	2.772	174.0	2.750	148.2	2.855	168.4
				N1H(g)-N7(a) (Hoogsteen)	N1H(g)-N7(a) (Hoogsteen)	O6(g)-N6H(a) (WC)	O6(g)-N6H(a) (WC)	O6(g)-N6H(a) (Hoogsteen)	O6(g)-N6H(a) (Hoogsteen)	N7(g)-N1H(a) (WC)	N7(g)-N1H(a) (WC)
$(H \bullet II)_2$ / $(III)_2$	C_2	Gas phase	-73.9	2.988	171.0	2.747	162.8	2.768	160.3	2.863	175.9
	C_{2h}	Gas phase	-73.2	3.088	178.6	2.768	165.0	2.760	160.2	2.828	174.4
	C_2	Water	-30.3	2.962	171.0	2.802	164.0	2.796	162.0	2.819	173.5
	C_{2h}	Water	-29.0	3.026	179.3	2.771	162.4	2.763	160.9	2.847	177.0
				O6(g)-N6H(a) (WC)	O6(g)-N6H(a) (WC)	N7(a)-N6H(a) (Hoogsteen)	N7(a)-N6H(a) (Hoogsteen)	N7(g)-N1H(a) (WC)	N7(g)-N1H(a) (WC)		
$(III')_2$	C_2	Gas phase	-68.0	2.790	164.6	2.889	159.6	2.797	175.2		
	C_{2h}	Gas phase	-68.0	2.783	164.6	2.889	159.5	2.796	175.3		
	C_2	Water	-23.6	2.810	162.0	2.887	158.9	2.800	176.8		
	C_{2h}	Water	-23.4	2.804	162.0	2.887	158.9	2.801	176.9		

a) Geometries were optimized at ZORA-BLYP-D3/TZ2P level of theory.
b) Energies were calculated at ZORA-BLYP-D3/QZ4P level of theory.

Table S3. Selected geometrical properties[a] and bond energies[b] of metalated quartets.

Quartet	Symmetry	Environment	ΔE (kcal mol^{-1})	Coordinative bonds				Hydrogen bonds					
				Distance (Å)	Distance (Å)	Distance (Å)	Angle (°)	Distance (Å)	Angle (°)	Distance (Å)	Angle (°)	Distance (Å)	Angle (°)
				N7(g)-Metal	N7(a)-Metal	O6(g)-Metal	N7(g)-Metal-N7(a)	N1H(g)-N1(a) (WC)	N1H(g)-N1(a) (WC)	O6(g)-N6H(a) (WC)	O6(g)-N6H(a) (WC)	O6(g)-N6H(a) (Hoogsteen)	O6(g)-N6H(a) (Hoogsteen)
(Li • I)$_2$	C_2	Gas phase	-209.3	2.037	1.979	2.041	141.3	2.829	175.0	3.044	173.9	3.027	175.2
	C_{2h}	Gas phase	-207.9	2.021	1.965	2.023	135.7	2.910	178.7	2.954	178.2	3.280	176.2
	C_2	Water	-120.7	2.120	2.055	2.202	98.8	2.882	177.0	2.988	176.8	3.133	152.6
	C_{2h}	Water	-114.8	2.047	1.984	2.076	135.5	2.909	179.3	2.921	178.8	3.341	173.7
(Na • I)$_2$	C_2	Gas phase	-161.1	2.354	2.359	2.422	163.0	2.811	175.6	3.138	171.3	3.184	162.1
	C_{2h}	Gas phase	-157.3	2.361	2.366	2.374	147.1	2.914	179.2	2.902	179.7	4.088	172.0
	C_2	Water	-126.3	2.390	2.419	2.947	103.7	2.861	175.0	2.989	176.9	3.003	165.7
	C_{2h}	Water	-118.5	2.431	2.393	2.447	148.3	2.971	179.9	2.874	179.7	4.255	170.9
(K • I)$_2$	C_2	Gas phase	-119.8	2.762	2.836	2.777	170.1	2.822	175.2	3.019	172.4	3.854	134.6
	C_{2h}	Gas phase	-117.0	2.765	2.877	2.752	156.4	2.931	179.2	2.866	179.4	5.130	169.0
	C_2	Water	-125.6	2.099	2.999	2.857	73.0	2.843	174.2	2.884	174.3	3.347	73.7
	C_{2h}	Water	-112.5	2.883	2.887	2.880	157.0	2.932	179.6	2.849	179.6	5.307	168.1
				N7(g)-Metal	N1(a)-Metal	O6(g)-Metal	N7(g)-Metal-N1(a)	N1H(g)-N7(a) (Hoogsteen)	N1H(g)-N7(a) (Hoogsteen)	O6(g)-N6H(a) (WC)	O6(g)-N6H(a) (WC)	O6(g)-N6H(a) (Hoogsteen)	O6(g)-N6H(a) (Hoogsteen)
(Li • II)$_2$	C_2	Gas phase	-207.3	2.030	1.990	2.079	147.0	2.849	170.8	3.092	165.0	3.002	162.9
	C_{2h}	Gas phase	-204.1	2.013	1.975	2.074	143.5	2.990	172.3	3.256	165.3	2.926	159.1
	C_2	Water	-121.3	1.999	2.030	3.119	112.2	2.844	176.1	2.926	165.1	2.959	167.0
	C_{2h}	Water	-112.6	2.038	2.003	2.105	143.5	2.967	173.0	3.294	166.9	2.919	160.0
(Na • II)$_2$	C_2	Gas phase	-159.9	2.350	2.371	2.455	172.9	2.837	170.8	3.333	172.6	3.062	166.1
	C_{2h}	Gas phase	-155.6	2.354	2.376	2.398	154.6	2.969	173.7	4.088	166.7	2.863	163.0
	C_2	Water	-130.3	2.491	2.640	2.487	88.0	2.897	176.7	3.352	64.9	2.841	165.5
	C_{2h}	Water	-117.6	2.408	2.411	2.451	154.4	2.950	175.1	4.201	167.4	2.853	163.5
(K • II)$_2$	C_2	Gas phase	-121.8	2.776	2.814	3.003	141.4	2.851	172.7	3.046	178.4	3.187	169.1
	C_{2h}	Gas phase	-116.3	2.765	2.853	2.779	166.1	2.984	174.0	5.073	165.6	2.834	162.6
	C_2	Water	-120.2	2.829	2.870	3.311	132.7	2.846	173.7	2.965	169.6	3.053	168.3
	C_{2h}	Water	-112.3	2.854	2.904	2.872	163.4	2.953	176.3	5.245	167.2	2.830	163.9

a) Geometries were optimized at ZORA-BLYP-D3/TZ2P level of theory.
b) Energies were calculated at ZORA-BLYP-D3/QZ4P level of theory.

(I)$_2$ gas phase C$_2$ E = -9557.8787

N	-0.501700	-3.785600	0.129000
C	-0.404100	-5.106900	0.397500
N	0.692900	-5.839000	0.622500
C	1.802600	-5.073400	0.560600
C	1.869100	-3.698300	0.284700
C	0.639600	-3.033600	0.028200
N	3.117500	-5.458000	0.744900
C	3.893500	-4.316300	0.577700
N	3.182000	-3.244000	0.301900
N	0.548400	-1.738200	-0.298300
N	-3.041900	-2.492900	-0.037000
C	-4.123700	-3.204500	0.414600
N	-5.383400	-2.832000	0.254900
C	-5.490100	-1.661700	-0.421000
C	-4.466400	-0.825000	-0.897100
C	-3.108400	-1.231900	-0.684600
N	-6.659300	-1.025000	-0.771100
C	-6.294500	0.144800	-1.437400
N	-4.993500	0.295000	-1.528200
O	-2.054600	-0.641500	-0.990300
N	-3.848400	-4.407900	1.040800
N	0.501700	3.785600	0.129000
C	0.404100	5.106900	0.397500
N	-0.692900	5.839000	0.622500
C	-1.802600	5.073400	0.560600
C	-1.869100	3.698300	0.284700
C	-0.639600	3.033600	0.028200
N	-3.117500	5.458000	0.744900
C	-3.893500	4.316300	0.577700
N	-3.182000	3.244000	0.301900
N	-0.548400	1.738200	-0.298300
N	3.041900	2.492900	-0.037000
C	4.123700	3.204500	0.414600
N	5.383400	2.832000	0.254900
C	5.490100	1.661700	-0.421000
C	4.466400	0.825000	-0.897100
C	3.108400	1.231900	-0.684600
N	6.659300	1.025000	-0.771100
C	6.294500	-0.144800	-1.437400
N	4.993500	-0.295000	-1.528200
O	2.054600	0.641500	-0.990300
N	3.848400	4.407900	1.040800
H	-1.350100	-5.646000	0.418000
H	3.434400	-6.397100	0.951900
H	4.970700	-4.346800	0.674900
H	1.382200	-1.180300	-0.479200
H	-0.360800	-1.342700	-0.562100
H	-2.086700	-2.935400	0.031800
H	-7.593500	-1.364800	-0.578400

H	-7.038900	0.826500	-1.826400
H	-2.972800	-4.467700	1.548900
H	-4.646500	-4.803100	1.525500
H	1.350100	5.646000	0.418000
H	-3.434400	6.397100	0.951900
H	-4.970700	4.346800	0.674900
H	-1.382200	1.180300	-0.479200
H	0.360800	1.342700	-0.562100
H	2.086700	2.935400	0.031800
H	7.593500	1.364800	-0.578400
H	7.038900	-0.826500	-1.826400
H	2.972800	4.467700	1.548900
H	4.646500	4.803100	1.525500

(I)$_2$ gas phase C_{2h} E = -9554.6754

N	-0.512200	-3.973800	0.000000
C	-0.367400	-5.317000	0.000000
N	0.745500	-6.056100	0.000000
C	1.840200	-5.266400	0.000000
C	1.864600	-3.863900	0.000000
C	0.613400	-3.189000	0.000000
N	3.169800	-5.641800	0.000000
C	3.913200	-4.464500	0.000000
N	3.165400	-3.383300	0.000000
N	0.502700	-1.854100	0.000000
N	-3.127300	-2.681600	0.000000
C	-4.213400	-3.524400	0.000000
N	-5.474700	-3.117700	0.000000
C	-5.577500	-1.769100	0.000000
C	-4.554300	-0.803900	0.000000
C	-3.194100	-1.260500	0.000000
N	-6.748800	-1.046400	0.000000
C	-6.387600	0.301100	0.000000
N	-5.088200	0.480100	0.000000
O	-2.136600	-0.600600	0.000000
N	-3.958800	-4.866900	0.000000
N	0.512200	3.973800	0.000000
C	0.367400	5.317000	0.000000
N	-0.745500	6.056100	0.000000
C	-1.840200	5.266400	0.000000
C	-1.864600	3.863900	0.000000
C	-0.613400	3.189000	0.000000
N	-3.169800	5.641800	0.000000
C	-3.913200	4.464500	0.000000
N	-3.165400	3.383300	0.000000
N	-0.502700	1.854100	0.000000
N	3.127300	2.681600	0.000000
C	4.213400	3.524400	0.000000
N	5.474700	3.117700	0.000000

C	5.577500	1.769100	0.000000
C	4.554300	0.803900	0.000000
C	3.194100	1.260500	0.000000
N	6.748800	1.046400	0.000000
C	6.387600	-0.301100	0.000000
N	5.088200	-0.480100	0.000000
O	2.136600	0.600600	0.000000
N	3.958800	4.866900	0.000000
H	-1.292600	-5.894300	0.000000
H	3.515200	-6.593500	0.000000
H	4.995200	-4.479400	0.000000
H	1.333200	-1.260700	0.000000
H	-0.421200	-1.409200	0.000000
H	-2.168000	-3.105100	0.000000
H	-7.681100	-1.441700	0.000000
H	-7.135400	1.083000	0.000000
H	-3.020300	-5.231100	0.000000
H	-4.744900	-5.499300	0.000000
H	1.292600	5.894300	0.000000
H	-3.515200	6.593500	0.000000
H	-4.995200	4.479400	0.000000
H	-1.333200	1.260700	0.000000
H	0.421200	1.409200	0.000000
H	2.168000	3.105100	0.000000
H	7.681100	1.441700	0.000000
H	7.135400	-1.083000	0.000000
H	3.020300	5.231100	0.000000
H	4.744900	5.499300	0.000000

(I)$_2$ water C$_2$ E = -9607.7009

N	-0.502400	-3.754500	0.130500
C	-0.397600	-5.083100	0.346200
N	0.714500	-5.815400	0.508400
C	1.826100	-5.046700	0.441600
C	1.870200	-3.659500	0.220100
C	0.631600	-2.996400	0.039800
N	3.143900	-5.428300	0.564600
C	3.908800	-4.289500	0.420900
N	3.185100	-3.200400	0.209800
N	0.515200	-1.676600	-0.202500
N	-3.064500	-2.456200	0.040300
C	-4.139800	-3.162200	0.528200
N	-5.405700	-2.785700	0.364600
C	-5.522900	-1.638200	-0.347000
C	-4.495500	-0.833000	-0.869200
C	-3.146300	-1.242900	-0.665400
N	-6.687900	-1.005900	-0.706600
C	-6.334800	0.123800	-1.423400
N	-5.027000	0.265300	-1.542800

O	-2.092600	-0.664700	-1.040900
N	-3.856900	-4.340100	1.168900
N	0.502400	3.754500	0.130500
C	0.397600	5.083100	0.346200
N	-0.714500	5.815400	0.508400
C	-1.826100	5.046700	0.441600
C	-1.870200	3.659500	0.220100
C	-0.631600	2.996400	0.039800
N	-3.143900	5.428300	0.564600
C	-3.908800	4.289500	0.420900
N	-3.185100	3.200400	0.209800
N	-0.515200	1.676600	-0.202500
N	3.064500	2.456200	0.040300
C	4.139800	3.162200	0.528200
N	5.405700	2.785700	0.364600
C	5.522900	1.638200	-0.347000
C	4.495500	0.833000	-0.869200
C	3.146300	1.242900	-0.665400
N	6.687900	1.005900	-0.706600
C	6.334800	-0.123800	-1.423400
N	5.027000	-0.265300	-1.542800
O	2.092600	0.664700	-1.040900
N	3.856900	4.340100	1.168900
H	-1.339500	-5.625700	0.384000
H	3.488000	-6.367300	0.731700
H	4.987400	-4.324400	0.484400
H	1.338400	-1.113200	-0.409100
H	-0.399700	-1.304400	-0.480500
H	-2.113200	-2.895800	0.108300
H	-7.629800	-1.315800	-0.494000
H	-7.082700	0.790900	-1.827500
H	-2.960000	-4.418900	1.635000
H	-4.630500	-4.753400	1.678800
H	1.339500	5.625700	0.384000
H	-3.488000	6.367300	0.731700
H	-4.987400	4.324400	0.484400
H	-1.338400	1.113200	-0.409100
H	0.399700	1.304400	-0.480500
H	2.113200	2.895800	0.108300
H	7.629800	1.315800	-0.494000
H	7.082700	-0.790900	-1.827500
H	2.960000	4.418900	1.635000
H	4.630500	4.753400	1.678800

(I)$_2$ water C_{2h} E = -9601.7804

N	-0.542000	-3.937600	0.000000
C	-0.387600	-5.279000	0.000000
N	0.742800	-5.999400	0.000000
C	1.833600	-5.198700	0.000000

C	1.832100	-3.795100	0.000000
C	0.573100	-3.143500	0.000000
N	3.164800	-5.553200	0.000000
C	3.892500	-4.380200	0.000000
N	3.130700	-3.297600	0.000000
N	0.436200	-1.805200	0.000000
N	-3.207400	-2.702900	0.000000
C	-4.291000	-3.556700	0.000000
N	-5.558100	-3.140600	0.000000
C	-5.666400	-1.792500	0.000000
C	-4.635700	-0.835100	0.000000
C	-3.284800	-1.294600	0.000000
N	-6.830800	-1.064100	0.000000
C	-6.474300	0.273600	0.000000
N	-5.167000	0.455500	0.000000
O	-2.226500	-0.612500	0.000000
N	-4.034900	-4.886600	0.000000
N	0.542000	3.937600	0.000000
C	0.387600	5.279000	0.000000
N	-0.742800	5.999400	0.000000
C	-1.833600	5.198700	0.000000
C	-1.832100	3.795100	0.000000
C	-0.573100	3.143500	0.000000
N	-3.164800	5.553200	0.000000
C	-3.892500	4.380200	0.000000
N	-3.130700	3.297600	0.000000
N	-0.436200	1.805200	0.000000
N	3.207400	2.702900	0.000000
C	4.291000	3.556700	0.000000
N	5.558100	3.140600	0.000000
C	5.666400	1.792500	0.000000
C	4.635700	0.835100	0.000000
C	3.284800	1.294600	0.000000
N	6.830800	1.064100	0.000000
C	6.474300	-0.273600	0.000000
N	5.167000	-0.455500	0.000000
O	2.226500	0.612500	0.000000
N	4.034900	4.886600	0.000000
H	-1.304300	-5.863700	0.000000
H	3.539400	-6.495500	0.000000
H	4.973400	-4.387900	0.000000
H	1.255300	-1.199600	0.000000
H	-0.499400	-1.384800	0.000000
H	-2.247600	-3.118300	0.000000
H	-7.772700	-1.439900	0.000000
H	-7.221800	1.054300	0.000000
H	-3.096100	-5.251800	0.000000
H	-4.807600	-5.538100	0.000000
H	1.304300	5.863700	0.000000
H	-3.539400	6.495500	0.000000

H	-4.973400	4.387900	0.000000
H	-1.255300	1.199600	0.000000
H	0.499400	1.384800	0.000000
H	2.247600	3.118300	0.000000
H	7.772700	1.439900	0.000000
H	7.221800	-1.054300	0.000000
H	3.096100	5.251800	0.000000
H	4.807600	5.538100	0.000000

(I')$_2$ gas phase C$_2$ E = -9566.0925

N	3.637200	-2.313700	-0.585900
C	4.731800	-3.083200	-0.414600
N	5.998900	-2.709300	-0.199300
C	6.098400	-1.363400	-0.161700
C	5.067800	-0.425100	-0.331000
C	3.760200	-0.944200	-0.582400
N	7.238000	-0.607800	0.046100
C	6.849900	0.722200	-0.004800
N	5.559800	0.876200	-0.228000
N	2.691500	-0.182000	-0.812900
N	1.018800	-3.370200	-0.132500
C	0.892800	-4.506700	0.625600
N	-0.263300	-5.051000	0.981300
C	-1.326500	-4.341800	0.526200
C	-1.321600	-3.160800	-0.222100
C	-0.067000	-2.576900	-0.600300
N	-2.664000	-4.607400	0.754300
C	-3.392900	-3.582800	0.159000
N	-2.613900	-2.705500	-0.434600
O	0.136000	-1.531600	-1.230100
N	2.070100	-5.130100	0.998700
N	-3.637200	2.313700	-0.585900
C	-4.731800	3.083200	-0.414600
N	-5.998900	2.709300	-0.199300
C	-6.098400	1.363400	-0.161700
C	-5.067800	0.425100	-0.331000
C	-3.760200	0.944200	-0.582400
N	-7.238000	0.607800	0.046100
C	-6.849900	-0.722200	-0.004800
N	-5.559800	-0.876200	-0.228000
N	-2.691500	0.182000	-0.812900
N	-1.018800	3.370200	-0.132500
C	-0.892800	4.506700	0.625600
N	0.263300	5.051000	0.981300
C	1.326500	4.341800	0.526200
C	1.321600	3.160800	-0.222100
C	0.067000	2.576900	-0.600300
N	2.664000	4.607400	0.754300
C	3.392900	3.582800	0.159000

N	2.613900	2.705500	-0.434600
O	-0.136000	1.531600	-1.230100
N	-2.070100	5.130100	0.998700
H	4.554800	-4.157700	-0.467500
H	8.169400	-0.975900	0.193800
H	7.564200	1.523800	0.129500
H	2.766200	0.844400	-0.777300
H	1.777500	-0.602600	-1.019800
H	1.978800	-3.010200	-0.372500
H	-3.025700	-5.393500	1.280100
H	-4.470400	-3.516700	0.210000
H	2.885400	-4.537900	1.114800
H	1.947800	-5.830800	1.721100
H	-4.554800	4.157700	-0.467500
H	-8.169400	0.975900	0.193800
H	-7.564200	-1.523800	0.129500
H	-2.766200	-0.844400	-0.777300
H	-1.777500	0.602600	-1.019800
H	-1.978800	3.010200	-0.372500
H	3.025700	5.393500	1.280100
H	4.470400	3.516700	0.210000
H	-2.885400	4.537900	1.114800
H	-1.947800	5.830800	1.721100

(I')$_2$ gas phase C_{2h} E = -9564.2519

N	3.701000	-2.169100	0.000000
C	4.837000	-2.893700	0.000000
N	6.110600	-2.485900	0.000000
C	6.175200	-1.136800	0.000000
C	5.101100	-0.235500	0.000000
C	3.784900	-0.793900	0.000000
N	7.308000	-0.343600	0.000000
C	6.871700	0.973100	0.000000
N	5.558100	1.082400	0.000000
N	2.678500	-0.053800	0.000000
N	1.115100	-3.528300	0.000000
C	1.019500	-4.899900	0.000000
N	-0.123500	-5.577800	0.000000
C	-1.207400	-4.763700	0.000000
C	-1.235100	-3.367200	0.000000
C	-0.000200	-2.643300	0.000000
N	-2.538400	-5.139100	0.000000
C	-3.294600	-3.968800	0.000000
N	-2.538400	-2.893800	0.000000
O	0.159100	-1.417700	0.000000
N	2.194500	-5.600400	0.000000
N	-3.701000	2.169100	0.000000
C	-4.837000	2.893700	0.000000
N	-6.110600	2.485900	0.000000

C	-6.175200	1.136800	0.000000
C	-5.101100	0.235500	0.000000
C	-3.784900	0.793900	0.000000
N	-7.308000	0.343600	0.000000
C	-6.871700	-0.973100	0.000000
N	-5.558100	-1.082400	0.000000
N	-2.678500	0.053800	0.000000
N	-1.115100	3.528300	0.000000
C	-1.019500	4.899900	0.000000
N	0.123500	5.577800	0.000000
C	1.207400	4.763700	0.000000
C	1.235100	3.367200	0.000000
C	0.000200	2.643300	0.000000
N	2.538400	5.139100	0.000000
C	3.294600	3.968800	0.000000
N	2.538400	2.893800	0.000000
O	-0.159100	1.417700	0.000000
N	-2.194500	5.600400	0.000000
H	4.704100	-3.977100	0.000000
H	8.263000	-0.679600	0.000000
H	7.571200	1.798600	0.000000
H	2.739300	0.973200	0.000000
H	1.748600	-0.491700	0.000000
H	2.054100	-3.055800	0.000000
H	-2.881500	-6.091700	0.000000
H	-4.375500	-3.967600	0.000000
H	3.089800	-5.138300	0.000000
H	2.149200	-6.608300	0.000000
H	-4.704100	3.977100	0.000000
H	-8.263000	0.679600	0.000000
H	-7.571200	-1.798600	0.000000
H	-2.739300	-0.973200	0.000000
H	-1.748600	0.491700	0.000000
H	-2.054100	3.055800	0.000000
H	2.881500	6.091700	0.000000
H	4.375500	3.967600	0.000000
H	-3.089800	5.138300	0.000000
H	-2.149200	6.608300	0.000000

(I')$_2$ water C$_2$ E = -9611.1227

N	3.645100	-2.312300	-0.654500
C	4.721200	-3.080600	-0.385900
N	5.980600	-2.693500	-0.139100
C	6.100100	-1.346100	-0.188100
C	5.078500	-0.423100	-0.467100
C	3.782900	-0.950800	-0.715700
N	7.228100	-0.582000	0.012300
C	6.857500	0.735400	-0.152700
N	5.573700	0.880400	-0.443900

N	2.714500	-0.193800	-1.011400
N	1.003900	-3.269600	-0.052400
C	0.870900	-4.309800	0.837400
N	-0.300800	-4.815800	1.215500
C	-1.354000	-4.187100	0.638000
C	-1.324600	-3.115400	-0.263600
C	-0.064100	-2.579500	-0.659700
N	-2.691200	-4.440800	0.843600
C	-3.405000	-3.529300	0.089900
N	-2.616400	-2.718400	-0.593200
O	0.157700	-1.618900	-1.432400
N	2.032800	-4.868000	1.307400
N	-3.645100	2.312300	-0.654500
C	-4.721200	3.080600	-0.385900
N	-5.980600	2.693500	-0.139100
C	-6.100100	1.346100	-0.188100
C	-5.078500	0.423100	-0.467100
C	-3.782900	0.950800	-0.715700
N	-7.228100	0.582000	0.012300
C	-6.857500	-0.735400	-0.152700
N	-5.573700	-0.880400	-0.443900
N	-2.714500	0.193800	-1.011400
N	-1.003900	3.269600	-0.052400
C	-0.870900	4.309800	0.837400
N	0.300800	4.815800	1.215500
C	1.354000	4.187100	0.638000
C	1.324600	3.115400	-0.263600
C	0.064100	2.579500	-0.659700
N	2.691200	4.440800	0.843600
C	3.405000	3.529300	0.089900
N	2.616400	2.718400	-0.593200
O	-0.157700	1.618900	-1.432400
N	-2.032800	4.868000	1.307400
H	4.535900	-4.153400	-0.370300
H	8.156700	-0.924100	0.234700
H	7.573300	1.539300	-0.048700
H	2.775200	0.832300	-0.960300
H	1.800600	-0.631800	-1.169000
H	1.966800	-2.950800	-0.321000
H	-3.079900	-5.153600	1.451700
H	-4.484500	-3.492200	0.093900
H	2.851300	-4.270300	1.355600
H	1.919900	-5.476500	2.112000
H	-4.535900	4.153400	-0.370300
H	-8.156700	0.924100	0.234700
H	-7.573300	-1.539300	-0.048700
H	-2.775200	-0.832300	-0.960300
H	-1.800600	0.631800	-1.169000
H	-1.966800	2.950800	-0.321000
H	3.079900	5.153600	1.451700

H	4.484500	3.492200	0.093900
H	-2.851300	4.270300	1.355600
H	-1.919900	5.476500	2.112000

(I')$_2$ water C_{2h} E = -9608.9406

N	3.706800	-2.168500	0.000000
C	4.840400	-2.898300	0.000000
N	6.113700	-2.477900	0.000000
C	6.183900	-1.125800	0.000000
C	5.100300	-0.234700	0.000000
C	3.795100	-0.798700	0.000000
N	7.306000	-0.326600	0.000000
C	6.868800	0.981000	0.000000
N	5.548400	1.086600	0.000000
N	2.676800	-0.060000	0.000000
N	1.104500	-3.559100	0.000000
C	1.017000	-4.936000	0.000000
N	-0.138600	-5.606200	0.000000
C	-1.221600	-4.792700	0.000000
C	-1.238000	-3.393600	0.000000
C	-0.002800	-2.686900	0.000000
N	-2.549000	-5.160300	0.000000
C	-3.299500	-3.997400	0.000000
N	-2.543900	-2.914300	0.000000
O	0.157000	-1.446100	0.000000
N	2.180900	-5.629200	0.000000
N	-3.706800	2.168500	0.000000
C	-4.840400	2.898300	0.000000
N	-6.113700	2.477900	0.000000
C	-6.183900	1.125800	0.000000
C	-5.100300	0.234700	0.000000
C	-3.795100	0.798700	0.000000
N	-7.306000	0.326600	0.000000
C	-6.868800	-0.981000	0.000000
N	-5.548400	-1.086600	0.000000
N	-2.676800	0.060000	0.000000
N	-1.104500	3.559100	0.000000
C	-1.017000	4.936000	0.000000
N	0.138600	5.606200	0.000000
C	1.221600	4.792700	0.000000
C	1.238000	3.393600	0.000000
C	0.002800	2.686900	0.000000
N	2.549000	5.160300	0.000000
C	3.299500	3.997400	0.000000
N	2.543900	2.914300	0.000000
O	-0.157000	1.446100	0.000000
N	-2.180900	5.629200	0.000000
H	4.707400	-3.978600	0.000000
H	8.270500	-0.639900	0.000000

H	7.565800	1.807900	0.000000
H	2.728800	0.968000	0.000000
H	1.756300	-0.516600	0.000000
H	2.041700	-3.092600	0.000000
H	-2.910500	-6.108000	0.000000
H	-4.380000	-4.008200	0.000000
H	3.080000	-5.171500	0.000000
H	2.152900	-6.639800	0.000000
H	-4.707400	3.978600	0.000000
H	-8.270500	0.639900	0.000000
H	-7.565800	-1.807900	0.000000
H	-2.728800	-0.968000	0.000000
H	-1.756300	0.516600	0.000000
H	-2.041700	3.092600	0.000000
H	2.910500	6.108000	0.000000
H	4.380000	4.008200	0.000000
H	-3.080000	5.171500	0.000000
H	-2.152900	6.639800	0.000000

(I'')$_2$ gas phase C$_2$ E = -9563.9575

N	3.133500	2.329100	0.643500
C	2.927800	3.506000	0.006600
N	1.777700	4.156300	-0.205800
C	0.733400	3.480200	0.313900
C	0.786200	2.265400	1.012600
C	2.060000	1.659600	1.164900
N	-0.614100	3.790200	0.245800
C	-1.300700	2.769100	0.879900
N	-0.491300	1.841500	1.360500
N	2.240100	0.476000	1.784000
N	-5.407900	-0.668700	-0.011000
C	-6.455600	-1.320000	-0.613100
N	-7.447800	-0.715200	-1.245800
C	-7.307700	0.633600	-1.248100
C	-6.308000	1.414600	-0.646300
C	-5.253000	0.746300	0.061300
N	-8.156800	1.541500	-1.841000
C	-7.639400	2.807800	-1.578000
N	-6.536300	2.768600	-0.864500
O	-4.293600	1.235300	0.680400
N	-6.420700	-2.701400	-0.570300
N	-3.133500	-2.329100	0.643500
C	-2.927800	-3.506000	0.006600
N	-1.777700	-4.156300	-0.205800
C	-0.733400	-3.480200	0.313900
C	-0.786200	-2.265400	1.012600
C	-2.060000	-1.659600	1.164900
N	0.614100	-3.790200	0.245800
C	1.300700	-2.769100	0.879900

N	0.491300	-1.841500	1.360500
N	-2.240100	-0.476000	1.784000
N	5.407900	0.668700	-0.011000
C	6.455600	1.320000	-0.613100
N	7.447800	0.715200	-1.245800
C	7.307700	-0.633600	-1.248100
C	6.308000	-1.414600	-0.646300
C	5.253000	-0.746300	0.061300
N	8.156800	-1.541500	-1.841000
C	7.639400	-2.807800	-1.578000
N	6.536300	-2.768600	-0.864500
O	4.293600	-1.235300	0.680400
N	6.420700	2.701400	-0.570300
H	3.819300	3.973900	-0.407300
H	-1.015300	4.594600	-0.220900
H	-2.382800	2.739400	0.927900
H	1.423400	-0.140900	1.865800
H	3.099800	-0.040900	1.567100
H	-4.605700	-1.252700	0.329300
H	7.291100	3.143400	-0.843700
H	-8.126200	3.702700	-1.942000
H	-5.953500	-3.125300	0.223300
H	-7.291100	-3.143400	-0.843700
H	-3.819300	-3.973900	-0.407300
H	1.015300	-4.594600	-0.220900
H	2.382800	-2.739400	0.927900
H	-1.423400	0.140900	1.865800
H	-3.099800	0.040900	1.567100
H	4.605700	1.252700	0.329300
H	5.953500	3.125300	0.223300
H	8.126200	-3.702700	-1.942000
H	8.991600	-1.309800	-2.365500
H	-8.991600	1.309800	-2.365500

(I’’)$_2$ gas phase C_{2h} E = -9563.3165

N	3.368700	2.324300	0.000000
C	3.293500	3.673400	0.000000
N	2.216000	4.467200	0.000000
C	1.081300	3.736700	0.000000
C	0.987900	2.340200	0.000000
C	2.202600	1.600000	0.000000
N	-0.231500	4.180000	0.000000
C	-1.039600	3.056600	0.000000
N	-0.340700	1.935600	0.000000
N	2.237600	0.266500	0.000000
N	-5.809800	-0.632700	0.000000
C	-6.997300	-1.327100	0.000000
N	-8.194000	-0.758400	0.000000
C	-8.120000	0.594000	0.000000

C	-6.979800	1.414200	0.000000
C	-5.689800	0.788700	0.000000
N	-9.183900	1.468600	0.000000
C	-8.647800	2.755500	0.000000
N	-7.334300	2.759200	0.000000
O	-4.565300	1.318600	0.000000
N	-6.908400	-2.690800	0.000000
N	-3.368700	-2.324300	0.000000
C	-3.293500	-3.673400	0.000000
N	-2.216000	-4.467200	0.000000
C	-1.081300	-3.736700	0.000000
C	-0.987900	-2.340200	0.000000
C	-2.202600	-1.600000	0.000000
N	0.231500	-4.180000	0.000000
C	1.039600	-3.056600	0.000000
N	0.340700	-1.935600	0.000000
N	-2.237600	-0.266500	0.000000
N	5.809800	0.632700	0.000000
C	6.997300	1.327100	0.000000
N	8.194000	0.758400	0.000000
C	8.120000	-0.594000	0.000000
C	6.979800	-1.414200	0.000000
C	5.689800	-0.788700	0.000000
N	9.183900	-1.468600	0.000000
C	8.647800	-2.755500	0.000000
N	7.334300	-2.759200	0.000000
O	4.565300	-1.318600	0.000000
N	6.908400	2.690800	0.000000
H	4.249200	4.199500	0.000000
H	-0.530700	5.147500	0.000000
H	-2.121400	3.115500	0.000000
H	1.379500	-0.297000	0.000000
H	3.127300	-0.252500	0.000000
H	-4.926600	-1.187300	0.000000
H	7.763500	3.226200	0.000000
H	-9.283300	3.630900	0.000000
H	-6.017300	-3.161200	0.000000
H	-7.763500	-3.226200	0.000000
H	-4.249200	-4.199500	0.000000
H	0.530700	-5.147500	0.000000
H	2.121400	-3.115500	0.000000
H	-1.379500	0.297000	0.000000
H	-3.127300	0.252500	0.000000
H	4.926600	1.187300	0.000000
H	6.017300	3.161200	0.000000
H	9.283300	-3.630900	0.000000
H	10.161400	-1.204200	0.000000
H	-10.161400	1.204200	0.000000

(I'')$_2$ water C$_2$ E = -9610.9181

N	3.457100	2.188400	0.526700
C	3.422700	3.512500	0.266000
N	2.354700	4.321600	0.186300
C	1.205000	3.644000	0.406600
C	1.087300	2.273800	0.687100
C	2.284400	1.515300	0.738900
N	-0.089600	4.119700	0.389600
C	-0.916900	3.052800	0.649600
N	-0.251800	1.921000	0.836800
N	2.317500	0.194900	0.993600
N	-5.884800	-0.667700	0.091800
C	-7.023600	-1.388800	-0.186500
N	-8.123700	-0.860300	-0.715900
C	-8.004100	0.469100	-0.953600
C	-6.908900	1.301600	-0.675500
C	-5.737100	0.720000	-0.105200
N	-8.950400	1.298400	-1.506300
C	-8.409700	2.569500	-1.542200
N	-7.182800	2.616400	-1.050800
O	-4.664100	1.294000	0.206100
N	-6.969700	-2.735900	0.065800
N	-3.457100	-2.188400	0.526700
C	-3.422700	-3.512500	0.266000
N	-2.354700	-4.321600	0.186300
C	-1.205000	-3.644000	0.406600
C	-1.087300	-2.273800	0.687100
C	-2.284400	-1.515300	0.738900
N	0.089600	-4.119700	0.389600
C	0.916900	-3.052800	0.649600
N	0.251800	-1.921000	0.836800
N	-2.317500	-0.194900	0.993600
N	5.884800	0.667700	0.091800
C	7.023600	1.388800	-0.186500
N	8.123700	0.860300	-0.715900
C	8.004100	-0.469100	-0.953600
C	6.908900	-1.301600	-0.675500
C	5.737100	-0.720000	-0.105200
N	8.950400	-1.298400	-1.506300
C	8.409700	-2.569500	-1.542200
N	7.182800	-2.616400	-1.050800
O	4.664100	-1.294000	0.206100
N	6.969700	2.735900	0.065800
H	4.387500	3.980800	0.088200
H	-0.379500	5.074400	0.208700
H	-1.992200	3.159700	0.685900
H	1.448600	-0.353200	0.991600
H	3.187900	-0.319700	0.808400
H	-5.020800	-1.198300	0.357000
H	7.862900	3.215600	0.022000

H	-8.964600	3.407700	-1.938600
H	-6.372400	-3.043800	0.825200
H	-7.862900	-3.215600	0.022000
H	-4.387500	-3.980800	0.088200
H	0.379500	-5.074400	0.208700
H	1.992200	-3.159700	0.685900
H	-1.448600	0.353200	0.991600
H	-3.187900	0.319700	0.808400
H	5.020800	1.198300	0.357000
H	6.372400	3.043800	0.825200
H	8.964600	-3.407700	-1.938600
H	9.874100	-1.027500	-1.825500
H	-9.874100	1.027500	-1.825500

(I'')$_2$ water C_{2h} E = -9609.9236

N	3.466300	2.232800	0.000000
C	3.431400	3.581600	0.000000
N	2.366800	4.397900	0.000000
C	1.209900	3.695800	0.000000
C	1.086700	2.299000	0.000000
C	2.284600	1.537100	0.000000
N	-0.085100	4.171300	0.000000
C	-0.918200	3.077500	0.000000
N	-0.255800	1.928500	0.000000
N	2.304900	0.197700	0.000000
N	-5.979100	-0.678000	0.000000
C	-7.166500	-1.381400	0.000000
N	-8.368600	-0.802100	0.000000
C	-8.300300	0.549600	0.000000
C	-7.153100	1.358300	0.000000
C	-5.874800	0.728400	0.000000
N	-9.355700	1.430600	0.000000
C	-8.824200	2.707100	0.000000
N	-7.502200	2.709400	0.000000
O	-4.746700	1.281800	0.000000
N	-7.083200	-2.733100	0.000000
N	-3.466300	-2.232800	0.000000
C	-3.431400	-3.581600	0.000000
N	-2.366800	-4.397900	0.000000
C	-1.209900	-3.695800	0.000000
C	-1.086700	-2.299000	0.000000
C	-2.284600	-1.537100	0.000000
N	0.085100	-4.171300	0.000000
C	0.918200	-3.077500	0.000000
N	0.255800	-1.928500	0.000000
N	-2.304900	-0.197700	0.000000
N	5.979100	0.678000	0.000000
C	7.166500	1.381400	0.000000
N	8.368600	0.802100	0.000000

C	8.300300	-0.549600	0.000000
C	7.153100	-1.358300	0.000000
C	5.874800	-0.728400	0.000000
N	9.355700	-1.430600	0.000000
C	8.824200	-2.707100	0.000000
N	7.502200	-2.709400	0.000000
O	4.746700	-1.281800	0.000000
N	7.083200	2.733100	0.000000
H	4.397500	4.081800	0.000000
H	-0.371300	5.144100	0.000000
H	-1.994500	3.179300	0.000000
H	1.438700	-0.353700	0.000000
H	3.201000	-0.306200	0.000000
H	-5.083200	-1.215900	0.000000
H	7.932700	3.280900	0.000000
H	-9.458200	3.582100	0.000000
H	-6.198000	-3.215300	0.000000
H	-7.932700	-3.280900	0.000000
H	-4.397500	-4.081800	0.000000
H	0.371300	-5.144100	0.000000
H	1.994500	-3.179300	0.000000
H	-1.438700	0.353700	0.000000
H	-3.201000	0.306200	0.000000
H	5.083200	1.215900	0.000000
H	6.198000	3.215300	0.000000
H	9.458200	-3.582100	0.000000
H	10.340400	-1.188000	0.000000
H	-10.340400	1.188000	0.000000

$(II)_2$ water C_2 E = -9606.8426

N	-4.094200	-0.458800	-0.523100
C	-5.456900	-0.338300	-0.677000
N	-6.062300	0.788500	-1.042400
C	-5.192400	1.805200	-1.262700
C	-3.791100	1.776700	-1.173600
C	-3.164100	0.565200	-0.763700
N	-5.496900	3.087100	-1.652200
C	-4.299600	3.767300	-1.788300
N	-3.248900	3.015100	-1.510700
O	-1.936000	0.346900	-0.603300
N	-6.204600	-1.449700	-0.381000
H	-6.425200	3.462100	-1.814000
H	-3.715600	-1.346200	-0.116000
N	4.094200	0.458800	-0.523100
C	5.456900	0.338300	-0.677000
N	6.062300	-0.788500	-1.042400
C	5.192400	-1.805200	-1.262700
C	3.791100	-1.776700	-1.173600
C	3.164100	-0.565200	-0.763700

N	5.496900	-3.087100	-1.652200
C	4.299600	-3.767300	-1.788300
N	3.248900	-3.015100	-1.510700
O	1.936000	-0.346900	-0.603300
N	6.204600	1.449700	-0.381000
H	6.425200	-3.462100	-1.814000
H	3.715600	1.346200	-0.116000
N	-0.346300	4.017600	0.865800
C	-0.051700	5.126000	1.571500
N	1.145600	5.546900	2.023900
C	2.128400	4.689900	1.672700
C	1.978900	3.509800	0.926800
C	0.661800	3.165000	0.519800
N	3.475800	4.765200	1.965700
C	4.076500	3.666100	1.407000
N	3.216800	2.883300	0.767200
N	0.373700	2.056900	-0.189900
H	3.937600	5.493100	2.499600
N	0.346300	-4.017600	0.865800
C	0.051700	-5.126000	1.571500
N	-1.145600	-5.546900	2.023900
C	-2.128400	-4.689900	1.672700
C	-1.978900	-3.509800	0.926800
C	-0.661800	-3.165000	0.519800
N	-3.475800	-4.765200	1.965700
C	-4.076500	-3.666100	1.407000
N	-3.216800	-2.883300	0.767200
N	-0.373700	-2.056900	-0.189900
H	-3.937600	-5.493100	2.499600
H	-0.598900	1.777300	-0.335900
H	1.073900	1.319700	-0.314500
H	-1.073900	-1.319700	-0.314500
H	0.598900	-1.777300	-0.335900
H	-7.174400	-1.403000	-0.676200
H	-5.770500	-2.353600	-0.534300
H	5.770500	2.353600	-0.534300
H	7.174400	1.403000	-0.676200
H	5.136800	3.484000	1.505600
H	-5.136800	-3.484000	1.505600
H	-4.272100	4.803100	-2.095300
H	4.272100	-4.803100	-2.095300
H	-0.899000	5.764500	1.813300
H	0.899000	-5.764500	1.813300

$(II)_2$ water C_{2h} E = -9599.7698

N	-4.344200	-0.341000	0.000000
C	-5.719900	-0.225500	0.000000
N	-6.362500	0.942700	0.000000
C	-5.523100	2.003100	0.000000

C	-4.116700	1.999100	0.000000
C	-3.442000	0.742100	0.000000
N	-5.876900	3.330300	0.000000
C	-4.702600	4.064100	0.000000
N	-3.623800	3.304200	0.000000
O	-2.203500	0.518900	0.000000
N	-6.440300	-1.372000	0.000000
H	-6.821300	3.699800	0.000000
H	-3.927200	-1.296000	0.000000
N	4.344200	0.341000	0.000000
C	5.719900	0.225500	0.000000
N	6.362500	-0.942700	0.000000
C	5.523100	-2.003100	0.000000
C	4.116700	-1.999100	0.000000
C	3.442000	-0.742100	0.000000
N	5.876900	-3.330300	0.000000
C	4.702600	-4.064100	0.000000
N	3.623800	-3.304200	0.000000
O	2.203500	-0.518900	0.000000
N	6.440300	1.372000	0.000000
H	6.821300	-3.699800	0.000000
H	3.927200	1.296000	0.000000
N	-0.265000	4.031700	0.000000
C	-0.022300	5.351400	0.000000
N	1.165600	5.989300	0.000000
C	2.187400	5.107600	0.000000
C	2.092300	3.703800	0.000000
C	0.780100	3.156500	0.000000
N	3.540000	5.379300	0.000000
C	4.195300	4.176300	0.000000
N	3.371300	3.136200	0.000000
N	0.502500	1.838100	0.000000
H	3.971600	6.296800	0.000000
N	0.265000	-4.031700	0.000000
C	0.022300	-5.351400	0.000000
N	-1.165600	-5.989300	0.000000
C	-2.187400	-5.107600	0.000000
C	-2.092300	-3.703800	0.000000
C	-0.780100	-3.156500	0.000000
N	-3.540000	-5.379300	0.000000
C	-4.195300	-4.176300	0.000000
N	-3.371300	-3.136200	0.000000
N	-0.502500	-1.838100	0.000000
H	-3.971600	-6.296800	0.000000
H	-0.475600	1.541100	0.000000
H	1.236900	1.126200	0.000000
H	-1.236900	-1.126200	0.000000
H	0.475600	-1.541100	0.000000
H	-7.449600	-1.320900	0.000000
H	-6.002400	-2.278000	0.000000

H	6.002400	2.278000	0.000000
H	7.449600	1.320900	0.000000
H	5.274800	4.137300	0.000000
H	-5.274800	-4.137300	0.000000
H	-4.715300	5.145000	0.000000
H	4.715300	-5.145000	0.000000
H	-0.903800	5.989900	0.000000
H	0.903800	-5.989900	0.000000

(II')$_2$ gas phase C_2 E = -9562.1110

N	-3.734500	-0.931400	-0.028300
C	-4.696600	-0.541900	0.868500
N	-4.931000	0.713100	1.215700
C	-4.089500	1.583300	0.600700
C	-3.055600	1.305100	-0.302400
C	-2.809400	-0.059800	-0.676500
N	-4.036900	2.950300	0.790000
C	-2.981500	3.429500	0.024500
N	-2.379700	2.467900	-0.642500
O	-1.943600	-0.522700	-1.427800
N	-5.484400	-1.552100	1.401500
H	-4.640600	3.482600	1.404400
N	3.734500	0.931400	-0.028300
C	4.696600	0.541900	0.868500
N	4.931000	-0.713100	1.215700
C	4.089500	-1.583300	0.600700
C	3.055600	-1.305100	-0.302400
C	2.809400	0.059800	-0.676500
N	4.036900	-2.950300	0.790000
C	2.981500	-3.429500	0.024500
N	2.379700	-2.467900	-0.642500
O	1.943600	0.522700	-1.427800
N	5.484400	1.552100	1.401500
H	4.640600	-3.482600	1.404400
N	-0.041300	5.138900	-0.286700
C	0.367000	6.373600	0.045800
N	1.619000	6.843900	0.218000
C	2.517900	5.860900	0.043600
C	2.260300	4.519000	-0.275400
C	0.893900	4.159000	-0.492900
N	3.899800	5.945600	0.153500
C	4.405800	4.685000	-0.084000
N	3.455900	3.799300	-0.338800
N	0.493900	2.948100	-0.889600
H	4.421800	6.788600	0.359000
N	0.041300	-5.138900	-0.286700
C	-0.367000	-6.373600	0.045800
N	-1.619000	-6.843900	0.218000
C	-2.517900	-5.860900	0.043600

C	-2.260300	-4.519000	-0.275400
C	-0.893900	-4.159000	-0.492900
N	-3.899800	-5.945600	0.153500
C	-4.405800	-4.685000	-0.084000
N	-3.455900	-3.799300	-0.338800
N	-0.493900	-2.948100	-0.889600
H	-4.421800	-6.788600	0.359000
H	-0.514500	2.754400	-0.969100
H	1.148700	2.186400	-1.092000
H	-1.148700	-2.186400	-1.092000
H	0.514500	-2.754400	-0.969100
H	-6.015400	-1.252100	2.212500
H	-5.031600	-2.454600	1.510100
H	5.031600	2.454600	1.510100
H	6.015400	1.252100	2.212500
H	5.467000	4.474700	-0.067500
H	-5.467000	-4.474700	-0.067500
H	-2.668000	4.464800	0.022200
H	2.668000	-4.464800	0.022200
H	-0.428400	7.103400	0.191700
H	0.428400	-7.103400	0.191700
H	3.626400	1.946300	-0.243400
H	-3.626400	-1.946300	-0.243400

(II’)$_2$ gas phase C_{2h} E = -9558.3743

N	-3.955200	-0.847800	0.000000
C	-5.268300	-0.438000	0.000000
N	-5.662400	0.827700	0.000000
C	-4.619300	1.694700	0.000000
C	-3.252000	1.401700	0.000000
C	-2.829100	0.031500	0.000000
N	-4.684700	3.075100	0.000000
C	-3.375500	3.546900	0.000000
N	-2.500900	2.566100	0.000000
O	-1.680100	-0.418200	0.000000
N	-6.220900	-1.421700	0.000000
H	-5.535100	3.624700	0.000000
N	3.955200	0.847800	0.000000
C	5.268300	0.438000	0.000000
N	5.662400	-0.827700	0.000000
C	4.619300	-1.694700	0.000000
C	3.252000	-1.401700	0.000000
C	2.829100	-0.031500	0.000000
N	4.684700	-3.075100	0.000000
C	3.375500	-3.546900	0.000000
N	2.500900	-2.566100	0.000000
O	1.680100	0.418200	0.000000
N	6.220900	1.421700	0.000000
H	5.535100	-3.624700	0.000000

N	-0.054700	5.217400	0.000000
C	0.392400	6.480800	0.000000
N	1.661200	6.938500	0.000000
C	2.529500	5.913600	0.000000
C	2.237400	4.539900	0.000000
C	0.849300	4.186100	0.000000
N	3.915100	5.988800	0.000000
C	4.389800	4.695800	0.000000
N	3.423600	3.792200	0.000000
N	0.391900	2.934400	0.000000
H	4.456400	6.844200	0.000000
N	0.054700	-5.217400	0.000000
C	-0.392400	-6.480800	0.000000
N	-1.661200	-6.938500	0.000000
C	-2.529500	-5.913600	0.000000
C	-2.237400	-4.539900	0.000000
C	-0.849300	-4.186100	0.000000
N	-3.915100	-5.988800	0.000000
C	-4.389800	-4.695800	0.000000
N	-3.423600	-3.792200	0.000000
N	-0.391900	-2.934400	0.000000
H	-4.456400	-6.844200	0.000000
H	-0.627400	2.777300	0.000000
H	1.008700	2.117000	0.000000
H	-1.008700	-2.117000	0.000000
H	0.627400	-2.777300	0.000000
H	-7.191700	-1.146900	0.000000
H	-5.971100	-2.396100	0.000000
H	5.971100	2.396100	0.000000
H	7.191700	1.146900	0.000000
H	5.452900	4.492900	0.000000
H	-5.452900	-4.492900	0.000000
H	-3.124000	4.598900	0.000000
H	3.124000	-4.598900	0.000000
H	-0.379800	7.249300	0.000000
H	0.379800	-7.249300	0.000000
H	3.727800	1.863900	0.000000
H	-3.727800	-1.863900	0.000000

(II')$_2$ water C$_2$ E = -9610.0965

N	-3.242500	-0.874200	0.283000
C	-3.867100	-0.443200	1.430300
N	-4.014400	0.840700	1.750800
C	-3.486900	1.675800	0.822500
C	-2.824100	1.340600	-0.369100
C	-2.658100	-0.040200	-0.691400
N	-3.470400	3.051000	0.864900
C	-2.814900	3.490200	-0.266900
N	-2.411200	2.493000	-1.037000

O	-2.081800	-0.549200	-1.681500
N	-4.395300	-1.410700	2.242200
H	-3.855000	3.633200	1.600900
N	3.242500	0.874200	0.283000
C	3.867100	0.443200	1.430300
N	4.014400	-0.840700	1.750800
C	3.486900	-1.675800	0.822500
C	2.824100	-1.340600	-0.369100
C	2.658100	0.040200	-0.691400
N	3.470400	-3.051000	0.864900
C	2.814900	-3.490200	-0.266900
N	2.411200	-2.493000	-1.037000
O	2.081800	0.549200	-1.681500
N	4.395300	1.410700	2.242200
H	3.855000	-3.633200	1.600900
N	-0.036200	5.256400	-0.999000
C	0.326000	6.471100	-0.545300
N	1.506500	6.862300	-0.026100
C	2.379500	5.833900	0.031600
C	2.140000	4.514900	-0.385200
C	0.867300	4.233200	-0.947400
N	3.670300	5.828700	0.523500
C	4.147900	4.548100	0.403800
N	3.260400	3.721500	-0.135900
N	0.517600	3.029800	-1.444800
H	4.171300	6.619600	0.912900
N	0.036200	-5.256400	-0.999000
C	-0.326000	-6.471100	-0.545300
N	-1.506500	-6.862300	-0.026100
C	-2.379500	-5.833900	0.031600
C	-2.140000	-4.514900	-0.385200
C	-0.867300	-4.233200	-0.947400
N	-3.670300	-5.828700	0.523500
C	-4.147900	-4.548100	0.403800
N	-3.260400	-3.721500	-0.135900
N	-0.517600	-3.029800	-1.444800
H	-4.171300	-6.619600	0.912900
H	-0.477600	2.838000	-1.595000
H	1.159200	2.233000	-1.417700
H	-1.159200	-2.233000	-1.417700
H	0.477600	-2.838000	-1.595000
H	-4.655100	-1.106300	3.174100
H	-3.995200	-2.341400	2.194300
H	3.995200	2.341400	2.194300
H	4.655100	1.106300	3.174100
H	5.142200	4.274400	0.725900
H	-5.142200	-4.274400	0.725900
H	-2.631200	4.537300	-0.456800
H	2.631200	-4.537300	-0.456800
H	-0.439000	7.241600	-0.612400

H	0.439000	-7.241600	-0.612400
H	3.232100	1.899300	0.077400
H	-3.232100	-1.899300	0.077400

(II')$_2$ water C_{2h} E = -9606.1398

N	-3.967800	-0.853600	0.000000
C	-5.290500	-0.456500	0.000000
N	-5.680900	0.820300	0.000000
C	-4.642700	1.689400	0.000000
C	-3.273700	1.390800	0.000000
C	-2.863400	0.025400	0.000000
N	-4.707000	3.065400	0.000000
C	-3.408500	3.538500	0.000000
N	-2.520000	2.561000	0.000000
O	-1.693900	-0.420200	0.000000
N	-6.229200	-1.432500	0.000000
H	-5.551200	3.627300	0.000000
N	3.967800	0.853600	0.000000
C	5.290500	0.456500	0.000000
N	5.680900	-0.820300	0.000000
C	4.642700	-1.689400	0.000000
C	3.273700	-1.390800	0.000000
C	2.863400	-0.025400	0.000000
N	4.707000	-3.065400	0.000000
C	3.408500	-3.538500	0.000000
N	2.520000	-2.561000	0.000000
O	1.693900	0.420200	0.000000
N	6.229200	1.432500	0.000000
H	5.551200	-3.627300	0.000000
N	-0.051200	5.239400	0.000000
C	0.404100	6.504800	0.000000
N	1.677600	6.946000	0.000000
C	2.550900	5.914600	0.000000
C	2.234000	4.546600	0.000000
C	0.851000	4.207900	0.000000
N	3.930100	5.967900	0.000000
C	4.386100	4.674600	0.000000
N	3.405900	3.780100	0.000000
N	0.386900	2.950000	0.000000
H	4.502200	6.804900	0.000000
N	0.051200	-5.239400	0.000000
C	-0.404100	-6.504800	0.000000
N	-1.677600	-6.946000	0.000000
C	-2.550900	-5.914600	0.000000
C	-2.234000	-4.546600	0.000000
C	-0.851000	-4.207900	0.000000
N	-3.930100	-5.967900	0.000000
C	-4.386100	-4.674600	0.000000
N	-3.405900	-3.780100	0.000000

N	-0.386900	-2.950000	0.000000
H	-4.502200	-6.804900	0.000000
H	-0.633400	2.791500	0.000000
H	1.015500	2.140900	0.000000
H	-1.015500	-2.140900	0.000000
H	0.633400	-2.791500	0.000000
H	-7.207000	-1.177000	0.000000
H	-5.984300	-2.409700	0.000000
H	5.984300	2.409700	0.000000
H	7.207000	1.177000	0.000000
H	5.444900	4.459600	0.000000
H	-5.444900	-4.459600	0.000000
H	-3.175900	4.593700	0.000000
H	3.175900	-4.593700	0.000000
H	-0.362400	7.277400	0.000000
H	0.362400	-7.277400	0.000000
H	3.740000	1.870300	0.000000
H	-3.740000	-1.870300	0.000000

$(H \bullet I)_2$ gas phase C_2 E = -9457.9165

N	-0.330600	-3.881900	-0.201700
C	-0.152900	-5.178200	0.120900
N	0.990200	-5.822600	0.397700
C	2.037900	-4.996600	0.342900
C	2.016600	-3.631300	0.042600
C	0.751100	-3.045300	-0.261700
N	3.374700	-5.311900	0.591300
C	4.116500	-4.192000	0.458200
N	3.332100	-3.161600	0.127800
N	0.581500	-1.770200	-0.593600
N	-3.061400	-2.605100	-0.361300
C	-4.205700	-3.361300	-0.170800
N	-5.397900	-2.836600	0.075900
C	-5.386300	-1.492400	0.124200
C	-4.302600	-0.627600	-0.084700
C	-3.031500	-1.199500	-0.364800
N	-6.459800	-0.660900	0.370900
C	-6.000400	0.636700	0.307300
N	-4.705700	0.702400	0.035700
O	-1.958400	-0.593900	-0.592600
N	-4.059000	-4.718000	-0.209300
N	0.330600	3.881900	-0.201700
C	0.152900	5.178200	0.120900
N	-0.990200	5.822600	0.397700
C	-2.037900	4.996600	0.342900
C	-2.016600	3.631300	0.042600
C	-0.751100	3.045300	-0.261700
N	-3.374700	5.311900	0.591300
C	-4.116500	4.192000	0.458200

N	-3.332100	3.161600	0.127800
N	-0.581500	1.770200	-0.593600
N	3.061400	2.605100	-0.361300
C	4.205700	3.361300	-0.170800
N	5.397900	2.836600	0.075900
C	5.386300	1.492400	0.124200
C	4.302600	0.627600	-0.084700
C	3.031500	1.199500	-0.364800
N	6.459800	0.660900	0.370900
C	6.000400	-0.636700	0.307300
N	4.705700	-0.702400	0.035700
O	1.958400	0.593900	-0.592600
N	4.059000	4.718000	-0.209300
H	-1.056300	-5.782200	0.170600
H	3.722200	-6.235200	0.836900
H	5.184900	-4.139300	0.601200
H	1.338900	-1.079400	-0.579800
H	-0.375100	-1.400700	-0.689600
H	-2.136300	-3.080200	-0.410100
H	-7.411300	-0.962900	0.555900
H	-6.657800	1.479800	0.465900
H	-3.308000	-5.119900	-0.756600
H	-4.920400	-5.252900	-0.175700
H	1.056300	5.782200	0.170600
H	-3.722200	6.235200	0.836900
H	-5.184900	4.139300	0.601200
H	-1.338900	1.079400	-0.579800
H	0.375100	1.400700	-0.689600
H	2.136300	3.080200	-0.410100
H	7.411300	0.962900	0.555900
H	6.657800	-1.479800	0.465900
H	3.308000	5.119900	-0.756600
H	4.920400	5.252900	-0.175700
H	3.731100	-2.172700	0.021400
H	-3.731100	2.172700	0.021400

(H • I)$_2$ gas phase C$_{2h}$ E = -9458.2365

N	-0.288900	-3.936000	0.000000
C	-0.067600	-5.265100	0.000000
N	1.100200	-5.924200	0.000000
C	2.133800	-5.078200	0.000000
C	2.073800	-3.682300	0.000000
C	0.779700	-3.078900	0.000000
N	3.491400	-5.402200	0.000000
C	4.209800	-4.259100	0.000000
N	3.389100	-3.204300	0.000000
N	0.576900	-1.767800	0.000000
N	-3.083100	-2.597900	0.000000
C	-4.251900	-3.344400	0.000000

N	-5.464700	-2.803900	0.000000
C	-5.449100	-1.460800	0.000000
C	-4.336400	-0.606900	0.000000
C	-3.043100	-1.192000	0.000000
N	-6.541200	-0.616600	0.000000
C	-6.064400	0.676800	0.000000
N	-4.741200	0.728800	0.000000
O	-1.941000	-0.595100	0.000000
N	-4.127200	-4.693300	0.000000
N	0.288900	3.936000	0.000000
C	0.067600	5.265100	0.000000
N	-1.100200	5.924200	0.000000
C	-2.133800	5.078200	0.000000
C	-2.073800	3.682300	0.000000
C	-0.779700	3.078900	0.000000
N	-3.491400	5.402200	0.000000
C	-4.209800	4.259100	0.000000
N	-3.389100	3.204300	0.000000
N	-0.576900	1.767800	0.000000
N	3.083100	2.597900	0.000000
C	4.251900	3.344400	0.000000
N	5.464700	2.803900	0.000000
C	5.449100	1.460800	0.000000
C	4.336400	0.606900	0.000000
C	3.043100	1.192000	0.000000
N	6.541200	0.616600	0.000000
C	6.064400	-0.676800	0.000000
N	4.741200	-0.728800	0.000000
O	1.941000	0.595100	0.000000
N	4.127200	4.693300	0.000000
H	-0.954300	-5.896100	0.000000
H	3.868100	-6.346500	0.000000
H	5.288000	-4.208400	0.000000
H	1.334300	-1.077200	0.000000
H	-0.385800	-1.399200	0.000000
H	-2.162700	-3.078200	0.000000
H	-7.513700	-0.908000	0.000000
H	-6.731600	1.527100	0.000000
H	-3.230600	-5.154700	0.000000
H	-4.969800	-5.253600	0.000000
H	0.954300	5.896100	0.000000
H	-3.868100	6.346500	0.000000
H	-5.288000	4.208400	0.000000
H	-1.334300	1.077200	0.000000
H	0.385800	1.399200	0.000000
H	2.162700	3.078200	0.000000
H	7.513700	0.908000	0.000000
H	6.731600	-1.527100	0.000000
H	3.230600	5.154700	0.000000
H	4.969800	5.253600	0.000000

H	3.771500	-2.202500	0.000000
H	-3.771500	2.202500	0.000000

(H • I)$_2$ water C$_2$ E = -9603.7313

N	-0.341200	-3.872200	-0.810300
C	-0.165300	-5.207200	-0.817800
N	0.982500	-5.899500	-0.735900
C	2.042000	-5.079900	-0.607200
C	2.003700	-3.684100	-0.572400
C	0.744400	-3.046500	-0.712700
N	3.378400	-5.428300	-0.474100
C	4.109600	-4.303600	-0.351500
N	3.312100	-3.235800	-0.412400
N	0.567800	-1.722700	-0.747600
N	-2.838000	-2.547100	-0.035600
C	-3.698500	-3.215400	0.811900
N	-4.734000	-2.631300	1.416300
C	-4.832300	-1.312400	1.140400
C	-4.000900	-0.543000	0.313100
C	-2.915800	-1.181300	-0.340200
N	-5.769000	-0.427800	1.624700
C	-5.480100	0.811100	1.100800
N	-4.422300	0.785200	0.304200
O	-2.068700	-0.655800	-1.112900
N	-3.426700	-4.523900	1.057800
N	0.341200	3.872200	-0.810300
C	0.165300	5.207200	-0.817800
N	-0.982500	5.899500	-0.735900
C	-2.042000	5.079900	-0.607200
C	-2.003700	3.684100	-0.572400
C	-0.744400	3.046500	-0.712700
N	-3.378400	5.428300	-0.474100
C	-4.109600	4.303600	-0.351500
N	-3.312100	3.235800	-0.412400
N	-0.567800	1.722700	-0.747600
N	2.838000	2.547100	-0.035600
C	3.698500	3.215400	0.811900
N	4.734000	2.631300	1.416300
C	4.832300	1.312400	1.140400
C	4.000900	0.543000	0.313100
C	2.915800	1.181300	-0.340200
N	5.769000	0.427800	1.624700
C	5.480100	-0.811100	1.100800
N	4.422300	-0.785200	0.304200
O	2.068700	0.655800	-1.112900
N	3.426700	4.523900	1.057800
H	-1.072400	-5.799600	-0.910500
H	3.752300	-6.371700	-0.461800
H	5.178200	-4.275500	-0.215400

H	1.346600	-1.061000	-0.798100
H	-0.371900	-1.352500	-0.936200
H	-2.014500	-3.058700	-0.421000
H	-6.528100	-0.646000	2.261000
H	-6.069400	1.684500	1.337200
H	-2.811700	-5.038900	0.441700
H	-4.138100	-5.063600	1.536000
H	1.072400	5.799600	-0.910500
H	-3.752300	6.371700	-0.461800
H	-5.178200	4.275500	-0.215400
H	-1.346600	1.061000	-0.798100
H	0.371900	1.352500	-0.936200
H	2.014500	3.058700	-0.421000
H	6.528100	0.646000	2.261000
H	6.069400	-1.684500	1.337200
H	2.811700	5.038900	0.441700
H	4.138100	5.063600	1.536000
H	3.665100	-2.244600	-0.240900
H	-3.665100	2.244600	-0.240900

$(H \bullet I)_2$ water C_{2h} E = -9602.0458

N	-0.290300	-3.967900	0.000000
C	-0.084400	-5.298500	0.000000
N	1.080900	-5.970100	0.000000
C	2.134400	-5.131000	0.000000
C	2.071800	-3.738000	0.000000
C	0.790500	-3.125900	0.000000
N	3.485400	-5.452600	0.000000
C	4.203300	-4.312900	0.000000
N	3.381900	-3.260800	0.000000
N	0.602500	-1.809900	0.000000
N	-3.043000	-2.611800	0.000000
C	-4.206300	-3.358600	0.000000
N	-5.426700	-2.815400	0.000000
C	-5.409100	-1.463400	0.000000
C	-4.289300	-0.620800	0.000000
C	-3.001700	-1.210800	0.000000
N	-6.490800	-0.611500	0.000000
C	-6.004100	0.677900	0.000000
N	-4.680500	0.718500	0.000000
O	-1.897100	-0.610600	0.000000
N	-4.069600	-4.702800	0.000000
N	0.290300	3.967900	0.000000
C	0.084400	5.298500	0.000000
N	-1.080900	5.970100	0.000000
C	-2.134400	5.131000	0.000000
C	-2.071800	3.738000	0.000000
C	-0.790500	3.125900	0.000000
N	-3.485400	5.452600	0.000000

C	-4.203300	4.312900	0.000000
N	-3.381900	3.260800	0.000000
N	-0.602500	1.809900	0.000000
N	3.043000	2.611800	0.000000
C	4.206300	3.358600	0.000000
N	5.426700	2.815400	0.000000
C	5.409100	1.463400	0.000000
C	4.289300	0.620800	0.000000
C	3.001700	1.210800	0.000000
N	6.490800	0.611500	0.000000
C	6.004100	-0.677900	0.000000
N	4.680500	-0.718500	0.000000
O	1.897100	0.610600	0.000000
N	4.069600	4.702800	0.000000
H	-0.979900	-5.915300	0.000000
H	3.878600	-6.388300	0.000000
H	5.279600	-4.261600	0.000000
H	1.369600	-1.133500	0.000000
H	-0.353600	-1.426700	0.000000
H	-2.126000	-3.099900	0.000000
H	-7.468600	-0.880900	0.000000
H	-6.661500	1.534600	0.000000
H	-3.164100	-5.149300	0.000000
H	-4.895900	-5.285400	0.000000
H	0.979900	5.915300	0.000000
H	-3.878600	6.388300	0.000000
H	-5.279600	4.261600	0.000000
H	-1.369600	1.133500	0.000000
H	0.353600	1.426700	0.000000
H	2.126000	3.099900	0.000000
H	7.468600	0.880900	0.000000
H	6.661500	-1.534600	0.000000
H	3.164100	5.149300	0.000000
H	4.895900	5.285400	0.000000
H	3.739700	-2.264600	0.000000
H	-3.739700	2.264600	0.000000

(H • II)$_2$ / (III)$_2$ gas phase C$_2$ E = -9473.7350

N	-4.152800	-0.352400	-0.411600
C	-5.519900	-0.136000	-0.419500
N	-6.072600	1.059300	-0.294300
C	-5.170700	2.051700	-0.168500
C	-3.770800	1.955600	-0.195600
C	-3.191200	0.664900	-0.331900
N	-5.430700	3.397000	-0.016300
C	-4.216100	4.047800	0.041600
N	-3.191800	3.217100	-0.062100
O	-1.969300	0.371800	-0.375000
N	-6.313600	-1.250600	-0.525900

H	-6.352100	3.820600	0.033800
H	-3.787300	-1.323700	-0.352400
N	4.152800	0.352400	-0.411600
C	5.519900	0.136000	-0.419500
N	6.072600	-1.059300	-0.294300
C	5.170700	-2.051700	-0.168500
C	3.770800	-1.955600	-0.195600
C	3.191200	-0.664900	-0.331900
N	5.430700	-3.397000	-0.016300
C	4.216100	-4.047800	0.041600
N	3.191800	-3.217100	-0.062100
O	1.969300	-0.371800	-0.375000
N	6.313600	1.250600	-0.525900
H	6.352100	-3.820600	0.033800
H	3.787300	1.323700	-0.352400
N	-0.449700	4.007300	0.167700
C	-0.250800	5.314400	0.544300
N	0.914700	5.875400	0.754000
C	1.946300	5.021000	0.552700
C	1.874800	3.678900	0.153200
C	0.584700	3.114900	-0.040700
N	3.290000	5.276200	0.708400
C	3.961300	4.112600	0.407300
N	3.146500	3.125700	0.062100
N	0.337200	1.863600	-0.381200
H	3.695600	6.160600	1.001000
H	-1.450600	3.676100	0.058800
N	0.449700	-4.007300	0.167700
C	0.250800	-5.314400	0.544300
N	-0.914700	-5.875400	0.754000
C	-1.946300	-5.021000	0.552700
C	-1.874800	-3.678900	0.153200
C	-0.584700	-3.114900	-0.040700
N	-3.290000	-5.276200	0.708400
C	-3.961300	-4.112600	0.407300
N	-3.146500	-3.125700	0.062100
N	-0.337200	-1.863600	-0.381200
H	-3.695600	-6.160600	1.001000
H	1.450600	-3.676100	0.058800
H	-0.621300	1.473200	-0.405100
H	1.105700	1.178200	-0.431100
H	-1.105700	-1.178200	-0.431100
H	0.621300	-1.473200	-0.405100
H	-7.302800	-1.047700	-0.638400
H	-5.973300	-2.018300	-1.094500
H	5.973300	2.018300	-1.094500
H	7.302800	1.047700	-0.638400
H	5.038900	4.040900	0.469900
H	-5.038900	-4.040900	0.469900
H	-4.149300	5.120600	0.157800

H	4.149300	-5.120600	0.157800
H	-1.156600	5.899100	0.674100
H	1.156600	-5.899100	0.674100

(H • II)$_2$ / (III)$_2$ gas phase C$_{2h}$ E = -9472.9715

N	-4.183300	-0.319100	0.000000
C	-5.550200	-0.081800	0.000000
N	-6.082400	1.132400	0.000000
C	-5.166300	2.114800	0.000000
C	-3.767600	1.994200	0.000000
C	-3.206000	0.690100	0.000000
N	-5.405800	3.472800	0.000000
C	-4.181500	4.107800	0.000000
N	-3.171100	3.254800	0.000000
O	-1.987600	0.378200	0.000000
N	-6.370400	-1.161600	0.000000
H	-6.320700	3.912700	0.000000
H	-3.825700	-1.291000	0.000000
N	4.183300	0.319100	0.000000
C	5.550200	0.081800	0.000000
N	6.082400	-1.132400	0.000000
C	5.166300	-2.114800	0.000000
C	3.767600	-1.994200	0.000000
C	3.206000	-0.690100	0.000000
N	5.405800	-3.472800	0.000000
C	4.181500	-4.107800	0.000000
N	3.171100	-3.254800	0.000000
O	1.987600	-0.378200	0.000000
N	6.370400	1.161600	0.000000
H	6.320700	-3.912700	0.000000
H	3.825700	1.291000	0.000000
N	-0.456200	4.047000	0.000000
C	-0.283000	5.408700	0.000000
N	0.872200	6.027100	0.000000
C	1.917800	5.167000	0.000000
C	1.875200	3.764100	0.000000
C	0.592800	3.147400	0.000000
N	3.255700	5.485100	0.000000
C	3.951900	4.298700	0.000000
N	3.162500	3.234000	0.000000
N	0.357600	1.848600	0.000000
H	3.641500	6.425100	0.000000
H	-1.457300	3.687900	0.000000
N	0.456200	-4.047000	0.000000
C	0.283000	-5.408700	0.000000
N	-0.872200	-6.027100	0.000000
C	-1.917800	-5.167000	0.000000
C	-1.875200	-3.764100	0.000000
C	-0.592800	-3.147400	0.000000

N	-3.255700	-5.485100	0.000000
C	-3.951900	-4.298700	0.000000
N	-3.162500	-3.234000	0.000000
N	-0.357600	-1.848600	0.000000
H	-3.641500	-6.425100	0.000000
H	1.457300	-3.687900	0.000000
H	-0.599900	1.456100	0.000000
H	1.132900	1.168900	0.000000
H	-1.132900	-1.168900	0.000000
H	0.599900	-1.456100	0.000000
H	-7.370000	-1.004200	0.000000
H	-6.019500	-2.105300	0.000000
H	6.019500	2.105300	0.000000
H	7.370000	1.004200	0.000000
H	5.033400	4.287600	0.000000
H	-5.033400	-4.287600	0.000000
H	-4.098100	5.185600	0.000000
H	4.098100	-5.185600	0.000000
H	-1.200400	5.989600	0.000000
H	1.200400	-5.989600	0.000000

(H • II)$_2$ / (III)$_2$ water C$_2$ E = -9612.0755

N	-4.156600	-0.363800	-0.223200
C	-5.464600	-0.172200	0.172900
N	-5.963200	1.014500	0.514000
C	-5.056100	2.013800	0.436800
C	-3.718600	1.926500	0.024600
C	-3.205300	0.655300	-0.343000
N	-5.252500	3.343000	0.733300
C	-4.064600	3.998300	0.502200
N	-3.115300	3.181700	0.072700
O	-2.039100	0.384800	-0.739300
N	-6.251600	-1.285000	0.256400
H	-6.113600	3.764100	1.064700
H	-3.807300	-1.334100	-0.367300
N	4.156600	0.363800	-0.223200
C	5.464600	0.172200	0.172900
N	5.963200	-1.014500	0.514000
C	5.056100	-2.013800	0.436800
C	3.718600	-1.926500	0.024600
C	3.205300	-0.655300	-0.343000
N	5.252500	-3.343000	0.733300
C	4.064600	-3.998300	0.502200
N	3.115300	-3.181700	0.072700
O	2.039100	-0.384800	-0.739300
N	6.251600	1.285000	0.256400
H	6.113600	-3.764100	1.064700
H	3.807300	1.334100	-0.367300
N	-0.426200	4.006700	-0.110100

C	-0.238200	5.340900	0.110500
N	0.930600	5.943100	0.179100
C	1.968000	5.080800	0.012200
C	1.892100	3.698500	-0.215100
C	0.604600	3.109800	-0.288700
N	3.310100	5.367300	0.032000
C	3.981200	4.189100	-0.173800
N	3.165600	3.153500	-0.330800
N	0.349600	1.830700	-0.507700
H	3.729500	6.279700	0.177900
H	-1.424900	3.653200	-0.100800
N	0.426200	-4.006700	-0.110100
C	0.238200	-5.340900	0.110500
N	-0.930600	-5.943100	0.179100
C	-1.968000	-5.080800	0.012200
C	-1.892100	-3.698500	-0.215100
C	-0.604600	-3.109800	-0.288700
N	-3.310100	-5.367300	0.032000
C	-3.981200	-4.189100	-0.173800
N	-3.165600	-3.153500	-0.330800
N	-0.349600	-1.830700	-0.507700
H	-3.729500	-6.279700	0.177900
H	1.424900	-3.653200	-0.100800
H	-0.610900	1.459000	-0.562400
H	1.119400	1.156400	-0.611300
H	-1.119400	-1.156400	-0.611300
H	0.610900	-1.459000	-0.562400
H	-7.242200	-1.108600	0.385800
H	-6.042300	-2.066600	-0.352400
H	6.042300	2.066600	-0.352400
H	7.242200	1.108600	0.385800
H	5.059900	4.152600	-0.195800
H	-5.059900	-4.152600	-0.195800
H	-3.955500	5.060100	0.665300
H	3.955500	-5.060100	0.665300
H	-1.147700	5.917700	0.235100
H	1.147700	-5.917700	0.235100

(H • II)$_2$ / (III)$_2$ water C$_{2h}$ E = -9610.7910

N	-4.176700	-0.346600	0.000000
C	-5.540600	-0.119500	0.000000
N	-6.080800	1.101900	0.000000
C	-5.161400	2.091500	0.000000
C	-3.764600	1.964800	0.000000
C	-3.206900	0.661400	0.000000
N	-5.394500	3.448000	0.000000
C	-4.170100	4.079600	0.000000
N	-3.161400	3.222900	0.000000
O	-1.984500	0.355400	0.000000

N	-6.345600	-1.204100	0.000000
H	-6.301500	3.901900	0.000000
H	-3.817000	-1.321200	0.000000
N	4.176700	0.346600	0.000000
C	5.540600	0.119500	0.000000
N	6.080800	-1.101900	0.000000
C	5.161400	-2.091500	0.000000
C	3.764600	-1.964800	0.000000
C	3.206900	-0.661400	0.000000
N	5.394500	-3.448000	0.000000
C	4.170100	-4.079600	0.000000
N	3.161400	-3.222900	0.000000
O	1.984500	-0.355400	0.000000
N	6.345600	1.204100	0.000000
H	6.301500	-3.901900	0.000000
H	3.817000	1.321200	0.000000
N	-0.444100	4.073100	0.000000
C	-0.256000	5.425800	0.000000
N	0.913600	6.030400	0.000000
C	1.952900	5.153100	0.000000
C	1.878100	3.752800	0.000000
C	0.589700	3.160600	0.000000
N	3.295300	5.439500	0.000000
C	3.966500	4.243100	0.000000
N	3.152200	3.194400	0.000000
N	0.339300	1.864200	0.000000
H	3.715000	6.363400	0.000000
H	-1.440700	3.718800	0.000000
N	0.444100	-4.073100	0.000000
C	0.256000	-5.425800	0.000000
N	-0.913600	-6.030400	0.000000
C	-1.952900	-5.153100	0.000000
C	-1.878100	-3.752800	0.000000
C	-0.589700	-3.160600	0.000000
N	-3.295300	-5.439500	0.000000
C	-3.966500	-4.243100	0.000000
N	-3.152200	-3.194400	0.000000
N	-0.339300	-1.864200	0.000000
H	-3.715000	-6.363400	0.000000
H	1.440700	-3.718800	0.000000
H	-0.618600	1.480300	0.000000
H	1.111700	1.183900	0.000000
H	-1.111700	-1.183900	0.000000
H	0.618600	-1.480300	0.000000
H	-7.348500	-1.076100	0.000000
H	-5.980000	-2.143800	0.000000
H	5.980000	2.143800	0.000000
H	7.348500	1.076100	0.000000
H	5.045800	4.207300	0.000000
H	-5.045800	-4.207300	0.000000

H	-4.082700	5.156000	0.000000
H	4.082700	-5.156000	0.000000
H	-1.165600	6.015700	0.000000
H	1.165600	-6.015700	0.000000

(III')$_2$ gas phase C$_2$ E = -9467.8392

N	-6.731400	-1.927800	-0.111300
C	-8.107800	-1.735600	-0.122600
N	-8.661100	-0.533600	-0.076600
C	-7.763300	0.465300	-0.017500
C	-6.361000	0.385500	-0.009400
C	-5.749300	-0.902900	-0.053500
N	-8.044400	1.814100	0.041700
C	-6.842600	2.486000	0.081700
N	-5.808000	1.663300	0.052600
O	-4.539900	-1.211000	-0.044100
N	-8.901600	-2.835000	-0.155300
H	-6.350900	-2.871300	-0.121900
N	3.160900	-2.565800	0.053500
C	3.029600	-3.932800	0.076300
N	1.895400	-4.591000	0.084800
C	0.821900	-3.765100	0.067900
C	0.828000	-2.363200	0.043900
C	2.084900	-1.701400	0.035800
N	-0.510200	-4.115200	0.068900
C	-1.238400	-2.944700	0.045700
N	-0.465500	-1.869400	0.029800
N	2.263800	-0.392200	0.013100
N	-3.160900	2.565800	0.053500
C	-3.029600	3.932800	0.076300
N	-1.895400	4.591000	0.084800
C	-0.821900	3.765100	0.067900
C	-0.828000	2.363200	0.043900
C	-2.084900	1.701400	0.035800
N	0.510200	4.115200	0.068900
C	1.238400	2.944700	0.045700
N	0.465500	1.869400	0.029800
N	-2.263800	0.392200	0.013100
N	6.731400	1.927800	-0.111300
C	8.107800	1.735600	-0.122600
N	8.661100	0.533600	-0.076600
C	7.763300	-0.465300	-0.017500
C	6.361000	-0.385500	-0.009400
C	5.749300	0.902900	-0.053500
N	8.044400	-1.814100	0.041700
C	6.842600	-2.486000	0.081700
N	5.808000	-1.663300	0.052600
O	4.539900	1.211000	-0.044100
N	8.901600	2.835000	-0.155300

H	-8.973500	2.222900	0.053500
H	-6.790800	3.564500	0.130500
H	6.790800	-3.564500	0.130500
H	8.973500	-2.222900	0.053500
H	3.965400	-4.483600	0.087800
H	-0.870600	-5.064300	0.084700
H	-2.321500	-2.927200	0.040600
H	1.452500	0.247300	0.010400
H	3.199500	0.052900	0.000300
H	-3.965400	4.483600	0.087800
H	0.870600	5.064300	0.084700
H	2.321500	2.927200	0.040600
H	-1.452500	-0.247300	0.010400
H	-3.199500	-0.052900	0.000300
H	6.350900	2.871300	-0.121900
H	-9.900100	-2.682700	-0.233200
H	-8.547200	-3.757900	-0.364200
H	9.900100	2.682700	-0.233200
H	8.547200	3.757900	-0.364200
H	4.151000	-2.170400	0.053000
H	-4.151000	2.170400	0.053000

(III')$_2$ gas phase C_{2h} E = -9467.8750

N	-6.731300	-1.928300	0.000000
C	-8.108000	-1.736500	0.000000
N	-8.661000	-0.533000	0.000000
C	-7.763000	0.466900	0.000000
C	-6.360500	0.387400	0.000000
C	-5.749000	-0.901600	0.000000
N	-8.043700	1.817200	0.000000
C	-6.841500	2.490000	0.000000
N	-5.807200	1.666600	0.000000
O	-4.539400	-1.209500	0.000000
N	-8.902200	-2.833400	0.000000
H	-6.349600	-2.871200	0.000000
N	3.159900	-2.567400	0.000000
C	3.028100	-3.934600	0.000000
N	1.893600	-4.592400	0.000000
C	0.820500	-3.765900	0.000000
C	0.827100	-2.363800	0.000000
C	2.084300	-1.702400	0.000000
N	-0.511800	-4.115500	0.000000
C	-1.239500	-2.944400	0.000000
N	-0.466200	-1.869400	0.000000
N	2.263500	-0.393000	0.000000
N	-3.159900	2.567400	0.000000
C	-3.028100	3.934600	0.000000
N	-1.893600	4.592400	0.000000
C	-0.820500	3.765900	0.000000

C	-0.827100	2.363800	0.000000
C	-2.084300	1.702400	0.000000
N	0.511800	4.115500	0.000000
C	1.239500	2.944400	0.000000
N	0.466200	1.869400	0.000000
N	-2.263500	0.393000	0.000000
N	6.731300	1.928300	0.000000
C	8.108000	1.736500	0.000000
N	8.661000	0.533000	0.000000
C	7.763000	-0.466900	0.000000
C	6.360500	-0.387400	0.000000
C	5.749000	0.901600	0.000000
N	8.043700	-1.817200	0.000000
C	6.841500	-2.490000	0.000000
N	5.807200	-1.666600	0.000000
O	4.539400	1.209500	0.000000
N	8.902200	2.833400	0.000000
H	-8.972600	2.226300	0.000000
H	-6.789500	3.569600	0.000000
H	6.789500	-3.569600	0.000000
H	8.972600	-2.226300	0.000000
H	3.963700	-4.485700	0.000000
H	-0.872400	-5.064600	0.000000
H	-2.322600	-2.926400	0.000000
H	1.452300	0.246400	0.000000
H	3.199400	0.052200	0.000000
H	-3.963700	4.485700	0.000000
H	0.872400	5.064600	0.000000
H	2.322600	2.926400	0.000000
H	-1.452300	-0.246400	0.000000
H	-3.199400	-0.052200	0.000000
H	6.349600	2.871200	0.000000
H	-9.904700	-2.691800	0.000000
H	-8.547100	-3.778400	0.000000
H	9.904700	2.691800	0.000000
H	8.547100	3.778400	0.000000
H	4.150400	-2.172700	0.000000
H	-4.150400	2.172700	0.000000

(III')$_2$ water C$_2$ E = -9605.3872

N	-6.696400	-1.947700	-0.068600
C	-8.065500	-1.754900	-0.076200
N	-8.623300	-0.546100	-0.045500
C	-7.717300	0.456800	-0.004000
C	-6.317200	0.360400	0.003800
C	-5.718400	-0.931700	-0.029700
N	-7.983500	1.806400	0.032500
C	-6.777500	2.466800	0.059500
N	-5.750000	1.632900	0.043600

O	-4.503200	-1.238800	-0.028000
N	-8.848400	-2.863800	-0.073200
H	-6.333900	-2.898300	-0.087900
N	3.121800	-2.599500	0.038300
C	2.990700	-3.959100	0.063100
N	1.848600	-4.615800	0.067700
C	0.773100	-3.783600	0.044400
C	0.794700	-2.381900	0.017200
C	2.052800	-1.731300	0.013600
N	-0.559100	-4.119700	0.043200
C	-1.275800	-2.948000	0.016300
N	-0.494000	-1.875300	-0.000500
N	2.242500	-0.422400	-0.010600
N	-3.121800	2.599500	0.038300
C	-2.990700	3.959100	0.063100
N	-1.848600	4.615800	0.067700
C	-0.773100	3.783600	0.044400
C	-0.794700	2.381900	0.017200
C	-2.052800	1.731300	0.013600
N	0.559100	4.119700	0.043200
C	1.275800	2.948000	0.016300
N	0.494000	1.875300	-0.000500
N	-2.242500	0.422400	-0.010600
N	6.696400	1.947700	-0.068600
C	8.065500	1.754900	-0.076200
N	8.623300	0.546100	-0.045500
C	7.717300	-0.456800	-0.004000
C	6.317200	-0.360400	0.003800
C	5.718400	0.931700	-0.029700
N	7.983500	-1.806400	0.032500
C	6.777500	-2.466800	0.059500
N	5.750000	-1.632900	0.043600
O	4.503200	1.238800	-0.028000
N	8.848400	2.863800	-0.073200
H	-8.902000	2.236700	0.036400
H	-6.714000	3.544200	0.088700
H	6.714000	-3.544200	0.088700
H	8.902000	-2.236700	0.036400
H	3.924600	-4.509500	0.080500
H	-0.943000	-5.058800	0.060400
H	-2.357000	-2.928900	0.010300
H	1.440900	0.228300	-0.016200
H	3.179500	0.011000	-0.009400
H	-3.924600	4.509500	0.080500
H	0.943000	5.058800	0.060400
H	2.357000	2.928900	0.010300
H	-1.440900	-0.228300	-0.016200
H	-3.179500	-0.011000	-0.009400
H	6.333900	2.898300	-0.087900
H	-9.836300	-2.729300	-0.254000

H	-8.459200	-3.761000	-0.335900
H	9.836300	2.729300	-0.254000
H	8.459200	3.761000	-0.335900
H	4.104900	-2.199300	0.040600
H	-4.104900	2.199300	0.040600

(III')$_2$ water C$_{2h}$ E = -9605.1932

N	-6.694300	-1.947500	0.000000
C	-8.064900	-1.756100	0.000000
N	-8.623200	-0.544900	0.000000
C	-7.717100	0.457600	0.000000
C	-6.316500	0.362300	0.000000
C	-5.717600	-0.929800	0.000000
N	-7.983400	1.808000	0.000000
C	-6.777300	2.469500	0.000000
N	-5.749600	1.636000	0.000000
O	-4.501900	-1.236400	0.000000
N	-8.844300	-2.858200	0.000000
H	-6.330300	-2.897700	0.000000
N	3.120800	-2.601800	0.000000
C	2.988900	-3.961500	0.000000
N	1.846400	-4.617500	0.000000
C	0.771400	-3.784500	0.000000
C	0.793800	-2.382500	0.000000
C	2.052200	-1.732600	0.000000
N	-0.561200	-4.119500	0.000000
C	-1.277100	-2.947000	0.000000
N	-0.494600	-1.874700	0.000000
N	2.242500	-0.423600	0.000000
N	-3.120800	2.601800	0.000000
C	-2.988900	3.961500	0.000000
N	-1.846400	4.617500	0.000000
C	-0.771400	3.784500	0.000000
C	-0.793800	2.382500	0.000000
C	-2.052200	1.732600	0.000000
N	0.561200	4.119500	0.000000
C	1.277100	2.947000	0.000000
N	0.494600	1.874700	0.000000
N	-2.242500	0.423600	0.000000
N	6.694300	1.947500	0.000000
C	8.064900	1.756100	0.000000
N	8.623200	0.544900	0.000000
C	7.717100	-0.457600	0.000000
C	6.316500	-0.362300	0.000000
C	5.717600	0.929800	0.000000
N	7.983400	-1.808000	0.000000
C	6.777300	-2.469500	0.000000
N	5.749600	-1.636000	0.000000
O	4.501900	1.236400	0.000000

N	8.844300	2.858200	0.000000
H	-8.901900	2.238200	0.000000
H	-6.714300	3.547400	0.000000
H	6.714300	-3.547400	0.000000
H	8.901900	-2.238200	0.000000
H	3.922600	-4.512700	0.000000
H	-0.945700	-5.058500	0.000000
H	-2.358400	-2.927000	0.000000
H	1.441000	0.227100	0.000000
H	3.179700	0.009800	0.000000
H	-3.922600	4.512700	0.000000
H	0.945700	5.058500	0.000000
H	2.358400	2.927000	0.000000
H	-1.441000	-0.227100	0.000000
H	-3.179700	-0.009800	0.000000
H	6.330300	2.897700	0.000000
H	-9.849700	-2.748900	0.000000
H	-8.457800	-3.792000	0.000000
H	9.849700	2.748900	0.000000
H	8.457800	3.792000	0.000000
H	4.104200	-2.202200	0.000000
H	-4.104200	2.202200	0.000000

$(Li \cdot I)_2$ gas phase C_2 E = -9475.9317

N	0.642200	-3.720800	-0.209600
C	1.205400	-4.899100	-0.575800
N	2.473400	-5.131300	-0.916500
C	3.209100	-4.011000	-0.871000
C	2.769200	-2.722800	-0.528600
C	1.399000	-2.591200	-0.192300
N	4.553800	-3.865300	-1.166100
C	4.870300	-2.545400	-1.000700
N	3.827800	-1.811000	-0.618000
N	0.810400	-1.422900	0.151200
N	-2.088200	-3.586600	0.518400
C	-2.707600	-4.785700	0.864300
N	-4.023300	-4.954500	0.953200
C	-4.717400	-3.838300	0.677900
C	-4.186900	-2.586600	0.367200
C	-2.791000	-2.415100	0.279500
N	-6.084600	-3.641100	0.653800
C	-6.311100	-2.314300	0.334600
N	-5.185600	-1.641300	0.153500
O	-2.227500	-1.303800	-0.002600
N	-1.880600	-5.845900	1.084900
N	-0.642200	3.720800	-0.209600
C	-1.205400	4.899100	-0.575800
N	-2.473400	5.131300	-0.916500
C	-3.209100	4.011000	-0.871000

C	-2.769200	2.722800	-0.528600
C	-1.399000	2.591200	-0.192300
N	-4.553800	3.865300	-1.166100
C	-4.870300	2.545400	-1.000700
N	-3.827800	1.811000	-0.618000
N	-0.810400	1.422900	0.151200
N	2.088200	3.586600	0.518400
C	2.707600	4.785700	0.864300
N	4.023300	4.954500	0.953200
C	4.717400	3.838300	0.677900
C	4.186900	2.586600	0.367200
C	2.791000	2.415100	0.279500
N	6.084600	3.641100	0.653800
C	6.311100	2.314300	0.334600
N	5.185600	1.641300	0.153500
O	2.227500	1.303800	-0.002600
N	1.880600	5.845900	1.084900
H	0.532100	-5.753300	-0.600100
H	5.179200	-4.610100	-1.458500
H	5.869800	-2.169600	-1.170900
H	1.323900	-0.553200	0.082700
H	-0.208300	-1.378100	0.172300
H	-1.051600	-3.604300	0.291000
H	-6.792300	-4.345700	0.839200
H	-7.309800	-1.909100	0.251000
H	-0.924000	-5.684300	1.374500
H	-2.323700	-6.689900	1.431400
H	-0.532100	5.753300	-0.600100
H	-5.179200	4.610100	-1.458500
H	-5.869800	2.169600	-1.170900
H	-1.323900	0.553200	0.082700
H	0.208300	1.378100	0.172300
H	1.051600	3.604300	0.291000
H	6.792300	4.345700	0.839200
H	7.309800	1.909100	0.251000
H	0.924000	5.684300	1.374500
H	2.323700	6.689900	1.431400
Li	3.880000	0.136400	-0.272400
Li	-3.880000	-0.136400	-0.272400

(Li • I)$_2$ gas phase C$_{2h}$ E = -9474.4857

N	0.700400	-3.841500	0.000000
C	1.381100	-5.013100	0.000000
N	2.697400	-5.214800	0.000000
C	3.368300	-4.053800	0.000000
C	2.817400	-2.764600	0.000000
C	1.401800	-2.674200	0.000000
N	4.739000	-3.862300	0.000000
C	4.963700	-2.513800	0.000000

N	3.836100	-1.806000	0.000000
N	0.729900	-1.503200	0.000000
N	-2.203500	-3.767100	0.000000
C	-2.895100	-4.980100	0.000000
N	-4.221900	-5.089100	0.000000
C	-4.849700	-3.905400	0.000000
C	-4.249100	-2.647000	0.000000
C	-2.844600	-2.534200	0.000000
N	-6.204600	-3.635300	0.000000
C	-6.357100	-2.260600	0.000000
N	-5.196200	-1.625800	0.000000
O	-2.222900	-1.417400	0.000000
N	-2.153700	-6.110800	0.000000
N	-0.700400	3.841500	0.000000
C	-1.381100	5.013100	0.000000
N	-2.697400	5.214800	0.000000
C	-3.368300	4.053800	0.000000
C	-2.817400	2.764600	0.000000
C	-1.401800	2.674200	0.000000
N	-4.739000	3.862300	0.000000
C	-4.963700	2.513800	0.000000
N	-3.836100	1.806000	0.000000
N	-0.729900	1.503200	0.000000
N	2.203500	3.767100	0.000000
C	2.895100	4.980100	0.000000
N	4.221900	5.089100	0.000000
C	4.849700	3.905400	0.000000
C	4.249100	2.647000	0.000000
C	2.844600	2.534200	0.000000
N	6.204600	3.635300	0.000000
C	6.357100	2.260600	0.000000
N	5.196200	1.625800	0.000000
O	2.222900	1.417400	0.000000
N	2.153700	6.110800	0.000000
H	0.777000	-5.918500	0.000000
H	5.439500	-4.597600	0.000000
H	5.961500	-2.097500	0.000000
H	1.230200	-0.625600	0.000000
H	-0.292300	-1.494000	0.000000
H	-1.149300	-3.779000	0.000000
H	-6.949800	-4.325400	0.000000
H	-7.332400	-1.794300	0.000000
H	-1.146300	-6.100400	0.000000
H	-2.638300	-6.999300	0.000000
H	-0.777000	5.918500	0.000000
H	-5.439500	4.597600	0.000000
H	-5.961500	2.097500	0.000000
H	-1.230200	0.625600	0.000000
H	0.292300	1.494000	0.000000
H	1.149300	3.779000	0.000000

H	6.949800	4.325400	0.000000
H	7.332400	1.794300	0.000000
H	1.146300	6.100400	0.000000
H	2.638300	6.999300	0.000000
Li	3.806600	0.159000	0.000000
Li	-3.806600	-0.159000	0.000000

(Li • I)$_2$ water C$_2$ E = -9627.3257

N	0.815800	-3.246300	-0.055300
C	1.501800	-4.141500	0.689200
N	2.824600	-4.214400	0.889600
C	3.481700	-3.239700	0.224500
C	2.906200	-2.257600	-0.596700
C	1.498000	-2.280000	-0.734300
N	4.836800	-2.989800	0.186700
C	5.030000	-1.906200	-0.629000
N	3.895300	-1.431600	-1.131100
N	0.816100	-1.417400	-1.515500
N	-1.993500	-2.778200	0.387200
C	-2.560200	-3.437900	1.464400
N	-3.868900	-3.470800	1.718100
C	-4.599500	-2.788200	0.807300
C	-4.111500	-2.075600	-0.289900
C	-2.721400	-2.036200	-0.540900
N	-5.963500	-2.616200	0.752600
C	-6.239100	-1.827400	-0.348500
N	-5.144500	-1.478800	-1.004700
O	-2.171600	-1.406900	-1.496500
N	-1.699600	-4.123600	2.272100
N	-0.815800	3.246300	-0.055300
C	-1.501800	4.141500	0.689200
N	-2.824600	4.214400	0.889600
C	-3.481700	3.239700	0.224500
C	-2.906200	2.257600	-0.596700
C	-1.498000	2.280000	-0.734300
N	-4.836800	2.989800	0.186700
C	-5.030000	1.906200	-0.629000
N	-3.895300	1.431600	-1.131100
N	-0.816100	1.417400	-1.515500
N	1.993500	2.778200	0.387200
C	2.560200	3.437900	1.464400
N	3.868900	3.470800	1.718100
C	4.599500	2.788200	0.807300
C	4.111500	2.075600	-0.289900
C	2.721400	2.036200	-0.540900
N	5.963500	2.616200	0.752600
C	6.239100	1.827400	-0.348500
N	5.144500	1.478800	-1.004700
O	2.171600	1.406900	-1.496500

N	1.699600	4.123600	2.272100
H	0.899300	-4.897500	1.186200
H	5.559500	-3.506600	0.675500
H	6.012600	-1.500200	-0.816300
H	1.285000	-0.576900	-1.831100
H	-0.206600	-1.444700	-1.531100
H	-0.967800	-2.916500	0.211400
H	-6.645400	-2.996700	1.399800
H	-7.248800	-1.545100	-0.607200
H	-0.738800	-3.807200	2.326500
H	-2.088800	-4.457900	3.147000
H	-0.899300	4.897500	1.186200
H	-5.559500	3.506600	0.675500
H	-6.012600	1.500200	-0.816300
H	-1.285000	0.576900	-1.831100
H	0.206600	1.444700	-1.531100
H	0.967800	2.916500	0.211400
H	6.645400	2.996700	1.399800
H	7.248800	1.545100	-0.607200
H	0.738800	3.807200	2.326500
H	2.088800	4.457900	3.147000
Li	3.912000	0.289500	-2.253700
Li	-3.912000	-0.289500	-2.253700

$(Li \bullet I)_2$ water C_{2h} E = -9621.4438

N	0.702800	-3.883400	0.000000
C	1.376800	-5.053000	0.000000
N	2.700200	-5.258400	0.000000
C	3.378900	-4.091200	0.000000
C	2.819400	-2.805600	0.000000
C	1.405800	-2.712900	0.000000
N	4.743400	-3.891800	0.000000
C	4.959600	-2.540600	0.000000
N	3.828100	-1.842100	0.000000
N	0.736600	-1.544700	0.000000
N	-2.205400	-3.807000	0.000000
C	-2.896000	-5.012100	0.000000
N	-4.229600	-5.112500	0.000000
C	-4.849500	-3.914200	0.000000
C	-4.237200	-2.661400	0.000000
C	-2.829400	-2.564000	0.000000
N	-6.198700	-3.637700	0.000000
C	-6.343700	-2.264000	0.000000
N	-5.179000	-1.635600	0.000000
O	-2.182900	-1.466900	0.000000
N	-2.155100	-6.141000	0.000000
N	-0.702800	3.883400	0.000000
C	-1.376800	5.053000	0.000000
N	-2.700200	5.258400	0.000000

C	-3.378900	4.091200	0.000000
C	-2.819400	2.805600	0.000000
C	-1.405800	2.712900	0.000000
N	-4.743400	3.891800	0.000000
C	-4.959600	2.540600	0.000000
N	-3.828100	1.842100	0.000000
N	-0.736600	1.544700	0.000000
N	2.205400	3.807000	0.000000
C	2.896000	5.012100	0.000000
N	4.229600	5.112500	0.000000
C	4.849500	3.914200	0.000000
C	4.237200	2.661400	0.000000
C	2.829400	2.564000	0.000000
N	6.198700	3.637700	0.000000
C	6.343700	2.264000	0.000000
N	5.179000	1.635600	0.000000
O	2.182900	1.466900	0.000000
N	2.155100	6.141000	0.000000
H	0.768200	-5.953400	0.000000
H	5.458200	-4.611200	0.000000
H	5.954500	-2.120900	0.000000
H	1.242700	-0.669300	0.000000
H	-0.287800	-1.531400	0.000000
H	-1.155700	-3.826200	0.000000
H	-6.953400	-4.315500	0.000000
H	-7.316100	-1.794600	0.000000
H	-1.147400	-6.124600	0.000000
H	-2.622700	-7.037400	0.000000
H	-0.768200	5.953400	0.000000
H	-5.458200	4.611200	0.000000
H	-5.954500	2.120900	0.000000
H	-1.242700	0.669300	0.000000
H	0.287800	1.531400	0.000000
H	1.155700	3.826200	0.000000
H	6.953400	4.315500	0.000000
H	7.316100	1.794600	0.000000
H	1.147400	6.124600	0.000000
H	2.622700	7.037400	0.000000
Li	3.780500	0.141000	0.000000
Li	-3.780500	-0.141000	0.000000

$(Li \bullet II)_2$ gas phase C_2 E = -9473.8889

N	-3.003100	-3.133800	-0.535800
C	-3.987000	-4.038500	-0.929600
N	-5.272100	-3.737300	-1.072300
C	-5.547200	-2.450400	-0.795800
C	-4.627800	-1.466500	-0.435800
C	-3.263200	-1.791300	-0.295200
N	-6.759900	-1.789200	-0.816300

C	-6.523900	-0.470400	-0.471200
N	-5.243200	-0.235400	-0.233300
O	-2.361300	-0.952500	0.031300
N	-3.568800	-5.322900	-1.137100
H	-7.660500	-2.199500	-1.044800
H	-2.060300	-3.519900	-0.265500
N	3.003100	3.133800	-0.535800
C	3.987000	4.038500	-0.929600
N	5.272100	3.737300	-1.072300
C	5.547200	2.450400	-0.795800
C	4.627800	1.466500	-0.435800
C	3.263200	1.791300	-0.295200
N	6.759900	1.789200	-0.816300
C	6.523900	0.470400	-0.471200
N	5.243200	0.235400	-0.233300
O	2.361300	0.952500	0.031300
N	3.568800	5.322900	-1.137100
H	7.660500	2.199500	-1.044800
H	2.060300	3.519900	-0.265500
N	-2.617400	2.489600	0.496600
C	-3.319100	3.572000	0.946900
N	-2.864700	4.790500	1.221100
C	-1.548300	4.899400	0.978100
C	-0.700900	3.895400	0.485200
C	-1.278500	2.624300	0.261300
N	-0.734200	6.004000	1.147400
C	0.532200	5.640700	0.757000
N	0.597100	4.381600	0.343000
N	-0.571300	1.560000	-0.182700
H	-1.028700	6.909200	1.501000
Li	-3.541300	0.739600	0.288800
N	2.617400	-2.489600	0.496600
C	3.319100	-3.572000	0.946900
N	2.864700	-4.790500	1.221100
C	1.548300	-4.899400	0.978100
C	0.700900	-3.895400	0.485200
C	1.278500	-2.624300	0.261300
N	0.734200	-6.004000	1.147400
C	-0.532200	-5.640700	0.757000
N	-0.597100	-4.381600	0.343000
N	0.571300	-1.560000	-0.182700
H	1.028700	-6.909200	1.501000
Li	3.541300	-0.739600	0.288800
H	-1.041900	0.663400	-0.228200
H	0.447200	1.553400	-0.125900
H	-0.447200	-1.553400	-0.125900
H	1.041900	-0.663400	-0.228200
H	-4.263300	-5.943600	-1.540200
H	-2.609600	-5.482900	-1.422900
H	2.609600	5.482900	-1.422900

H	4.263300	5.943600	-1.540200
H	1.368900	6.325200	0.801200
H	-1.368900	-6.325200	0.801200
H	-7.320600	0.258100	-0.411900
H	7.320600	-0.258100	-0.411900
H	-4.382100	3.407400	1.107800
H	4.382100	-3.407400	1.107800

(Li • II)$_2$ gas phase C_{2h} E = -9470.7599

N	-3.182900	-3.238600	0.000000
C	-4.252300	-4.138500	0.000000
N	-5.533100	-3.784500	0.000000
C	-5.716200	-2.455500	0.000000
C	-4.718600	-1.482000	0.000000
C	-3.359500	-1.857100	0.000000
N	-6.894900	-1.735700	0.000000
C	-6.564800	-0.392300	0.000000
N	-5.256300	-0.197600	0.000000
O	-2.393700	-1.026800	0.000000
N	-3.940000	-5.455200	0.000000
H	-7.832700	-2.125900	0.000000
H	-2.212100	-3.622600	0.000000
N	3.182900	3.238600	0.000000
C	4.252300	4.138500	0.000000
N	5.533100	3.784500	0.000000
C	5.716200	2.455500	0.000000
C	4.718600	1.482000	0.000000
C	3.359500	1.857100	0.000000
N	6.894900	1.735700	0.000000
C	6.564800	0.392300	0.000000
N	5.256300	0.197600	0.000000
O	2.393700	1.026800	0.000000
N	3.940000	5.455200	0.000000
H	7.832700	2.125900	0.000000
H	2.212100	3.622600	0.000000
N	-2.619400	2.521700	0.000000
C	-3.432300	3.616600	0.000000
N	-3.080100	4.897000	0.000000
C	-1.745500	5.045300	0.000000
C	-0.775700	4.027100	0.000000
C	-1.263900	2.697400	0.000000
N	-1.029100	6.224500	0.000000
C	0.301500	5.887600	0.000000
N	0.509300	4.577500	0.000000
N	-0.475100	1.603500	0.000000
H	-1.425900	7.159200	0.000000
Li	-3.476100	0.741900	0.000000
N	2.619400	-2.521700	0.000000
C	3.432300	-3.616600	0.000000

N	3.080100	-4.897000	0.000000
C	1.745500	-5.045300	0.000000
C	0.775700	-4.027100	0.000000
C	1.263900	-2.697400	0.000000
N	1.029100	-6.224500	0.000000
C	-0.301500	-5.887600	0.000000
N	-0.509300	-4.577500	0.000000
N	0.475100	-1.603500	0.000000
H	1.425900	-7.159200	0.000000
Li	3.476100	-0.741900	0.000000
H	-0.917300	0.694000	0.000000
H	0.544000	1.646600	0.000000
H	-0.544000	-1.646600	0.000000
H	0.917300	-0.694000	0.000000
H	-4.696200	-6.127500	0.000000
H	-2.986700	-5.777000	0.000000
H	2.986700	5.777000	0.000000
H	4.696200	6.127500	0.000000
H	1.063800	6.655500	0.000000
H	-1.063800	-6.655500	0.000000
H	-7.318300	0.383000	0.000000
H	7.318300	-0.383000	0.000000
H	-4.499900	3.408200	0.000000
H	4.499900	-3.408200	0.000000

(Li • II)$_2$ water C$_2$ E = -9627.9319

N	-2.290000	-2.697200	-0.633200
C	-2.929300	-3.158700	-1.764700
N	-4.009000	-2.579800	-2.292600
C	-4.429800	-1.509100	-1.582300
C	-3.851000	-0.971400	-0.425400
C	-2.675200	-1.569600	0.096500
N	-5.517200	-0.704500	-1.839100
C	-5.567600	0.254900	-0.853100
N	-4.580000	0.128800	0.020800
O	-1.996800	-1.187700	1.091800
N	-2.427800	-4.295900	-2.324300
H	-6.167800	-0.800700	-2.611200
H	-1.499900	-3.266000	-0.239700
N	2.290000	2.697200	-0.633200
C	2.929300	3.158700	-1.764700
N	4.009000	2.579800	-2.292600
C	4.429800	1.509100	-1.582300
C	3.851000	0.971400	-0.425400
C	2.675200	1.569600	0.096500
N	5.517200	0.704500	-1.839100
C	5.567600	-0.254900	-0.853100
N	4.580000	-0.128800	0.020800
O	1.996800	1.187700	1.091800

N	2.427800	4.295900	-2.324300
H	6.167800	0.800700	-2.611200
H	1.499900	3.266000	-0.239700
N	-2.954900	2.670500	1.452000
C	-3.635400	3.840300	1.532600
N	-3.186700	5.083200	1.317300
C	-1.883500	5.084600	0.958700
C	-1.060500	3.957700	0.822600
C	-1.631200	2.691800	1.105100
N	-1.083700	6.158400	0.626700
C	0.152000	5.661500	0.299500
N	0.210900	4.339300	0.401800
N	-0.940400	1.540600	1.034000
H	-1.356700	7.135200	0.618500
Li	-4.097800	1.024600	1.742000
N	2.954900	-2.670500	1.452000
C	3.635400	-3.840300	1.532600
N	3.186700	-5.083200	1.317300
C	1.883500	-5.084600	0.958700
C	1.060500	-3.957700	0.822600
C	1.631200	-2.691800	1.105100
N	1.083700	-6.158400	0.626700
C	-0.152000	-5.661500	0.299500
N	-0.210900	-4.339300	0.401800
N	0.940400	-1.540600	1.034000
H	1.356700	-7.135200	0.618500
Li	4.097800	-1.024600	1.742000
H	-1.402400	0.642400	1.190200
H	0.079300	1.545600	0.959400
H	-0.079300	-1.545600	0.959400
H	1.402400	-0.642400	1.190200
H	-2.770300	-4.533100	-3.248300
H	-1.466200	-4.552500	-2.132300
H	1.466200	4.552500	-2.132300
H	2.770300	4.533100	-3.248300
H	0.966700	6.301700	-0.006100
H	-0.966700	-6.301700	-0.006100
H	-6.339900	1.008900	-0.827600
H	6.339900	-1.008900	-0.827600
H	-4.682100	3.751400	1.813000
H	4.682100	-3.751400	1.813000

(Li • II)$_2$ water C$_{2h}$ E = -9619.2081

N	-3.191700	-3.261800	0.000000
C	-4.247800	-4.166300	0.000000
N	-5.537000	-3.814400	0.000000
C	-5.720600	-2.477300	0.000000
C	-4.724000	-1.502500	0.000000
C	-3.363400	-1.880400	0.000000

N	-6.899800	-1.765600	0.000000
C	-6.576800	-0.422900	0.000000
N	-5.268900	-0.220800	0.000000
O	-2.389000	-1.064500	0.000000
N	-3.924400	-5.476800	0.000000
H	-7.838000	-2.151300	0.000000
H	-2.220400	-3.641800	0.000000
N	3.191700	3.261800	0.000000
C	4.247800	4.166300	0.000000
N	5.537000	3.814400	0.000000
C	5.720600	2.477300	0.000000
C	4.724000	1.502500	0.000000
C	3.363400	1.880400	0.000000
N	6.899800	1.765600	0.000000
C	6.576800	0.422900	0.000000
N	5.268900	0.220800	0.000000
O	2.389000	1.064500	0.000000
N	3.924400	5.476800	0.000000
H	7.838000	2.151300	0.000000
H	2.220400	3.641800	0.000000
N	-2.613000	2.550000	0.000000
C	-3.415600	3.640300	0.000000
N	-3.057300	4.929200	0.000000
C	-1.714000	5.071700	0.000000
C	-0.759400	4.040300	0.000000
C	-1.255400	2.714400	0.000000
N	-0.983300	6.238600	0.000000
C	0.341400	5.888200	0.000000
N	0.531000	4.574100	0.000000
N	-0.476800	1.617500	0.000000
H	-1.353400	7.182900	0.000000
Li	-3.471700	0.740400	0.000000
N	2.613000	-2.550000	0.000000
C	3.415600	-3.640300	0.000000
N	3.057300	-4.929200	0.000000
C	1.714000	-5.071700	0.000000
C	0.759400	-4.040300	0.000000
C	1.255400	-2.714400	0.000000
N	0.983300	-6.238600	0.000000
C	-0.341400	-5.888200	0.000000
N	-0.531000	-4.574100	0.000000
N	0.476800	-1.617500	0.000000
H	1.353400	-7.182900	0.000000
Li	3.471700	-0.740400	0.000000
H	-0.926200	0.710600	0.000000
H	0.544100	1.656900	0.000000
H	-0.544100	-1.656900	0.000000
H	0.926200	-0.710600	0.000000
H	-4.661100	-6.169100	0.000000
H	-2.967300	-5.788500	0.000000

H	2.967300	5.788500	0.000000
H	4.661100	6.169100	0.000000
H	1.112000	6.645400	0.000000
H	-1.112000	-6.645400	0.000000
H	-7.334900	0.345900	0.000000
H	7.334900	-0.345900	0.000000
H	-4.483000	3.435400	0.000000
H	4.483000	-3.435400	0.000000

(Na • I)$_2$ gas phase C$_2$ E = -9432.1761

N	0.535500	-3.749800	-0.624700
C	0.936600	-4.941400	-1.130200
N	2.112400	-5.239200	-1.684300
C	2.937500	-4.182400	-1.680700
C	2.668700	-2.892400	-1.187600
C	1.367900	-2.676700	-0.667500
N	4.232700	-4.121200	-2.161000
C	4.691300	-2.849400	-1.940000
N	3.788200	-2.069800	-1.353500
N	0.899900	-1.491600	-0.198300
N	-1.924100	-3.575300	0.725200
C	-2.367000	-4.731700	1.348700
N	-3.601200	-4.905100	1.805300
C	-4.393400	-3.842700	1.582400
C	-4.041700	-2.624800	0.985700
C	-2.709200	-2.439500	0.528600
N	-5.728900	-3.702200	1.898500
C	-6.125700	-2.445800	1.482700
N	-5.138200	-1.764500	0.927700
O	-2.231500	-1.395200	-0.015500
N	-1.465200	-5.750900	1.447200
N	-0.535500	3.749800	-0.624700
C	-0.936600	4.941400	-1.130200
N	-2.112400	5.239200	-1.684300
C	-2.937500	4.182400	-1.680700
C	-2.668700	2.892400	-1.187600
C	-1.367900	2.676700	-0.667500
N	-4.232700	4.121200	-2.161000
C	-4.691300	2.849400	-1.940000
N	-3.788200	2.069800	-1.353500
N	-0.899900	1.491600	-0.198300
N	1.924100	3.575300	0.725200
C	2.367000	4.731700	1.348700
N	3.601200	4.905100	1.805300
C	4.393400	3.842700	1.582400
C	4.041700	2.624800	0.985700
C	2.709200	2.439500	0.528600
N	5.728900	3.702200	1.898500
C	6.125700	2.445800	1.482700

N	5.138200	1.764500	0.927700
O	2.231500	1.395200	-0.015500
N	1.465200	5.750900	1.447200
H	0.203100	-5.743000	-1.076500
H	4.737500	-4.886400	-2.597400
H	5.690100	-2.546600	-2.224400
H	1.424200	-0.634200	-0.322300
H	-0.106600	-1.406300	-0.049900
H	-0.984800	-3.597400	0.239200
H	-6.307400	-4.403000	2.351000
H	-7.139700	-2.093900	1.611900
H	-0.473500	-5.548600	1.426000
H	-1.756600	-6.551000	1.997500
H	-0.203100	5.743000	-1.076500
H	-4.737500	4.886400	-2.597400
H	-5.690100	2.546600	-2.224400
H	-1.424200	0.634200	-0.322300
H	0.106600	1.406300	-0.049900
H	0.984800	3.597400	0.239200
H	6.307400	4.403000	2.351000
H	7.139700	2.093900	1.611900
H	0.473500	5.548600	1.426000
H	1.756600	6.551000	1.997500
Na	4.204600	0.029300	-0.360300
Na	-4.204600	-0.029300	-0.360300

$(Na \bullet I)_2$ gas phase C_{2h} E = -9428.4475

N	0.608800	-4.223700	0.000000
C	1.278600	-5.399700	0.000000
N	2.593300	-5.611900	0.000000
C	3.271300	-4.455600	0.000000
C	2.732700	-3.158600	0.000000
C	1.315800	-3.060200	0.000000
N	4.642500	-4.280300	0.000000
C	4.879700	-2.932100	0.000000
N	3.762000	-2.210400	0.000000
N	0.636400	-1.890100	0.000000
N	-2.304800	-4.188800	0.000000
C	-2.989500	-5.399000	0.000000
N	-4.314300	-5.504500	0.000000
C	-4.936900	-4.317000	0.000000
C	-4.348000	-3.047000	0.000000
C	-2.933300	-2.941000	0.000000
N	-6.294600	-4.071800	0.000000
C	-6.469300	-2.700800	0.000000
N	-5.321400	-2.046400	0.000000
O	-2.265200	-1.859400	0.000000
N	-2.248800	-6.532800	0.000000
N	-0.608800	4.223700	0.000000

C	-1.278600	5.399700	0.000000
N	-2.593300	5.611900	0.000000
C	-3.271300	4.455600	0.000000
C	-2.732700	3.158600	0.000000
C	-1.315800	3.060200	0.000000
N	-4.642500	4.280300	0.000000
C	-4.879700	2.932100	0.000000
N	-3.762000	2.210400	0.000000
N	-0.636400	1.890100	0.000000
N	2.304800	4.188800	0.000000
C	2.989500	5.399000	0.000000
N	4.314300	5.504500	0.000000
C	4.936900	4.317000	0.000000
C	4.348000	3.047000	0.000000
C	2.933300	2.941000	0.000000
N	6.294600	4.071800	0.000000
C	6.469300	2.700800	0.000000
N	5.321400	2.046400	0.000000
O	2.265200	1.859400	0.000000
N	2.248800	6.532800	0.000000
H	0.667000	-6.299800	0.000000
H	5.333800	-5.023800	0.000000
H	5.885300	-2.532900	0.000000
H	1.132700	-1.012200	0.000000
H	-0.391200	-1.882500	0.000000
H	-1.254500	-4.192200	0.000000
H	-7.024100	-4.777800	0.000000
H	-7.452100	-2.250600	0.000000
H	-1.241700	-6.524200	0.000000
H	-2.735000	-7.420000	0.000000
H	-0.667000	6.299800	0.000000
H	-5.333800	5.023800	0.000000
H	-5.885300	2.532900	0.000000
H	-1.132700	1.012200	0.000000
H	0.391200	1.882500	0.000000
H	1.254500	4.192200	0.000000
H	7.024100	4.777800	0.000000
H	7.452100	2.250600	0.000000
H	1.241700	6.524200	0.000000
H	2.735000	7.420000	0.000000
Na	3.913800	0.150600	0.000000
Na	-3.913800	-0.150600	0.000000

$(Na \bullet I)_2$ water C_2 E = -9581.6584

N	0.579900	-3.219500	-0.764400
C	1.052100	-4.027200	-1.739300
N	2.293400	-4.081000	-2.243600
C	3.117700	-3.208200	-1.624700
C	2.766600	-2.322300	-0.593600

C	1.413800	-2.313000	-0.182000
N	4.462300	-2.992500	-1.839800
C	4.868200	-2.030000	-0.949100
N	3.880800	-1.596700	-0.173900
N	0.927000	-1.472800	0.763200
N	-2.128400	-3.427300	0.132800
C	-2.787800	-4.588100	-0.210200
N	-4.099800	-4.766400	-0.067700
C	-4.719900	-3.685800	0.460200
C	-4.134700	-2.482200	0.876200
C	-2.731500	-2.307600	0.720200
N	-6.060400	-3.540800	0.731700
C	-6.237200	-2.291800	1.289400
N	-5.101100	-1.621700	1.395300
O	-2.043300	-1.301000	1.047300
N	-2.024900	-5.577000	-0.769600
N	-0.579900	3.219500	-0.764400
C	-1.052100	4.027200	-1.739300
N	-2.293400	4.081000	-2.243600
C	-3.117700	3.208200	-1.624700
C	-2.766600	2.322300	-0.593600
C	-1.413800	2.313000	-0.182000
N	-4.462300	2.992500	-1.839800
C	-4.868200	2.030000	-0.949100
N	-3.880800	1.596700	-0.173900
N	-0.927000	1.472800	0.763200
N	2.128400	3.427300	0.132800
C	2.787800	4.588100	-0.210200
N	4.099800	4.766400	-0.067700
C	4.719900	3.685800	0.460200
C	4.134700	2.482200	0.876200
C	2.731500	2.307600	0.720200
N	6.060400	3.540800	0.731700
C	6.237200	2.291800	1.289400
N	5.101100	1.621700	1.395300
O	2.043300	1.301000	1.047300
N	2.024900	5.577000	-0.769600
H	0.326100	-4.708200	-2.176000
H	5.047600	-3.461200	-2.522500
H	5.892600	-1.689700	-0.906800
H	1.434600	-0.607600	0.936900
H	-0.089100	-1.433900	0.891200
H	-1.117700	-3.340800	-0.142000
H	-6.785600	-4.229100	0.562000
H	-7.210600	-1.936300	1.593000
H	-1.047500	-5.625200	-0.504700
H	-2.479600	-6.479700	-0.856600
H	-0.326100	4.708200	-2.176000
H	-5.047600	3.461200	-2.522500
H	-5.892600	1.689700	-0.906800

H	-1.434600	0.607600	0.936900
H	0.089100	1.433900	0.891200
H	1.117700	3.340800	-0.142000
H	6.785600	4.229100	0.562000
H	7.210600	1.936300	1.593000
H	1.047500	5.625200	-0.504700
H	2.479600	6.479700	-0.856600
Na	4.180200	-0.508700	1.965800
Na	-4.180200	0.508700	1.965800

(Na • I)$_2$ water C_{2h} E = -9573.8613

N	0.591600	-4.307200	0.000000
C	1.266300	-5.475500	0.000000
N	2.590000	-5.679000	0.000000
C	3.266700	-4.510400	0.000000
C	2.707600	-3.223000	0.000000
C	1.292400	-3.134200	0.000000
N	4.630300	-4.313100	0.000000
C	4.845900	-2.959900	0.000000
N	3.716600	-2.258600	0.000000
N	0.613600	-1.971100	0.000000
N	-2.325300	-4.276300	0.000000
C	-3.011600	-5.478700	0.000000
N	-4.344200	-5.575100	0.000000
C	-4.959800	-4.372900	0.000000
C	-4.355800	-3.111300	0.000000
C	-2.940200	-3.020700	0.000000
N	-6.311400	-4.113900	0.000000
C	-6.471100	-2.741800	0.000000
N	-5.315200	-2.099000	0.000000
O	-2.260000	-1.952400	0.000000
N	-2.271700	-6.610200	0.000000
N	-0.591600	4.307200	0.000000
C	-1.266300	5.475500	0.000000
N	-2.590000	5.679000	0.000000
C	-3.266700	4.510400	0.000000
C	-2.707600	3.223000	0.000000
C	-1.292400	3.134200	0.000000
N	-4.630300	4.313100	0.000000
C	-4.845900	2.959900	0.000000
N	-3.716600	2.258600	0.000000
N	-0.613600	1.971100	0.000000
N	2.325300	4.276300	0.000000
C	3.011600	5.478700	0.000000
N	4.344200	5.575100	0.000000
C	4.959800	4.372900	0.000000
C	4.355800	3.111300	0.000000
C	2.940200	3.020700	0.000000
N	6.311400	4.113900	0.000000

C	6.471100	2.741800	0.000000
N	5.315200	2.099000	0.000000
O	2.260000	1.952400	0.000000
N	2.271700	6.610200	0.000000
H	0.658400	-6.376400	0.000000
H	5.344000	-5.033500	0.000000
H	5.843000	-2.544100	0.000000
H	1.114000	-1.094200	0.000000
H	-0.414300	-1.960500	0.000000
H	-1.277000	-4.288200	0.000000
H	-7.055700	-4.802900	0.000000
H	-7.449200	-2.284000	0.000000
H	-1.264000	-6.594800	0.000000
H	-2.740300	-7.506000	0.000000
H	-0.658400	6.376400	0.000000
H	-5.344000	5.033500	0.000000
H	-5.843000	2.544100	0.000000
H	-1.114000	1.094200	0.000000
H	0.414300	1.960500	0.000000
H	1.277000	4.288200	0.000000
H	7.055700	4.802900	0.000000
H	7.449200	2.284000	0.000000
H	1.264000	6.594800	0.000000
H	2.740300	7.506000	0.000000
Na	3.891000	0.128500	0.000000
Na	-3.891000	-0.128500	0.000000

(Na • II)$_2$ gas phase C$_2$ E = -9431.0478

N	-2.968100	-3.235400	-0.643100
C	-3.800100	-4.213400	-1.166700
N	-4.998000	-3.974300	-1.680200
C	-5.340800	-2.675600	-1.625400
C	-4.570200	-1.610100	-1.144400
C	-3.274600	-1.873200	-0.621600
N	-6.520800	-2.094300	-2.038600
C	-6.427400	-0.738900	-1.787800
N	-5.267500	-0.407700	-1.248600
O	-2.456300	-1.022100	-0.159400
N	-3.342200	-5.500300	-1.094700
H	-7.309900	-2.582200	-2.451500
H	-2.132100	-3.553500	-0.091600
N	2.968100	3.235400	-0.643100
C	3.800100	4.213400	-1.166700
N	4.998000	3.974300	-1.680200
C	5.340800	2.675600	-1.625400
C	4.570200	1.610100	-1.144400
C	3.274600	1.873200	-0.621600
N	6.520800	2.094300	-2.038600
C	6.427400	0.738900	-1.787800

N	5.267500	0.407700	-1.248600
O	2.456300	1.022100	-0.159400
N	3.342200	5.500300	-1.094700
H	7.309900	2.582200	-2.451500
H	2.132100	3.553500	-0.091600
N	-2.461700	2.654000	0.976600
C	-3.046000	3.675800	1.663900
N	-2.480800	4.787100	2.128800
C	-1.179600	4.850600	1.810600
C	-0.444700	3.895800	1.089900
C	-1.128800	2.720100	0.703200
N	-0.273800	5.852900	2.105100
C	0.932000	5.486400	1.556600
N	0.871400	4.318200	0.932400
N	-0.524500	1.684600	0.063900
H	-0.475200	6.697200	2.632300
Na	-3.827600	1.010900	-0.050800
N	2.461700	-2.654000	0.976600
C	3.046000	-3.675800	1.663900
N	2.480800	-4.787100	2.128800
C	1.179600	-4.850600	1.810600
C	0.444700	-3.895800	1.089900
C	1.128800	-2.720100	0.703200
N	0.273800	-5.852900	2.105100
C	-0.932000	-5.486400	1.556600
N	-0.871400	-4.318200	0.932400
N	0.524500	-1.684600	0.063900
H	0.475200	-6.697200	2.632300
Na	3.827600	-1.010900	-0.050800
H	-1.035200	0.811800	-0.023400
H	0.495300	1.622600	0.028200
H	-0.495300	-1.622600	0.028200
H	1.035200	-0.811800	-0.023400
H	-3.896200	-6.184600	-1.599400
H	-2.342200	-5.662800	-1.070900
H	2.342200	5.662800	-1.070900
H	3.896200	6.184600	-1.599400
H	1.818000	6.102300	1.640000
H	-1.818000	-6.102300	1.640000
H	-7.234900	-0.056400	-2.016200
H	7.234900	0.056400	-2.016200
H	-4.110200	3.562300	1.864000
H	4.110200	-3.562300	1.864000

(Na • II)$_2$ gas phase C_{2h} E = -9426.7205

N	-3.442800	-3.609200	0.000000
C	-4.504100	-4.508700	0.000000
N	-5.782600	-4.153300	0.000000
C	-5.960300	-2.822800	0.000000

C	-4.969800	-1.834400	0.000000
C	-3.604100	-2.219900	0.000000
N	-7.150000	-2.125100	0.000000
C	-6.841200	-0.777500	0.000000
N	-5.538000	-0.560200	0.000000
O	-2.607200	-1.435800	0.000000
N	-4.194400	-5.828300	0.000000
H	-8.078700	-2.535100	0.000000
H	-2.468100	-3.981300	0.000000
N	3.442800	3.609200	0.000000
C	4.504100	4.508700	0.000000
N	5.782600	4.153300	0.000000
C	5.960300	2.822800	0.000000
C	4.969800	1.834400	0.000000
C	3.604100	2.219900	0.000000
N	7.150000	2.125100	0.000000
C	6.841200	0.777500	0.000000
N	5.538000	0.560200	0.000000
O	2.607200	1.435800	0.000000
N	4.194400	5.828300	0.000000
H	8.078700	2.535100	0.000000
H	2.468100	3.981300	0.000000
N	-2.371300	2.796500	0.000000
C	-3.182400	3.890300	0.000000
N	-2.836100	5.173400	0.000000
C	-1.502900	5.323600	0.000000
C	-0.530700	4.306600	0.000000
C	-1.017700	2.974000	0.000000
N	-0.790900	6.505400	0.000000
C	0.540500	6.172200	0.000000
N	0.751500	4.863600	0.000000
N	-0.220500	1.882600	0.000000
H	-1.191300	7.438300	0.000000
Na	-3.585000	0.753400	0.000000
N	2.371300	-2.796500	0.000000
C	3.182400	-3.890300	0.000000
N	2.836100	-5.173400	0.000000
C	1.502900	-5.323600	0.000000
C	0.530700	-4.306600	0.000000
C	1.017700	-2.974000	0.000000
N	0.790900	-6.505400	0.000000
C	-0.540500	-6.172200	0.000000
N	-0.751500	-4.863600	0.000000
N	0.220500	-1.882600	0.000000
H	1.191300	-7.438300	0.000000
Na	3.585000	-0.753400	0.000000
H	-0.658300	0.971800	0.000000
H	0.804300	1.923900	0.000000
H	-0.804300	-1.923900	0.000000
H	0.658300	-0.971800	0.000000

H	-4.952500	-6.498100	0.000000
H	-3.242900	-6.154100	0.000000
H	3.242900	6.154100	0.000000
H	4.952500	6.498100	0.000000
H	1.300900	6.941700	0.000000
H	-1.300900	-6.941700	0.000000
H	-7.607500	-0.014900	0.000000
H	7.607500	0.014900	0.000000
H	-4.252400	3.688200	0.000000
H	4.252400	-3.688200	0.000000

(Na • II)$_2$ water C$_2$ E = -9585.6708

N	-2.144000	-1.303300	0.885700
C	-2.768200	-1.139200	2.106900
N	-4.038700	-0.768600	2.249600
C	-4.672500	-0.583300	1.068300
C	-4.141800	-0.771000	-0.211000
C	-2.778400	-1.141500	-0.348800
N	-5.967800	-0.175000	0.854800
C	-6.165300	-0.123300	-0.511300
N	-5.085700	-0.474300	-1.191100
O	-2.153900	-1.286400	-1.435000
N	-1.989400	-1.323500	3.217900
H	-6.656500	0.042300	1.567400
H	-1.114400	-1.501500	0.861300
N	2.144000	1.303300	0.885700
C	2.768200	1.139200	2.106900
N	4.038700	0.768600	2.249600
C	4.672500	0.583300	1.068300
C	4.141800	0.771000	-0.211000
C	2.778400	1.141500	-0.348800
N	5.967800	0.175000	0.854800
C	6.165300	0.123300	-0.511300
N	5.085700	0.474300	-1.191100
O	2.153900	1.286400	-1.435000
N	1.989400	1.323500	3.217900
H	6.656500	-0.042300	1.567400
H	1.114400	1.501500	0.861300
N	-2.834800	2.124500	-2.137700
C	-3.917800	2.536700	-1.439300
N	-4.032300	2.733200	-0.118500
C	-2.880500	2.449200	0.525100
C	-1.663300	2.064000	-0.063400
C	-1.654900	1.922200	-1.474800
N	-2.640000	2.454400	1.882500
C	-1.332400	2.081100	2.066500
N	-0.701600	1.844600	0.924500
N	-0.570800	1.571700	-2.186200
H	-3.309200	2.675900	2.611700

Na	-3.579600	-0.195100	-3.155800
N	2.834800	-2.124500	-2.137700
C	3.917800	-2.536700	-1.439300
N	4.032300	-2.733200	-0.118500
C	2.880500	-2.449200	0.525100
C	1.663300	-2.064000	-0.063400
C	1.654900	-1.922200	-1.474800
N	2.640000	-2.454400	1.882500
C	1.332400	-2.081100	2.066500
N	0.701600	-1.844600	0.924500
N	0.570800	-1.571700	-2.186200
H	3.309200	-2.675900	2.611700
Na	3.579600	0.195100	-3.155800
H	-0.644300	1.536800	-3.195900
H	0.354400	1.436700	-1.762600
H	-0.354400	-1.436700	-1.762600
H	0.644300	-1.536800	-3.195900
H	-2.502400	-1.362900	4.093100
H	-1.245200	-2.008900	3.153900
H	1.245200	2.008900	3.153900
H	2.502400	1.362900	4.093100
H	-0.911900	1.983300	3.056400
H	0.911900	-1.983300	3.056400
H	-7.111200	0.179100	-0.936600
H	7.111200	-0.179100	-0.936600
H	-4.813200	2.713800	-2.030100
H	4.813200	-2.713800	-2.030100

(Na • II)$_2$ water C$_{2h}$ E = -9572.8853

N	-3.477000	-3.675900	0.000000
C	-4.533900	-4.570800	0.000000
N	-5.817900	-4.205800	0.000000
C	-5.986600	-2.866300	0.000000
C	-4.987600	-1.888300	0.000000
C	-3.626000	-2.286900	0.000000
N	-7.168100	-2.161400	0.000000
C	-6.849300	-0.817600	0.000000
N	-5.543700	-0.610100	0.000000
O	-2.622100	-1.516800	0.000000
N	-4.224900	-5.886600	0.000000
H	-8.103500	-2.554000	0.000000
H	-2.501600	-4.049000	0.000000
N	3.477000	3.675900	0.000000
C	4.533900	4.570800	0.000000
N	5.817900	4.205800	0.000000
C	5.986600	2.866300	0.000000
C	4.987600	1.888300	0.000000
C	3.626000	2.286900	0.000000
N	7.168100	2.161400	0.000000

C	6.849300	0.817600	0.000000
N	5.543700	0.610100	0.000000
O	2.622100	1.516800	0.000000
N	4.224900	5.886600	0.000000
H	8.103500	2.554000	0.000000
H	2.501600	4.049000	0.000000
N	-2.348600	2.835900	0.000000
C	-3.153800	3.922400	0.000000
N	-2.803100	5.214600	0.000000
C	-1.461900	5.363400	0.000000
C	-0.502800	4.336800	0.000000
C	-0.993600	3.007200	0.000000
N	-0.738600	6.534700	0.000000
C	0.587800	6.192000	0.000000
N	0.783800	4.879700	0.000000
N	-0.202400	1.917900	0.000000
H	-1.115100	7.476700	0.000000
Na	-3.556100	0.749200	0.000000
N	2.348600	-2.835900	0.000000
C	3.153800	-3.922400	0.000000
N	2.803100	-5.214600	0.000000
C	1.461900	-5.363400	0.000000
C	0.502800	-4.336800	0.000000
C	0.993600	-3.007200	0.000000
N	0.738600	-6.534700	0.000000
C	-0.587800	-6.192000	0.000000
N	-0.783800	-4.879700	0.000000
N	0.202400	-1.917900	0.000000
H	1.115100	-7.476700	0.000000
Na	3.556100	-0.749200	0.000000
H	-0.638700	1.005400	0.000000
H	0.822900	1.963700	0.000000
H	-0.822900	-1.963700	0.000000
H	0.638700	-1.005400	0.000000
H	-4.970300	-6.569800	0.000000
H	-3.271800	-6.211500	0.000000
H	3.271800	6.211500	0.000000
H	4.970300	6.569800	0.000000
H	1.354900	6.952800	0.000000
H	-1.354900	-6.952800	0.000000
H	-7.611200	-0.051500	0.000000
H	7.611200	0.051500	0.000000
H	-4.222100	3.717500	0.000000
H	4.222100	-3.717500	0.000000

$(K \bullet I)_2$ gas phase C_2 E = -9430.8644

N	0.552500	-4.098900	-0.520100
C	0.988300	-5.332000	-0.878900
N	2.094600	-5.642600	-1.555800

C	2.812000	-4.550600	-1.854200
C	2.498100	-3.216300	-1.535800
C	1.269900	-3.002900	-0.867300
N	4.010900	-4.479600	-2.538400
C	4.369600	-3.154900	-2.594400
N	3.493200	-2.353800	-1.999700
N	0.776000	-1.771900	-0.540800
N	-1.864500	-3.839800	0.913200
C	-2.265600	-4.917600	1.680300
N	-3.478800	-5.048700	2.195600
C	-4.291500	-4.023700	1.881000
C	-3.983500	-2.874300	1.137500
C	-2.668500	-2.735900	0.607300
N	-5.614200	-3.870500	2.233800
C	-6.048500	-2.671600	1.700300
N	-5.098600	-2.041500	1.033600
O	-2.207200	-1.776000	-0.076900
N	-1.342700	-5.912500	1.861700
N	-0.552500	4.098900	-0.520100
C	-0.988300	5.332000	-0.878900
N	-2.094600	5.642600	-1.555800
C	-2.812000	4.550600	-1.854200
C	-2.498100	3.216300	-1.535800
C	-1.269900	3.002900	-0.867300
N	-4.010900	4.479600	-2.538400
C	-4.369600	3.154900	-2.594400
N	-3.493200	2.353800	-1.999700
N	-0.776000	1.771900	-0.540800
N	1.864500	3.839800	0.913200
C	2.265600	4.917600	1.680300
N	3.478800	5.048700	2.195600
C	4.291500	4.023700	1.881000
C	3.983500	2.874300	1.137500
C	2.668500	2.735900	0.607300
N	5.614200	3.870500	2.233800
C	6.048500	2.671600	1.700300
N	5.098600	2.041500	1.033600
O	2.207200	1.776000	-0.076900
N	1.342700	5.912500	1.861700
H	0.344900	-6.159500	-0.585400
H	4.516000	-5.268800	-2.930200
H	5.278000	-2.832200	-3.087200
H	1.176000	-0.967600	-1.007100
H	-0.225700	-1.712200	-0.317600
H	-0.939600	-3.898100	0.410700
H	-6.159500	-4.527500	2.783100
H	-7.063300	-2.322300	1.836000
H	-0.357200	-5.679500	1.830700
H	-1.608300	-6.634300	2.523500
H	-0.344900	6.159500	-0.585400

H	-4.516000	5.268800	-2.930200
H	-5.278000	2.832200	-3.087200
H	-1.176000	0.967600	-1.007100
H	0.225700	1.712200	-0.317600
H	0.939600	3.898100	0.410700
H	6.159500	4.527500	2.783100
H	7.063300	2.322300	1.836000
H	0.357200	5.679500	1.830700
H	1.608300	6.634300	2.523500
K	4.276400	0.017100	-0.655200
K	-4.276400	-0.017100	-0.655200

(K • I)$_2$ gas phase C_{2h} E = -9428.0694

N	0.471300	-4.750500	0.000000
C	1.149800	-5.921800	0.000000
N	2.466600	-6.123800	0.000000
C	3.137600	-4.962200	0.000000
C	2.590200	-3.668300	0.000000
C	1.172900	-3.584300	0.000000
N	4.506600	-4.774900	0.000000
C	4.728600	-3.421000	0.000000
N	3.607300	-2.707300	0.000000
N	0.491000	-2.416200	0.000000
N	-2.458500	-4.676300	0.000000
C	-3.154000	-5.877300	0.000000
N	-4.479000	-5.966300	0.000000
C	-5.086100	-4.769100	0.000000
C	-4.487900	-3.500700	0.000000
C	-3.069000	-3.414600	0.000000
N	-6.441800	-4.518000	0.000000
C	-6.607700	-3.145300	0.000000
N	-5.457900	-2.495400	0.000000
O	-2.374300	-2.355300	0.000000
N	-2.423600	-7.019300	0.000000
N	-0.471300	4.750500	0.000000
C	-1.149800	5.921800	0.000000
N	-2.466600	6.123800	0.000000
C	-3.137600	4.962200	0.000000
C	-2.590200	3.668300	0.000000
C	-1.172900	3.584300	0.000000
N	-4.506600	4.774900	0.000000
C	-4.728600	3.421000	0.000000
N	-3.607300	2.707300	0.000000
N	-0.491000	2.416200	0.000000
N	2.458500	4.676300	0.000000
C	3.154000	5.877300	0.000000
N	4.479000	5.966300	0.000000
C	5.086100	4.769100	0.000000
C	4.487900	3.500700	0.000000

C	3.069000	3.414600	0.000000
N	6.441800	4.518000	0.000000
C	6.607700	3.145300	0.000000
N	5.457900	2.495400	0.000000
O	2.374300	2.355300	0.000000
N	2.423600	7.019300	0.000000
H	0.543500	-6.825900	0.000000
H	5.204900	-5.511600	0.000000
H	5.732500	-3.017400	0.000000
H	1.000700	-1.547400	0.000000
H	-0.538500	-2.401100	0.000000
H	-1.410400	-4.693700	0.000000
H	-7.173500	-5.221400	0.000000
H	-7.588900	-2.691200	0.000000
H	-1.416300	-7.018500	0.000000
H	-2.917600	-7.901900	0.000000
H	-0.543500	6.825900	0.000000
H	-5.204900	5.511600	0.000000
H	-5.732500	3.017400	0.000000
H	-1.000700	1.547400	0.000000
H	0.538500	2.401100	0.000000
H	1.410400	4.693700	0.000000
H	7.173500	5.221400	0.000000
H	7.588900	2.691200	0.000000
H	1.416300	7.018500	0.000000
H	2.917600	7.901900	0.000000
K	4.008200	0.141200	0.000000
K	-4.008200	-0.141200	0.000000

(K • I)$_2$ water C$_2$ E = -9585.0355

N	1.123800	-2.349400	0.363400
C	1.957300	-2.942500	1.246000
N	3.231300	-3.313100	1.067200
C	3.664500	-3.009300	-0.175500
C	2.917600	-2.391200	-1.188500
C	1.570100	-2.072000	-0.896600
N	4.905700	-3.228400	-0.733100
C	4.864500	-2.745600	-2.019100
N	3.685100	-2.226500	-2.339400
N	0.733600	-1.509700	-1.790000
N	-1.445400	-1.371200	1.087100
C	-1.680400	-1.062900	2.408900
N	-2.796800	-0.489500	2.851400
C	-3.715800	-0.303600	1.873300
C	-3.593600	-0.630500	0.516600
C	-2.370200	-1.184400	0.050200
N	-4.972200	0.239000	1.992300
C	-5.553100	0.212200	0.737700
N	-4.754500	-0.303500	-0.179700

O	-2.075500	-1.485500	-1.136400
N	-0.711300	-1.424200	3.304100
N	-1.123800	2.349400	0.363400
C	-1.957300	2.942500	1.246000
N	-3.231300	3.313100	1.067200
C	-3.664500	3.009300	-0.175500
C	-2.917600	2.391200	-1.188500
C	-1.570100	2.072000	-0.896600
N	-4.905700	3.228400	-0.733100
C	-4.864500	2.745600	-2.019100
N	-3.685100	2.226500	-2.339400
N	-0.733600	1.509700	-1.790000
N	1.445400	1.371200	1.087100
C	1.680400	1.062900	2.408900
N	2.796800	0.489500	2.851400
C	3.715800	0.303600	1.873300
C	3.593600	0.630500	0.516600
C	2.370200	1.184400	0.050200
N	4.972200	-0.239000	1.992300
C	5.553100	-0.212200	0.737700
N	4.754500	0.303500	-0.179700
O	2.075500	1.485500	-1.136400
N	0.711300	1.424200	3.304100
H	1.529100	-3.159600	2.222200
H	5.695400	-3.681600	-0.286600
H	5.723700	-2.797300	-2.672500
H	1.039400	-1.425100	-2.750800
H	-0.273900	-1.447300	-1.594600
H	-0.516800	-1.796700	0.824900
H	-5.401700	0.580200	2.845500
H	-6.552700	0.585700	0.569200
H	0.244900	-1.522900	2.985400
H	-0.816000	-1.058700	4.243600
H	-1.529100	3.159600	2.222200
H	-5.695400	3.681600	-0.286600
H	-5.723700	2.797300	-2.672500
H	-1.039400	1.425100	-2.750800
H	0.273900	1.447300	-1.594600
H	0.516800	1.796700	0.824900
H	5.401700	-0.580200	2.845500
H	6.552700	-0.585700	0.569200
H	-0.244900	1.522900	2.985400
H	0.816000	1.058700	4.243600
K	4.093300	0.672300	-2.988800
K	-4.093300	-0.672300	-2.988800

(K • I)$_2$ water C$_{2h}$ E = -9571.9555

N	0.460600	-4.840100	0.000000
C	1.136300	-6.008100	0.000000

N	2.460700	-6.211400	0.000000
C	3.138300	-5.041800	0.000000
C	2.578700	-3.754400	0.000000
C	1.163300	-3.667500	0.000000
N	4.501400	-4.841300	0.000000
C	4.711500	-3.484300	0.000000
N	3.582100	-2.785100	0.000000
N	0.489700	-2.502800	0.000000
N	-2.470900	-4.781000	0.000000
C	-3.171600	-5.972800	0.000000
N	-4.505000	-6.048600	0.000000
C	-5.102300	-4.835600	0.000000
C	-4.484800	-3.578000	0.000000
C	-3.065300	-3.510100	0.000000
N	-6.451100	-4.565500	0.000000
C	-6.596700	-3.190600	0.000000
N	-5.436400	-2.557300	0.000000
O	-2.358800	-2.464600	0.000000
N	-2.446600	-7.115200	0.000000
N	-0.460600	4.840100	0.000000
C	-1.136300	6.008100	0.000000
N	-2.460700	6.211400	0.000000
C	-3.138300	5.041800	0.000000
C	-2.578700	3.754400	0.000000
C	-1.163300	3.667500	0.000000
N	-4.501400	4.841300	0.000000
C	-4.711500	3.484300	0.000000
N	-3.582100	2.785100	0.000000
N	-0.489700	2.502800	0.000000
N	2.470900	4.781000	0.000000
C	3.171600	5.972800	0.000000
N	4.505000	6.048600	0.000000
C	5.102300	4.835600	0.000000
C	4.484800	3.578000	0.000000
C	3.065300	3.510100	0.000000
N	6.451100	4.565500	0.000000
C	6.596700	3.190600	0.000000
N	5.436400	2.557300	0.000000
O	2.358800	2.464600	0.000000
N	2.446600	7.115200	0.000000
H	0.527300	-6.908700	0.000000
H	5.217300	-5.559500	0.000000
H	5.708200	-3.067200	0.000000
H	0.999000	-1.631200	0.000000
H	-0.539200	-2.484400	0.000000
H	-1.424300	-4.806500	0.000000
H	-7.200900	-5.248600	0.000000
H	-7.571400	-2.725000	0.000000
H	-1.438500	-7.113300	0.000000
H	-2.927300	-8.004500	0.000000

H	-0.527300	6.908700	0.000000
H	-5.217300	5.559500	0.000000
H	-5.708200	3.067200	0.000000
H	-0.999000	1.631200	0.000000
H	0.539200	2.484400	0.000000
H	1.424300	4.806500	0.000000
H	7.200900	5.248600	0.000000
H	7.571400	2.725000	0.000000
H	1.438500	7.113300	0.000000
H	2.927300	8.004500	0.000000
K	3.967400	0.076100	0.000000
K	-3.967400	-0.076100	0.000000

$(K \bullet II)_2$ gas phase C_2 E = -9432.8127

N	-2.653300	-2.992700	-0.702600
C	-3.173900	-3.987500	-1.511900
N	-4.044600	-3.765500	-2.486700
C	-4.401000	-2.472600	-2.584700
C	-3.937800	-1.387600	-1.823700
C	-2.958600	-1.629100	-0.817800
N	-5.305600	-1.922200	-3.466500
C	-5.368500	-0.566600	-3.202300
N	-4.561700	-0.204100	-2.221300
O	-2.392300	-0.786900	-0.067200
N	-2.775100	-5.263500	-1.242500
H	-5.830300	-2.431500	-4.171100
H	-2.070800	-3.282100	0.120300
N	2.653300	2.992700	-0.702600
C	3.173900	3.987500	-1.511900
N	4.044600	3.765500	-2.486700
C	4.401000	2.472600	-2.584700
C	3.937800	1.387600	-1.823700
C	2.958600	1.629100	-0.817800
N	5.305600	1.922200	-3.466500
C	5.368500	0.566600	-3.202300
N	4.561700	0.204100	-2.221300
O	2.392300	0.786900	-0.067200
N	2.775100	5.263500	-1.242500
H	5.830300	2.431500	-4.171100
H	2.070800	3.282100	0.120300
N	-2.366600	2.654700	1.631100
C	-2.766700	3.594000	2.528400
N	-2.044400	4.559800	3.099500
C	-0.781000	4.557300	2.650500
C	-0.223900	3.668800	1.716500
C	-1.064100	2.636000	1.236100
N	0.249500	5.418300	2.984700
C	1.349700	5.041500	2.252000
N	1.107500	3.996300	1.473000

N	-0.638500	1.660800	0.389300
H	0.191300	6.179400	3.654000
K	-4.307800	1.525300	-0.064100
N	2.366600	-2.654700	1.631100
C	2.766700	-3.594000	2.528400
N	2.044400	-4.559800	3.099500
C	0.781000	-4.557300	2.650500
C	0.223900	-3.668800	1.716500
C	1.064100	-2.636000	1.236100
N	-0.249500	-5.418300	2.984700
C	-1.349700	-5.041500	2.252000
N	-1.107500	-3.996300	1.473000
N	0.638500	-1.660800	0.389300
H	-0.191300	-6.179400	3.654000
K	4.307800	-1.525300	-0.064100
H	-1.236300	0.845900	0.251700
H	0.363300	1.502800	0.285300
H	-0.363300	-1.502800	0.285300
H	1.236300	-0.845900	0.251700
H	-3.054800	-5.973200	-1.909300
H	-1.930800	-5.427100	-0.708600
H	1.930800	5.427100	-0.708600
H	3.054800	5.973200	-1.909300
H	2.295800	5.561700	2.321300
H	-2.295800	-5.561700	2.321300
H	-6.022200	0.092700	-3.758000
H	6.022200	-0.092700	-3.758000
H	-3.813400	3.546600	2.825800
H	3.813400	-3.546600	2.825800

$(K \bullet II)_2$ gas phase C_{2h} E = -9427.3233

N	-3.732900	-4.019100	0.000000
C	-4.781200	-4.928700	0.000000
N	-6.061300	-4.585000	0.000000
C	-6.250300	-3.255500	0.000000
C	-5.273600	-2.250300	0.000000
C	-3.902600	-2.626900	0.000000
N	-7.451000	-2.580300	0.000000
C	-7.163600	-1.227500	0.000000
N	-5.865700	-0.986300	0.000000
O	-2.903400	-1.853000	0.000000
N	-4.458600	-6.246600	0.000000
H	-8.371500	-3.008400	0.000000
H	-2.754000	-4.377500	0.000000
N	3.732900	4.019100	0.000000
C	4.781200	4.928700	0.000000
N	6.061300	4.585000	0.000000
C	6.250300	3.255500	0.000000
C	5.273600	2.250300	0.000000

C	3.902600	2.626900	0.000000
N	7.451000	2.580300	0.000000
C	7.163600	1.227500	0.000000
N	5.865700	0.986300	0.000000
O	2.903400	1.853000	0.000000
N	4.458600	6.246600	0.000000
H	8.371500	3.008400	0.000000
H	2.754000	4.377500	0.000000
N	-2.074700	3.103500	0.000000
C	-2.901700	4.180800	0.000000
N	-2.584500	5.473200	0.000000
C	-1.255000	5.650400	0.000000
C	-0.264600	4.652200	0.000000
C	-0.727600	3.310800	0.000000
N	-0.564700	6.845200	0.000000
C	0.773000	6.536000	0.000000
N	1.006900	5.231900	0.000000
N	0.095400	2.238600	0.000000
H	-0.981800	7.770800	0.000000
K	-3.750600	0.794100	0.000000
N	2.074700	-3.103500	0.000000
C	2.901700	-4.180800	0.000000
N	2.584500	-5.473200	0.000000
C	1.255000	-5.650400	0.000000
C	0.264600	-4.652200	0.000000
C	0.727600	-3.310800	0.000000
N	0.564700	-6.845200	0.000000
C	-0.773000	-6.536000	0.000000
N	-1.006900	-5.231900	0.000000
N	-0.095400	-2.238600	0.000000
H	0.981800	-7.770800	0.000000
K	3.750600	-0.794100	0.000000
H	-0.325700	1.320700	0.000000
H	1.121200	2.299300	0.000000
H	-1.121200	-2.299300	0.000000
H	0.325700	-1.320700	0.000000
H	-5.210800	-6.923100	0.000000
H	-3.504000	-6.563800	0.000000
H	3.504000	6.563800	0.000000
H	5.210800	6.923100	0.000000
H	1.520000	7.318900	0.000000
H	-1.520000	-7.318900	0.000000
H	-7.944800	-0.479200	0.000000
H	7.944800	0.479200	0.000000
H	-3.969900	3.962400	0.000000
H	3.969900	-3.962400	0.000000

(K • II)$_2$ water C$_2$ E = -9579.6430

N	-2.761700	-3.023000	-0.490300

C	-3.404100	-4.061300	-1.128200
N	-4.270800	-3.889900	-2.125200
C	-4.458600	-2.587600	-2.438000
C	-3.842700	-1.466100	-1.861800
C	-2.911100	-1.667600	-0.806200
N	-5.294800	-2.075400	-3.401000
C	-5.168100	-0.701500	-3.370500
N	-4.302300	-0.292600	-2.458400
O	-2.256300	-0.798800	-0.166500
N	-3.152300	-5.316500	-0.655400
H	-5.898600	-2.608700	-4.017700
H	-2.148700	-3.251400	0.329500
N	2.761700	3.023000	-0.490300
C	3.404100	4.061300	-1.128200
N	4.270800	3.889900	-2.125200
C	4.458600	2.587600	-2.438000
C	3.842700	1.466100	-1.861800
C	2.911100	1.667600	-0.806200
N	5.294800	2.075400	-3.401000
C	5.168100	0.701500	-3.370500
N	4.302300	0.292600	-2.458400
O	2.256300	0.798800	-0.166500
N	3.152300	5.316500	-0.655400
H	5.898600	2.608700	-4.017700
H	2.148700	3.251400	0.329500
N	-2.429300	2.618200	1.447900
C	-2.884700	3.539700	2.325200
N	-2.190100	4.478100	2.988200
C	-0.881000	4.445100	2.662500
C	-0.272000	3.557900	1.761800
C	-1.097700	2.581200	1.155700
N	0.139400	5.255300	3.116100
C	1.291700	4.849700	2.492600
N	1.090700	3.829200	1.668300
N	-0.640700	1.644700	0.293200
H	0.056000	6.013100	3.785200
K	-4.351700	1.743300	-0.495200
N	2.429300	-2.618200	1.447900
C	2.884700	-3.539700	2.325200
N	2.190100	-4.478100	2.988200
C	0.881000	-4.445100	2.662500
C	0.272000	-3.557900	1.761800
C	1.097700	-2.581200	1.155700
N	-0.139400	-5.255300	3.116100
C	-1.291700	-4.849700	2.492600
N	-1.090700	-3.829200	1.668300
N	0.640700	-1.644700	0.293200
H	-0.056000	-6.013100	3.785200
K	4.351700	-1.743300	-0.495200
H	-1.254200	0.866900	0.048100

H	0.362900	1.478800	0.206800
H	-0.362900	-1.478800	0.206800
H	1.254200	-0.866900	0.048100
H	-3.460200	-6.085100	-1.240700
H	-2.294100	-5.476700	-0.139400
H	2.294100	5.476700	-0.139400
H	3.460200	6.085100	-1.240700
H	2.242400	5.331300	2.670900
H	-2.242400	-5.331300	2.670900
H	-5.735200	-0.063900	-4.033600
H	5.735200	0.063900	-4.033600
H	-3.955300	3.513700	2.517400
H	3.955300	-3.513700	2.517400

(K • II)$_2$ water C$_{2h}$ E = -9571.6847

N	-3.771600	-4.133200	0.000000
C	-4.827600	-5.026600	0.000000
N	-6.110600	-4.657700	0.000000
C	-6.274300	-3.315600	0.000000
C	-5.275600	-2.333300	0.000000
C	-3.913000	-2.738500	0.000000
N	-7.458200	-2.615000	0.000000
C	-7.141300	-1.269600	0.000000
N	-5.837000	-1.055700	0.000000
O	-2.900200	-1.987100	0.000000
N	-4.520100	-6.343800	0.000000
H	-8.391900	-3.011300	0.000000
H	-2.795300	-4.501500	0.000000
N	3.771600	4.133200	0.000000
C	4.827600	5.026600	0.000000
N	6.110600	4.657700	0.000000
C	6.274300	3.315600	0.000000
C	5.275600	2.333300	0.000000
C	3.913000	2.738500	0.000000
N	7.458200	2.615000	0.000000
C	7.141300	1.269600	0.000000
N	5.837000	1.055700	0.000000
O	2.900200	1.987100	0.000000
N	4.520100	6.343800	0.000000
H	8.391900	3.011300	0.000000
H	2.795300	4.501500	0.000000
N	-2.063100	3.212200	0.000000
C	-2.874200	4.292300	0.000000
N	-2.537400	5.591000	0.000000
C	-1.197000	5.752300	0.000000
C	-0.229000	4.733600	0.000000
C	-0.710200	3.397800	0.000000
N	-0.483200	6.931100	0.000000
C	0.846400	6.599600	0.000000

N	1.054200	5.289000	0.000000
N	0.095200	2.319000	0.000000
H	-0.867400	7.869600	0.000000
K	-3.655400	0.783700	0.000000
N	2.063100	-3.212200	0.000000
C	2.874200	-4.292300	0.000000
N	2.537400	-5.591000	0.000000
C	1.197000	-5.752300	0.000000
C	0.229000	-4.733600	0.000000
C	0.710200	-3.397800	0.000000
N	0.483200	-6.931100	0.000000
C	-0.846400	-6.599600	0.000000
N	-1.054200	-5.289000	0.000000
N	-0.095200	-2.319000	0.000000
H	0.867400	-7.869600	0.000000
K	3.655400	-0.783700	0.000000
H	-0.324200	1.399000	0.000000
H	1.120100	2.383000	0.000000
H	-1.120100	-2.383000	0.000000
H	0.324200	-1.399000	0.000000
H	-5.265800	-7.026200	0.000000
H	-3.567500	-6.669900	0.000000
H	3.567500	6.669900	0.000000
H	5.265800	7.026200	0.000000
H	1.607500	7.366200	0.000000
H	-1.607500	-7.366200	0.000000
H	-7.906500	-0.507200	0.000000
H	7.906500	0.507200	0.000000
H	-3.940900	4.079800	0.000000
H	3.940900	-4.079800	0.000000

Guanine gas phase C_1 E = -2456.1560

N	2.830700	1.694000	0.078200
C	3.899700	0.828700	0.041200
N	3.770500	-0.482000	0.005300
C	2.470100	-0.882800	0.020900
C	1.301600	-0.106300	0.042700
C	1.420700	1.330400	0.079800
N	2.029000	-2.186700	-0.000800
C	0.637500	-2.140800	0.009500
N	0.171200	-0.912500	0.035700
O	0.575900	2.215000	0.116600
N	5.168000	1.386100	0.098400
H	2.622200	-3.007400	-0.019900
H	0.042200	-3.043700	-0.005200
H	2.987000	2.694800	0.170100
H	5.904100	0.716200	-0.100200
H	5.301700	2.286100	-0.350000

Guanine water C_1 E = -2481.3570

N	2.825800	1.695000	0.075600
C	3.903700	0.832800	0.041200
N	3.769100	-0.490000	0.010100
C	2.474100	-0.892500	0.019700
C	1.315800	-0.099600	0.050000
C	1.455800	1.321700	0.081400
N	2.016400	-2.188000	-0.005800
C	0.635700	-2.135900	0.008500
N	0.172600	-0.898000	0.042200
O	0.570800	2.200600	0.111200
N	5.146900	1.389700	0.090400
H	2.587500	-3.025700	-0.032000
H	0.036600	-3.034900	-0.009400
H	3.000100	2.696900	0.110100
H	5.922300	0.775900	-0.133000
H	5.265300	2.360400	-0.175800

Adenine gas phase C_1 E = -2299.8969

N	2.873500	1.759200	0.053300
C	3.867200	0.846200	0.043900
N	3.778100	-0.495700	0.029200
C	2.496200	-0.897100	0.023100
C	1.356500	-0.074200	0.033500
C	1.596200	1.319300	0.051700
N	1.992800	-2.186800	0.000500
C	0.609400	-2.077400	-0.004100
N	0.186100	-0.829200	0.015100
N	0.583600	2.230800	0.089100
H	4.875100	1.255300	0.047000
H	2.543400	-3.036500	-0.013400
H	-0.025400	-2.953500	-0.021700
H	-0.367600	1.919400	-0.051800
H	0.810900	3.208100	-0.037600

Adenine water C_1 E = -2314.8756

N	2.877700	1.760800	0.063000
C	3.871000	0.846900	0.047800
N	3.777800	-0.494900	0.025300
C	2.489600	-0.901400	0.018000
C	1.355700	-0.071800	0.030100
C	1.589000	1.324600	0.053700
N	1.989700	-2.186200	-0.000900
C	0.616100	-2.086200	0.000900
N	0.186300	-0.832200	0.019400
N	0.587200	2.234500	0.048300
H	4.879400	1.253900	0.055200

H	2.529100	-3.044700	-0.011900
H	-0.013100	-2.964800	-0.012700
H	-0.374000	1.934600	0.143300
H	0.799100	3.217500	0.165900

Adeninium (N1H) gas phase C_1 E = -2243.7404

N	2.870400	1.710700	0.058700
C	3.930300	0.808700	0.046000
N	3.790700	-0.487600	0.026400
C	2.497700	-0.889600	0.019300
C	1.355600	-0.065000	0.030800
C	1.545800	1.329500	0.051900
N	1.999200	-2.164500	-0.000300
C	0.614200	-2.056600	0.000200
N	0.194000	-0.806000	0.018700
N	0.550400	2.222400	0.064500
H	4.917900	1.259400	0.053600
H	2.548600	-3.019600	-0.012800
H	-0.023000	-2.931200	-0.013500
H	-0.404900	1.878000	0.058600
H	0.696100	3.224600	0.079800
H	3.104500	2.701800	0.073800

Adeninium (N1H) water C_1 E = -2309.5304

N	2.866800	1.706300	0.060900
C	3.916700	0.819900	0.045900
N	3.786700	-0.485400	0.025200
C	2.489100	-0.887900	0.019700
C	1.350000	-0.065800	0.032800
C	1.543800	1.331700	0.052700
N	1.999000	-2.165900	-0.001400
C	0.624900	-2.074000	-0.000400
N	0.189900	-0.821700	0.020000
N	0.573800	2.240400	0.061600
H	4.899400	1.274200	0.051800
H	2.545800	-3.020700	-0.015600
H	-0.000400	-2.954600	-0.016200
H	-0.392100	1.936500	0.062300
H	0.762300	3.235400	0.076900
H	3.093100	2.698400	0.076800

Adeninium (N7H) gas phase C_1 E = -2235.9920

N	2.868400	1.753600	0.059400
C	3.854900	0.843800	0.046500
N	3.741800	-0.501300	0.026100
C	2.470600	-0.877500	0.019400
C	1.342900	-0.042400	0.031100

C	1.579700	1.360900	0.052400
N	1.979500	-2.190900	-0.000700
C	0.637600	-2.166700	-0.001500
N	0.225200	-0.889200	0.017400
N	0.619700	2.310900	0.065900
H	4.866700	1.239900	0.053300
H	2.558500	-3.027600	-0.012900
H	-0.009400	-3.031800	-0.015000
H	-0.369400	2.108000	0.062100
H	0.911800	3.282700	0.080800
H	-0.753800	-0.620100	0.020700

Adeninium (N7H) water C_1 E = -2305.3720

N	2.877400	1.763000	0.061300
C	3.860000	0.843200	0.048000
N	3.753400	-0.499100	0.024700
C	2.469900	-0.889500	0.018800
C	1.357600	-0.044800	0.033800
C	1.582400	1.354200	0.053100
N	1.969500	-2.186400	-0.003400
C	0.627600	-2.141300	-0.001600
N	0.233000	-0.864900	0.019800
N	0.599900	2.271300	0.061900
H	4.871700	1.239100	0.055500
H	2.520100	-3.039000	-0.015500
H	-0.026400	-2.997200	-0.008700
H	-0.377200	2.012500	0.055000
H	0.834000	3.256800	0.073000
H	-0.739000	-0.574600	0.034400
